# Supplementary material for: Runx1 and Runx2 act in concert to suppress Wnt/β-catenin-driven mammary tumourigenesis
Source: Br J Cancer. 2026 May 7;135(4):532–45. doi: 10.1038/s41416-026-03439-5 (PMC13427844; doi:10.1038/s41416-026-03439-5)
Supplement: Supplementary file 1 — Supplementary Figures [file 41416_2026_3439_MOESM1_ESM.pdf]

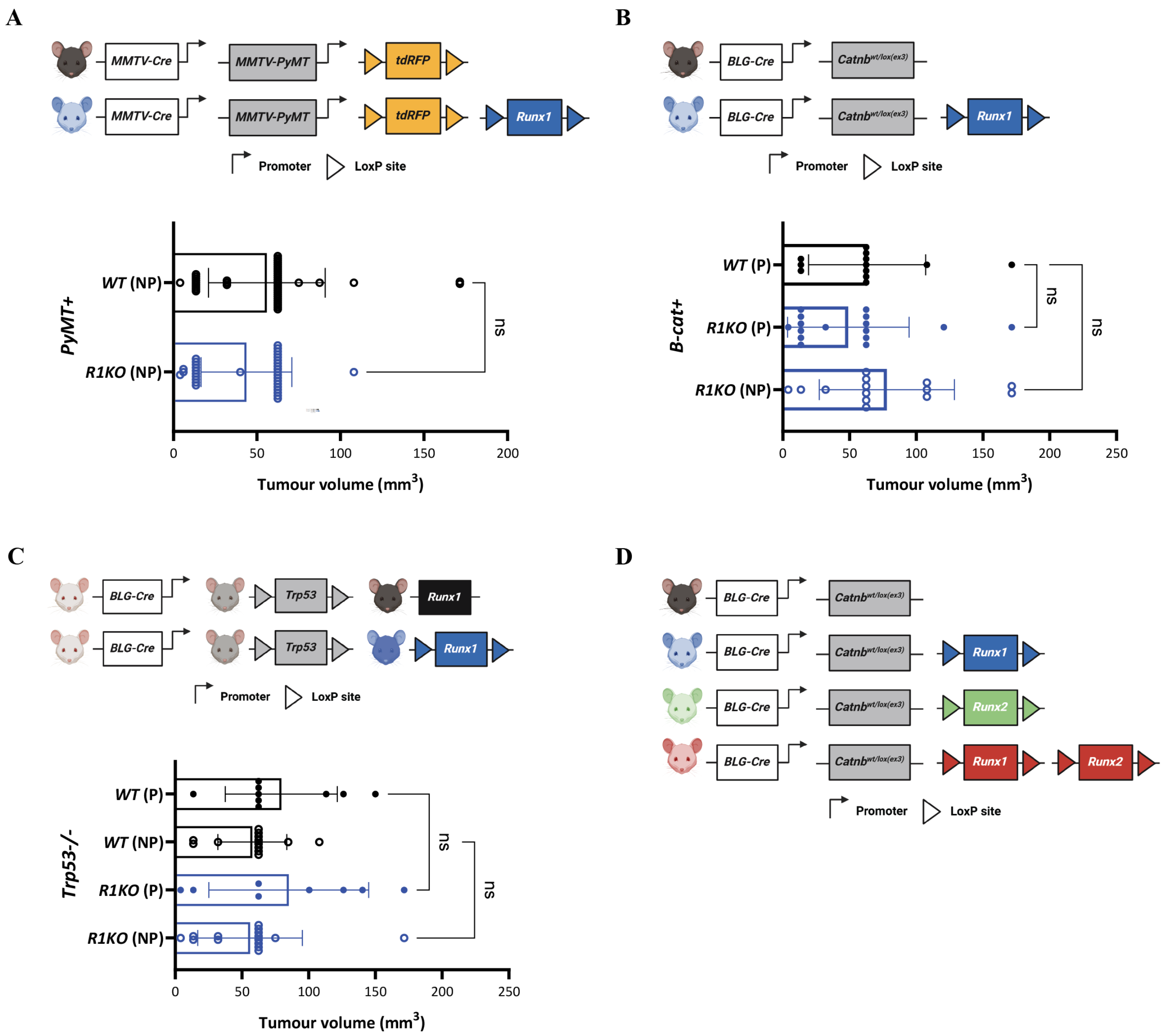

Supplementary Figure 1 (relative to Fig. 1 and Fig 2A).

A. Schematic of mouse cohorts (top) and tumour volume (bottom) of *PyMT+* cohorts as in Fig. 1A (*n* = 44; *WT*; *n* = 34; *R1KO*) at date of tumour notice. Average tumour volume with SD shown: 56mm^3 (±34.9) for *WT* and 44mm^3 (±27.2) for *R1KO*. NP, nulliparous. Statistical analysis performed with two-tailed unpaired t-test; ns, non-significant ($P > 0.05$).

B. Schematic of mouse cohorts (top) and tumour volume (bottom) of *B-cat+* cohorts as in Fig. 1C (*n* = 12, *B-cat+/WT*-parous; *n* = 16, *B-cat+/R1KO*-parous; *n* = 14, *B-cat+/R1KO*-nulliparous) at date of tumour notice. Average tumour volume with SD shown: 63mm^3 (±43.8) for *WT*-parous, 49mm^3 (±45.5) for *R1KO*-parous and 78mm^3 (±50.7) for *R1KO*-nulliparous. Statistical analysis performed with ordinary one-way ANOVA test with Dunnett's multiple comparisons test; ns, non-significant ($P > 0.05$).

C. Schematic of mouse cohorts (top) and tumour volume (bottom) of *BLG-Cre;*$Trp53^{fl/fl}$ cohorts as in Fig. 1D (*n* = 9, *WT*-P; *n* = 13, *WT*-NP; *n* = 8, *R1KO*-P; *n* = 15, *R1KO*-NP) at dates of tumour notice. Average tumour volume with SD shown. Parous (P) and nulliparous (NP) *WT* mice: 79mm^3 (±41.9) and 58mm^3 (±25.8), respectively. *R1KO*-P and *R1KO*-NP mice: 85mm^3 (±59.9) and 56mm^3 (±39.2), respectively. Statistical analysis performed with ordinary one-way ANOVA test with Sidak's multiple comparisons test; ns, non-significant ($P > 0.05$).

D. Schematic of mouse cohorts shown in Fig. 2A.

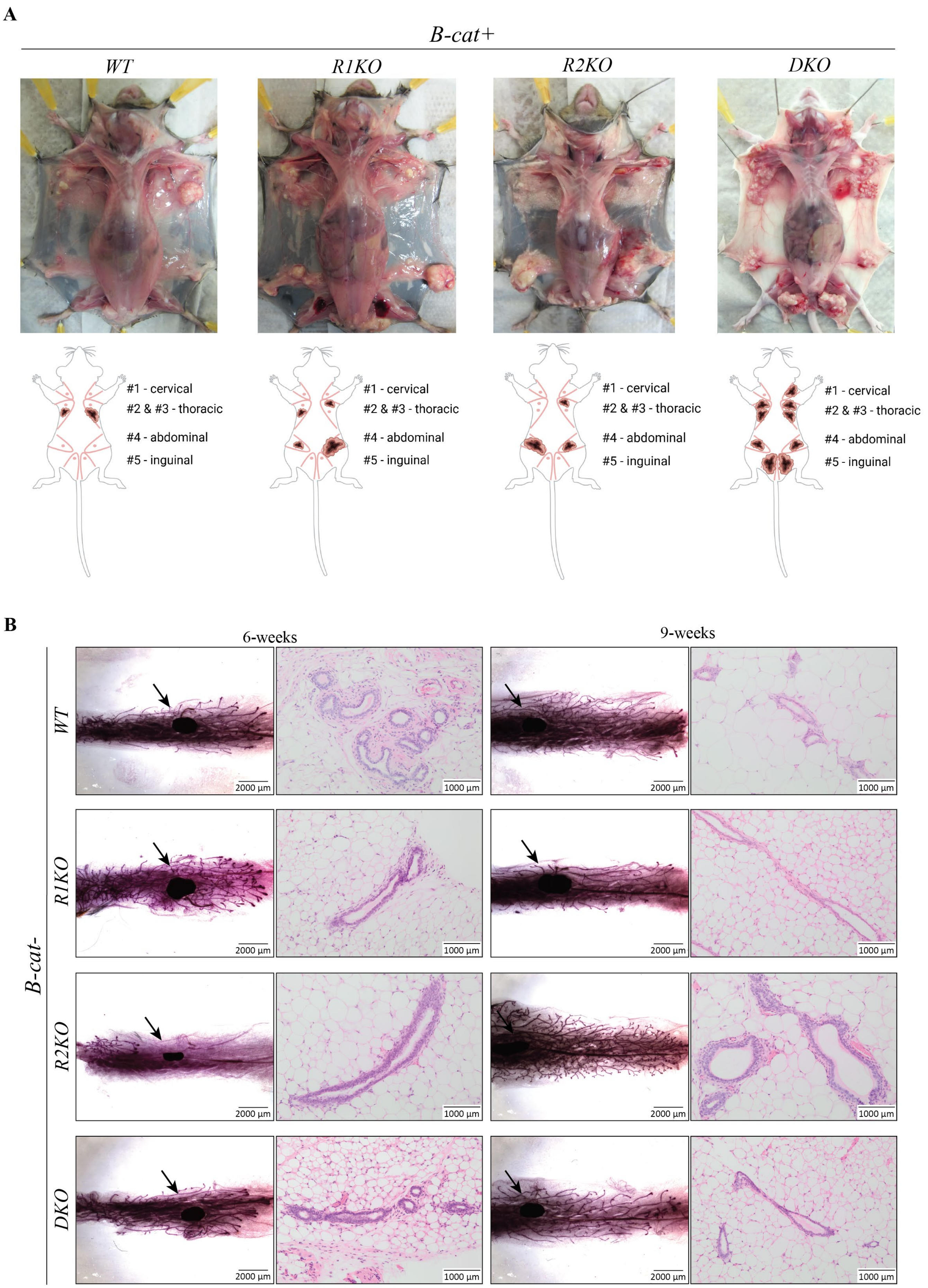
A
B-cat+
WT
R1KO
R2KO
DKO
#1 - cervical
#2 & #3 - thoracic
#4 - abdominal
#5 - inguinal
B
6-weeks
9-weeks
B-cat-
WT
R1KO
R2KO
DKO
2000 µm
1000 µm

Supplementary Figure 2 (relative to Fig. 2).

A. One representative picture shown per genotype ($n \geq 9$ mice per genotype) with diagrams below illustrating mammary gland location and necropsy examination of *B-cat+* cohorts at clinical endpoint (as in Fig. 2B). Note the distinct formation of multiple, sometimes coalescing tumours affecting almost all mammary glands in nulliparous *B-cat+/DKO* mice compared to other genotypes (as taken from parous cohorts).

B. Wholemount and H&E images of abdominal mammary glands from 6- and 9-weeks old *BLG-Cre;Runx1*$^{wt/wt}$ (*WT*; $n = 5$ for 6-weeks; $n = 6$ for 9-weeks), *Runx1*$^{fl/fl}$ (*R1KO*; $n = 7$ for 6-weeks; $n = 7$ for 9-weeks), *Runx2*$^{fl/fl}$ (*R2KO*; $n = 4$ for 6-weeks; $n = 5$ for 9-weeks) and *Runx1*$^{fl/fl}$*;Runx2*$^{fl/fl}$ (*DKO*; $n = 7$ for 6-weeks; $n = 8$ for 9-weeks) cohorts (all without *Ctnnb1*$^{wt/lox(ex3)}$, *B-cat-*). One representative wholemount and H&E shown per genotype. Scale bars of wholemounts, 2000µm; H&E images, 1000µm. Arrow depicts lymph node.

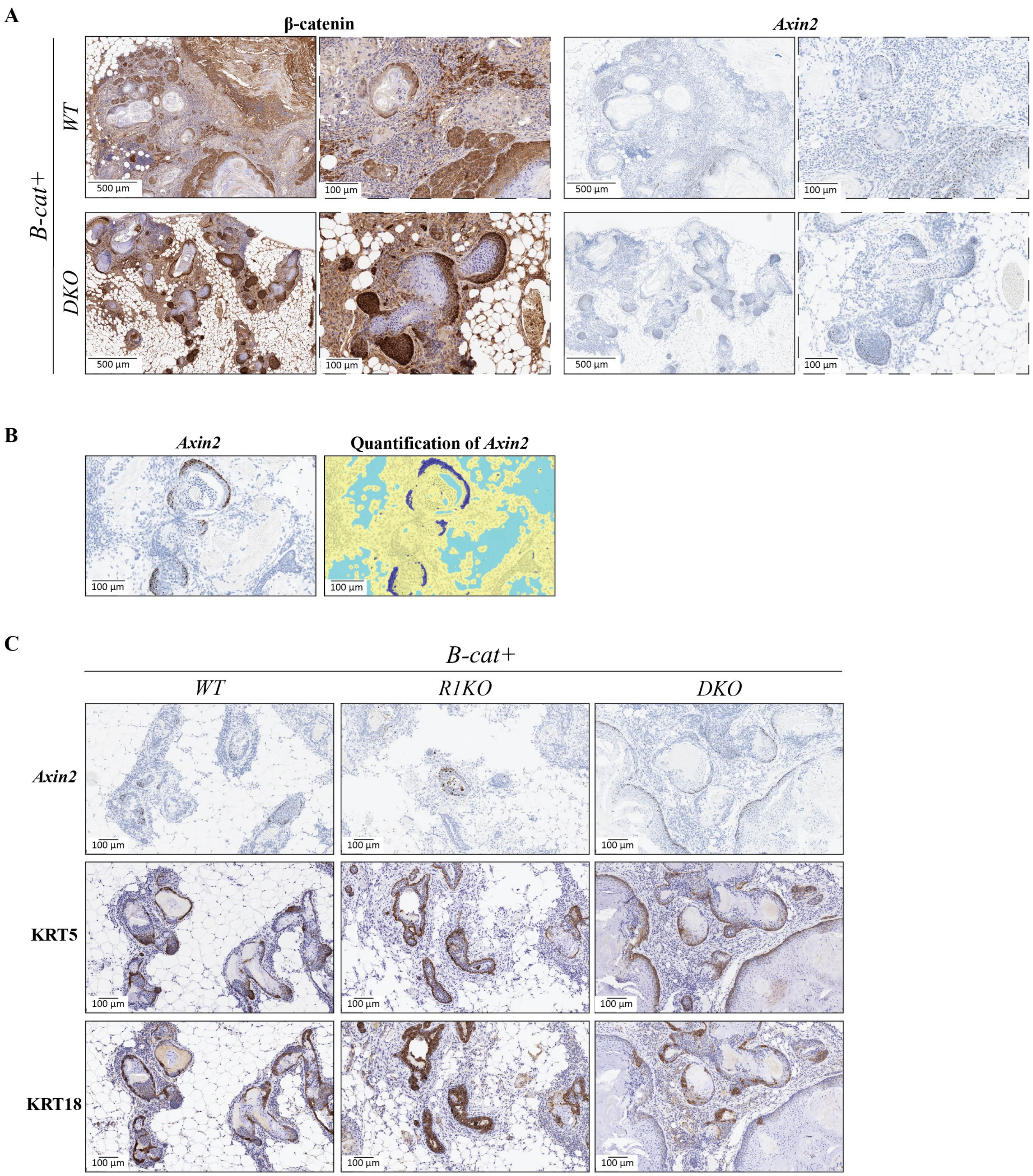

Supplementary Figure 3 (relative to Fig. 2D).

A. Representative IHC images for β-catenin (left) and ISH staining (right) for *Axin2* in 9-weeks mammary glands from *B-cat+/WT* (representative of *n* = 3 mice; top) and *B-cat+/DKO* (representative of *n* = 3; bottom). Serial sections from the same mouse used to stain for β-catenin and *Axin2*. Note *Axin2* positivity overlaps with areas of nuclear β-catenin. Scale bars, 500µm and 100µm. Dotted box is higher magnification of images directly to the left.

B. Representation of the quantification of positive *Axin2* ISH staining. Positive stain (blue), negative tissue (yellow), and background (cyan) were classified using the Random Forest machine learning algorithm in HALO software (see methods). Relative to the quantification shown in Fig. 2D.

C. Serial sections stained for *Axin2* (ISH, top), KRT5 (IHC, middle) and KRT18 (IHC, bottom) of 9-weeks old mammary glands from *B-cat+/WT*, *B-cat+/R1KO* and *B-cat+/DKO* mice. One representative image is shown per genotype (of $n = 5$ mice per genotype). Scale bars, 100µm. High power magnification of the same images are shown in Fig. 2D.

A

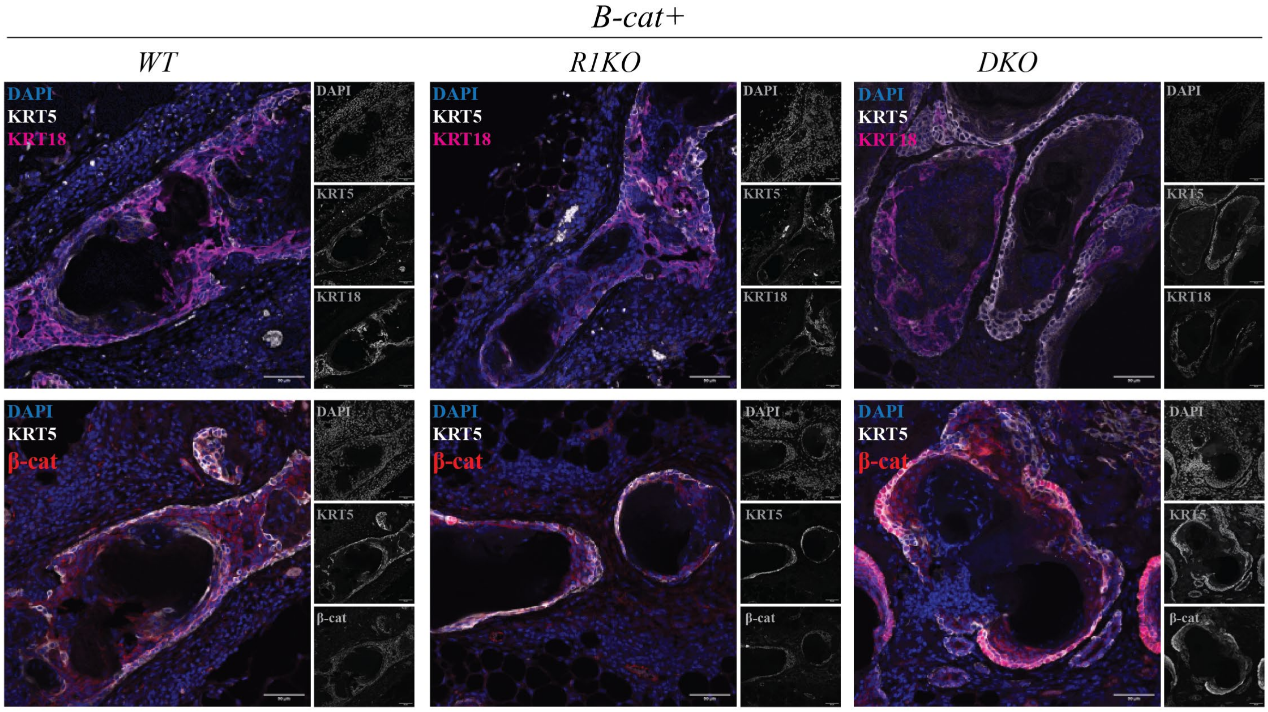

B

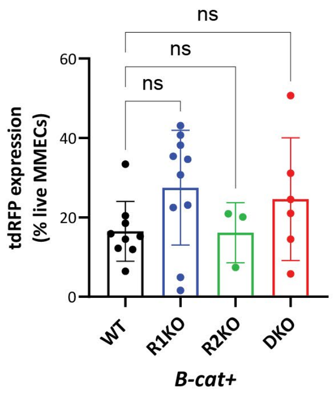

Supplementary Figure 4.

A. Top: Co-IF images of 9-weeks glands from *B-cat+/WT, B-cat+/R1KO* and *B-cat+/DKO* mice. Samples stained with KERATIN-5 (KRT5, white) and KERATIN 18 (KRT18, magenta) and DAPI (blue). KRT5 brightness increased for visualisation purposes. One representative image is shown per genotype (of *n* = 2 mice per genotype). Bottom: Co-IF of 9-weeks glands from *B-cat+/WT, B-cat+/R1KO* and *B-cat+/DKO* mice. Samples stained with KERATIN-5 (KRT5, white) and β-catenin (β-cat, red) and DAPI (blue). KRT5 brightness increased for visualisation purposes. One representative image is shown per genotype (of *n* = 2 mice per genotype).

B. Flow cytometric analysis of *tdRFP* positivity in MMECs from *B-cat+/WT* (*n* = 9), *B-cat+/R1KO* (*n* = 10), *B-cat+/R2KO* (*n* = 3) and *B-cat+/DKO* (*n* = 6) female mice at 8-9-weeks of age. Each data point represents percentage of *tdRFP*-positive MMECs, defined as DAPI-/CD45-/CD31-/CD24+, isolated from one mouse, with ten glands analysed per animal. Bars represent mean $\pm$ S.D. Statistical analysis performed with ordinary one-way ANOVA with Dunnett's multiple comparisons test; ns, non-significant (ns = $P > 0.05$).

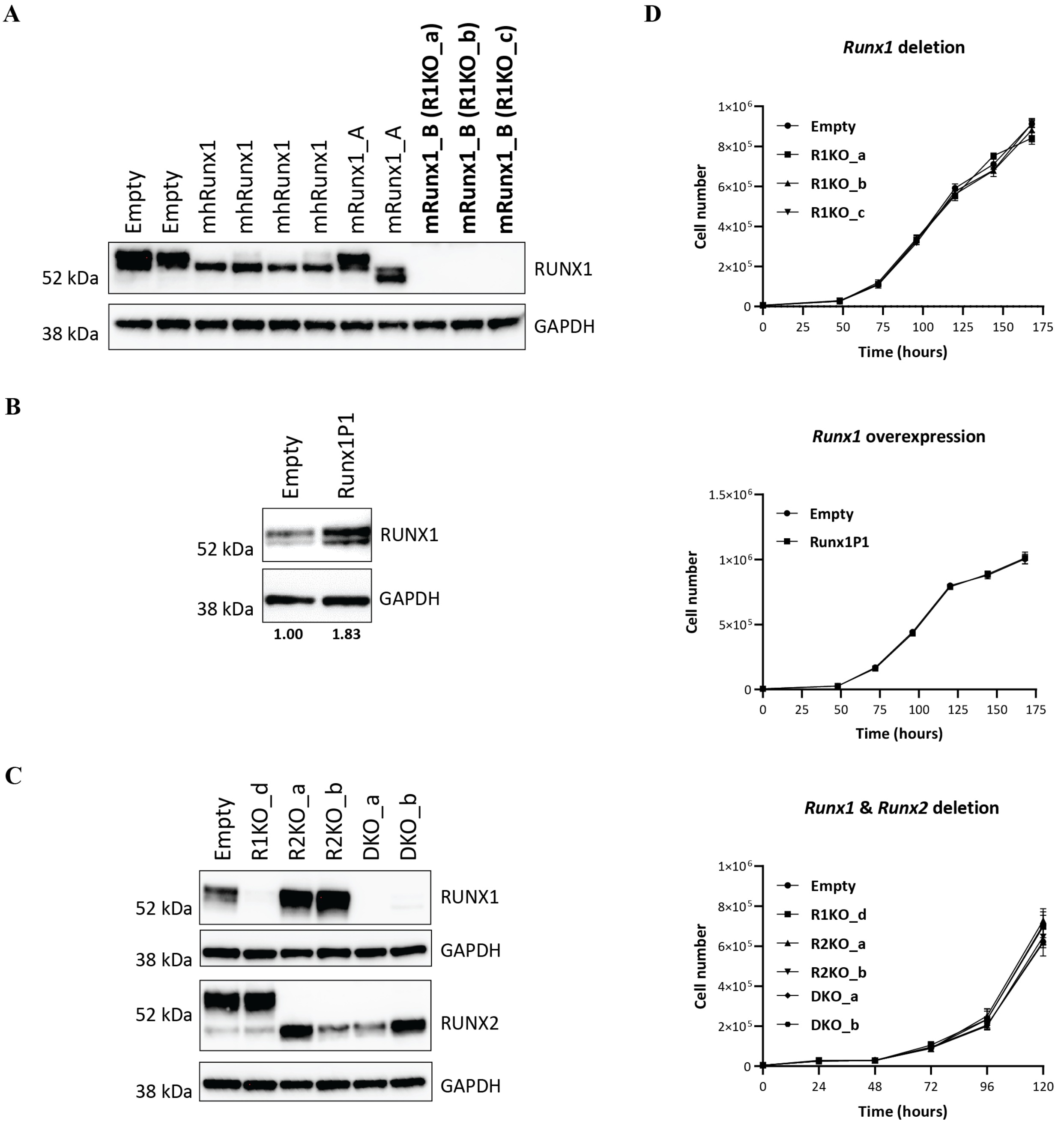

Supplementary Figure 5. Altered RUNX1 expression does not affect the phenotype of HC11 cells in 2D cultures.

A. Western blot of HC11 cells with empty vector (Empty) or three different *Runx1*-targeting sequences (*mhRunx1*, *mRunx1*_A and *mRunx1*_B), with RUNX1 depletion achieved in three independent *mRunx1*_B clones (R1KO_a, R1KO_b, R1KO_c) used in 2D growth (Supplementary Figure 5D top panel) and mammosphere (Fig. 4A) assays. GAPDH used as loading control.

B. Western blot of HC11 cells transduced with *Runx1P1* or empty vector. Densitometry analysis revealed an almost 2-fold increase in RUNX1 protein levels in *RunxP1* cells (values below). GAPDH used as loading control. Relative to Fig. 4B and Supplementary Figure 5D (middle panel).

C. Western blots of HC11 cells CRISPR-deleted for *Runx1* (R1KO_d), *Runx2* (R2KO_a, R2KO_b) or *Runx1/Runx2* (DKO_a, DKO_b). Note the non-specificity of the lower band in the RUNX2 blot (on-target gene editing was confirmed by genomic DNA sequencing). GAPDH used as loading control. Relative to Fig. 4C and Supplementary Figure 5D (bottom panel).

D. 2D growth curves of three independent *Runx1*-deleted HC11 clones (R1KO_a, R1KO_b and R1KO_c; top panel), *Runx1*-overexpressing clone (Runx1P1; middle panel), and an independent *Runx1*-deleted clone (R1KO_d), two independent *Runx2*-deleted clones (R2KO_a, R2KO_b) and two independent *Runx1*/*Runx2*-deleted clones (DKO_a, DKO_b; bottom panel). For each experiment, HC11 clone(s) were compared to their relative empty vector control (Empty). Results show mean with error bars representing standard deviation and are representative experiments of $N = 2$-4 experimental repeats per condition.
