# Supplementary material for: Runx1 and Runx2 act in concert to suppress Wnt/β-catenin-driven mammary tumourigenesis
Source: Br J Cancer. 2026 May 7;135(4):532–45. doi: 10.1038/s41416-026-03439-5 (PMC13427844; doi:10.1038/s41416-026-03439-5)
Supplement: Supplementary file 2 — Supplementary Tables legend [file 41416_2026_3439_MOESM2_ESM.docx]

# **Riggio et al, *Runx1 and Runx2 act in concert to suppress Wnt/β-catenin-driven mammary tumorigenesis***

# **Legends for Supplementary Tables 1 and 2**

## **Supplementary Table 1.**

List of 88 significantly altered DEGs from 9-weeks old *B-cat+/R1KO* (*n* = 5) compared to *B-cat+/WT* (*n* = 4) females (shown in Figure 5). Each sample comprised of pooled *tdRFP*-positive MMECs isolated from multiple mice of the same genotype. The differential expression of a gene was considered significant if it had an absolute fold change >1.5 and an adjusted *P* < 0.05, calculated using Wald test with DESeq2 package. Genes in bold are shared with significantly altered DEGs in *B-cat+/DKO* (Supplementary Table 2) and non-bold genes (13 of) only in the *B-cat+/R1KO* group.

## **Supplementary Table 2.**

List of 1,312 significantly altered DEGs from 9-weeks old *B-cat+/DKO* (*n* = 5) compared to *B-cat+/WT* (*n* = 4) mice (shown in Figure 5). Each sample comprised of pooled *tdRFP*-positive MMECs isolated from multiple mice of the same genotype. The differential expression of a gene was considered significant if it had an absolute fold change >1.5 and an adjusted *P* < 0.05, calculated using Wald test with DESeq2 package. Genes in bold are shared with significantly altered DEGs in *B-cat+/R1KO* (Supplementary Table 1) and non-bold genes only in the *B-cat+/DKO* group.
