# Supplementary material for: Runx1 and Runx2 act in concert to suppress Wnt/β-catenin-driven mammary tumourigenesis
Source: Br J Cancer. 2026 May 7;135(4):532–45. doi: 10.1038/s41416-026-03439-5 (PMC13427844; doi:10.1038/s41416-026-03439-5)
Supplement: Supplementary file 3 — Supplementary Table 1 [file 41416_2026_3439_MOESM3_ESM.pdf]

| Significant Genes | | | |
|---|---|---|---|
| Runx1 Knock-out | | | |
| Gene Name | Description | log2 FC | Adj p-value |
| **H19** | **H19, imprinted maternally expressed transcript [Source:MGI Symbol;Acc:MGI:95891]** | **2.641** | **3.808658e-06** |
| **Dkk2** | **dickkopf WNT signaling pathway inhibitor 2 [Source:MGI Symbol;Acc:MGI:1890663]** | **2.585** | **5.202147e-10** |
| **Hdc** | **histidine decarboxylase [Source:MGI Symbol;Acc:MGI:96062]** | **1.529** | **9.359363e-04** |
| **Bcl11b** | **B cell leukemia/lymphoma 11B [Source:MGI Symbol;Acc:MGI:1929913]** | **1.510** | **6.253528e-07** |
| **Tgfbi** | **transforming growth factor, beta induced [Source:MGI Symbol;Acc:MGI:99959]** | **1.246** | **1.833708e-04** |
| **6530402F18Rik** | **RIKEN cDNA 6530402F18 gene [Source:MGI Symbol;Acc:MGI:1923470]** | **1.169** | **6.186537e-04** |
| **Col7a1** | **collagen, type VII, alpha 1 [Source:MGI Symbol;Acc:MGI:88462]** | **1.154** | **9.359363e-04** |
| **Flrt1** | **fibronectin leucine rich transmembrane protein 1 [Source:MGI Symbol;Acc:MGI:3026647]** | **1.115** | **1.323395e-03** |
| **Il24** | **interleukin 24 [Source:MGI Symbol;Acc:MGI:2135548]** | **1.102** | **5.435477e-04** |
| **Chl1** | **cell adhesion molecule L1-like [Source:MGI Symbol;Acc:MGI:1098266]** | **1.077** | **3.321100e-05** |
| **P4ha1** | **procollagen-proline, 2-oxoglutarate 4-dioxygenase (proline 4-hydroxylase), alpha 1 polypeptide [Source:MGI Symbol;Acc:MGI:97463]** | **1.051** | **3.481518e-04** |
| **Slc27a3** | **solute carrier family 27 (fatty acid transporter), member 3 [Source:MGI** | **1.029** | **4.172441e-03** |

| Significant Genes<br>Runx1 Knock-out | | | |
|---|---|---|---|
| Gene Name | Description | log2 FC | Adj p-value |
| | **Symbol;Acc:MGI:1347358]** | | |
| **Pappa** | **pregnancy-associated plasma protein A [Source:MGI Symbol;Acc:MGI:97479]** | **0.984** | **4.030117e-03** |
| **Lrch2** | **leucine-rich repeats and calponin homology (CH) domain containing 2 [Source:MGI Symbol;Acc:MGI:2147870]** | **0.977** | **3.198256e-03** |
| **Ptprv** | **protein tyrosine phosphatase, receptor type, V [Source:MGI Symbol;Acc:MGI:108027]** | **0.974** | **5.377529e-03** |
| **Meis1** | **Meis homeobox 1 [Source:MGI Symbol;Acc:MGI:104717]** | **0.960** | **1.209811e-03** |
| **Tspan18** | **tetraspanin 18 [Source:MGI Symbol;Acc:MGI:1917186]** | **0.952** | **4.491123e-03** |
| **Sh2d5** | **SH2 domain containing 5 [Source:MGI Symbol;Acc:MGI:2446215]** | **0.948** | **2.883295e-03** |
| Meis2 | Meis homeobox 2 [Source:MGI Symbol;Acc:MGI:108564] | 0.942 | 4.491123e-03 |
| **Fn1** | **fibronectin 1 [Source:MGI Symbol;Acc:MGI:95566]** | **0.921** | **6.964084e-03** |
| **Pde4dip** | **phosphodiesterase 4D interacting protein (myomegalin) [Source:MGI Symbol;Acc:MGI:1891434]** | **0.871** | **5.706880e-08** |
| Acp6 | acid phosphatase 6, lysophosphatidic [Source:MGI Symbol;Acc:MGI:1931010] | 0.865 | 1.401768e-03 |
| Zfp949 | zinc finger protein 949 [Source:MGI Symbol;Acc:MGI:1918890] | 0.854 | 5.109096e-05 |
| **Plch2** | **phospholipase C, eta 2 [Source:MGI Symbol;Acc:MGI:2443078]** | **0.851** | **1.883886e-03** |

| Significant Genes<br>Runx1 Knock-out | | | |
|---|---|---|---|
| Gene Name | Description | log2 FC | Adj p-value |
| **Egfl6** | **EGF-like-domain, multiple 6 [Source:MGI Symbol;Acc:MGI:1858599]** | **0.829** | **1.308268e-02** |
| **Prdm6** | **PR domain containing 6 [Source:MGI Symbol;Acc:MGI:2684938]** | **0.822** | **1.389834e-02** |
| **Axl** | **AXL receptor tyrosine kinase [Source:MGI Symbol;Acc:MGI:1347244]** | **0.821** | **4.691642e-03** |
| Stc1 | stanniocalcin 1 [Source:MGI Symbol;Acc:MGI:109131] | 0.818 | 1.389834e-02 |
| **Fas** | **Fas (TNF receptor superfamily member 6) [Source:MGI Symbol;Acc:MGI:95484]** | **0.814** | **1.883886e-03** |
| **Porcn** | **porcupine O-acyltransferase [Source:MGI Symbol;Acc:MGI:1890212]** | **0.803** | **4.030117e-03** |
| **Tpm2** | **tropomyosin 2, beta [Source:MGI Symbol;Acc:MGI:98810]** | **0.737** | **1.939623e-02** |
| Abhd12b | abhydrolase domain containing 12B [Source:MGI Symbol;Acc:MGI:2685650] | 0.733 | 2.137925e-02 |
| **Snai2** | **snail family zinc finger 2 [Source:MGI Symbol;Acc:MGI:1096393]** | **0.724** | **2.248119e-02** |
| **Has2** | **hyaluronan synthase 2 [Source:MGI Symbol;Acc:MGI:107821]** | **0.700** | **2.396533e-02** |
| **Krt42** | **keratin 42 [Source:MGI Symbol;Acc:MGI:1915489]** | **0.681** | **2.264116e-02** |
| **Tns4** | **tensin 4 [Source:MGI Symbol;Acc:MGI:2144377]** | **0.677** | **2.647191e-02** |
| **Mapk4** | **mitogen-activated protein kinase 4 [Source:MGI Symbol;Acc:MGI:2444559]** | **0.668** | **2.685957e-02** |

| Significant Genes<br>Runx1 Knock-out | | | |
|---|---|---|---|
| Gene Name | Description | log2 FC | Adj p-value |
| **Phf24** | **PHD finger protein 24 [Source:MGI Symbol;Acc:MGI:2140712]** | **0.668** | **2.367643e-02** |
| **Large2** | **LARGE xylosyl- and glucuronyltransferase 2 [Source:MGI Symbol;Acc:MGI:2443769]** | **0.667** | **2.647191e-02** |
| **Nod2** | **nucleotide-binding oligomerization domain containing 2 [Source:MGI Symbol;Acc:MGI:2429397]** | **0.660** | **2.647191e-02** |
| **Slc4a11** | **solute carrier family 4, sodium bicarbonate transporter-like, member 11 [Source:MGI Symbol;Acc:MGI:2138987]** | **0.658** | **8.649263e-03** |
| **Serpinb10** | **serine (or cysteine) peptidase inhibitor, clade B (ovalbumin), member 10 [Source:MGI Symbol;Acc:MGI:2138648]** | **0.651** | **2.647191e-02** |
| **P2ry1** | **purinergic receptor P2Y, G-protein coupled 1 [Source:MGI Symbol;Acc:MGI:105049]** | **0.648** | **2.744058e-02** |
| **Adamts17** | **a disintegrin-like and metallopeptidase (reprolysin type) with thrombospondin type 1 motif, 17 [Source:MGI Symbol;Acc:MGI:3588195]** | **0.644** | **2.647191e-02** |
| **Lacc1** | **laccase domain containing 1 [Source:MGI Symbol;Acc:MGI:2445077]** | **0.639** | **2.402331e-02** |
| **Car12** | **carbonic anhydrase 12 [Source:MGI Symbol;Acc:MGI:1923709]** | **0.623** | **3.163225e-02** |
| **Krt10** | **keratin 10 [Source:MGI Symbol;Acc:MGI:96685]** | **0.611** | **1.781192e-02** |
| Nrp1 | neuropilin 1 [Source:MGI Symbol;Acc:MGI:106206] | 0.610 | 1.770225e-02 |

## Significant Genes

Runx1 Knock-out

| Gene Name | Description | log2 FC | Adj p-value |
|---|---|---|---|
| **Antxr1** | **anthrax toxin receptor 1 [Source:MGI Symbol;Acc:MGI:1916788]** | **0.609** | **3.310804e-02** |
| Stx1a | syntaxin 1A (brain) [Source:MGI Symbol;Acc:MGI:109355] | 0.593 | 2.647191e-02 |
| **Cntfr** | **ciliary neurotrophic factor receptor [Source:MGI Symbol;Acc:MGI:99605]** | **0.591** | **3.245599e-02** |
| **Ifitm3** | **interferon induced transmembrane protein 3 [Source:MGI Symbol;Acc:MGI:1913391]** | **0.590** | **3.502769e-02** |
| **Gata2** | **GATA binding protein 2 [Source:MGI Symbol;Acc:MGI:95662]** | **0.590** | **3.927009e-02** |
| **Tfcp2l1** | **transcription factor CP2-like 1 [Source:MGI Symbol;Acc:MGI:2444691]** | **−0.594** | **6.964084e-03** |
| **Asprv1** | **aspartic peptidase, retroviral-like 1 [Source:MGI Symbol;Acc:MGI:1915105]** | **−0.599** | **3.626540e-02** |
| Capn12 | calpain 12 [Source:MGI Symbol;Acc:MGI:1891369] | −0.605 | 3.642940e-02 |
| Timp3 | tissue inhibitor of metalloproteinase 3 [Source:MGI Symbol;Acc:MGI:98754] | −0.616 | 1.939623e-02 |
| **Cobl** | **cordon-bleu WH2 repeat [Source:MGI Symbol;Acc:MGI:105056]** | **−0.651** | **1.837800e-02** |
| Slc15a2 | solute carrier family 15 (H+/peptide transporter), member 2 [Source:MGI Symbol;Acc:MGI:1890457] | −0.661 | 2.647191e-02 |
| **Mme** | **membrane metallo endopeptidase [Source:MGI Symbol;Acc:MGI:97004]** | **−0.668** | **2.647191e-02** |
| Fam214a | family with sequence similarity 214, member A [Source:MGI Symbol;Acc:MGI:2387648] | −0.673 | 6.964084e-03 |

| Significant Genes<br>Runx1 Knock-out | | | |
|---|---|---|---|
| Gene Name | Description | log2 FC | Adj p-value |
| Rdh10 | retinol dehydrogenase 10 (all-trans) [Source:MGI Symbol;Acc:MGI:1924238] | −0.675 | 1.467976e-02 |
| **Krt7** | **keratin 7 [Source:MGI Symbol;Acc:MGI:96704]** | **−0.706** | **2.367643e-02** |
| **Eml5** | **echinoderm microtubule associated protein like 5 [Source:MGI Symbol;Acc:MGI:2442513]** | **−0.713** | **4.677095e-03** |
| **Atp6v1b1** | **ATPase, H+ transporting, lysosomal V1 subunit B1 [Source:MGI Symbol;Acc:MGI:103285]** | **−0.734** | **1.942746e-02** |
| **Map2** | **microtubule-associated protein 2 [Source:MGI Symbol;Acc:MGI:97175]** | **−0.742** | **1.773867e-02** |
| **Nabp1** | **nucleic acid binding protein 1 [Source:MGI Symbol;Acc:MGI:1923258]** | **−0.763** | **1.891063e-02** |
| **Cbx7** | **chromobox 7 [Source:MGI Symbol;Acc:MGI:1196439]** | **−0.810** | **6.964084e-03** |
| **Rhpn2** | **rhophilin, Rho GTPase binding protein 2 [Source:MGI Symbol;Acc:MGI:1289234]** | **−0.816** | **1.308268e-02** |
| **Spns2** | **spinster homolog 2 [Source:MGI Symbol;Acc:MGI:2384936]** | **−0.820** | **1.323395e-03** |
| **Myo6** | **myosin VI [Source:MGI Symbol;Acc:MGI:104785]** | **−0.832** | **5.551618e-03** |
| **Barx2** | **BarH-like homeobox 2 [Source:MGI Symbol;Acc:MGI:109617]** | **−0.856** | **1.188108e-02** |
| **Itih5** | **inter-alpha (globulin) inhibitor H5 [Source:MGI Symbol;Acc:MGI:1925751]** | **−0.860** | **7.503140e-03** |
| **Prss8** | **protease, serine 8 (prostasin) [Source:MGI Symbol;Acc:MGI:1923810]** | **−0.875** | **5.551618e-03** |

## Significant Genes

Runx1 Knock-out

| Gene Name | Description | log2 FC | Adj p-value |
|---|---|---|---|
| **Tpcn1** | **two pore channel 1 [Source:MGI Symbol;Acc:MGI:2182472]** | **−0.881** | **6.836887e-06** |
| **Dsc2** | **desmocollin 2 [Source:MGI Symbol;Acc:MGI:103221]** | **−0.900** | **5.551618e-03** |
| **Add3** | **adducin 3 (gamma) [Source:MGI Symbol;Acc:MGI:1351615]** | **−0.906** | **6.776834e-05** |
| Sprr1a | small proline-rich protein 1A [Source:MGI Symbol;Acc:MGI:106660] | −0.916 | 6.964084e-03 |
| **Eps8l1** | **EPS8-like 1 [Source:MGI Symbol;Acc:MGI:1914675]** | **−0.918** | **3.481518e-04** |
| **Sorl1** | **sortilin-related receptor, LDLR class A repeats-containing [Source:MGI Symbol;Acc:MGI:1202296]** | **−0.957** | **6.071142e-04** |
| **Gm14137** | **predicted gene 14137 [Source:MGI Symbol;Acc:MGI:3651144]** | **−1.009** | **4.172441e-03** |
| **Mal** | **myelin and lymphocyte protein, T cell differentiation protein [Source:MGI Symbol;Acc:MGI:892970]** | **−1.041** | **5.228852e-03** |
| **Gprc5a** | **G protein-coupled receptor, family C, group 5, member A [Source:MGI Symbol;Acc:MGI:1891250]** | **−1.150** | **1.883886e-03** |
| **Cryab** | **crystallin, alpha B [Source:MGI Symbol;Acc:MGI:88516]** | **−1.217** | **1.883886e-03** |
| **Apobec3** | **apolipoprotein B mRNA editing enzyme, catalytic polypeptide 3 [Source:MGI Symbol;Acc:MGI:1933111]** | **−1.264** | **1.209811e-03** |
| **Krt80** | **keratin 80 [Source:MGI Symbol;Acc:MGI:1921377]** | **−1.434** | **3.110889e-04** |

## Significant Genes

Runx1 Knock-out

| Gene Name | Description | log2 FC | Adj p-value |
|---|---|---|---|
| **Rptn** | **repetin [Source:MGI Symbol;Acc:MGI:1099055]** | **−1.544** | **2.043832e-03** |
| **Cnfn** | **cornifelin [Source:MGI Symbol;Acc:MGI:1919633]** | **−2.677** | **2.475522e-04** |
