# Supplementary material for: Runx1 and Runx2 act in concert to suppress Wnt/β-catenin-driven mammary tumourigenesis
Source: Br J Cancer. 2026 May 7;135(4):532–45. doi: 10.1038/s41416-026-03439-5 (PMC13427844; doi:10.1038/s41416-026-03439-5)
Supplement: Supplementary file 4 — Supplementary Table 2 [file 41416_2026_3439_MOESM4_ESM.pdf]

Significant Genes

Runx1 and Runx2 Knock-out

Gene Name	Description	log2 FC	Adj p-value
Krt1	keratin 1 [Source:MGI Symbol;Acc:MGI:96698]	6.232	8.909268e-16
**Krt10**	**keratin 10 [Source:MGI Symbol;Acc:MGI:96685]**	**4.996**	**2.823323e-16**
Aard	alanine and arginine rich domain containing protein [Source:MGI Symbol;Acc:MGI:2181621]	4.632	1.299585e-11
Gm28119	predicted gene 28119 [Source:MGI Symbol;Acc:MGI:5578825]	3.840	1.155448e-10
**Hdc**	**histidine decarboxylase [Source:MGI Symbol;Acc:MGI:96062]**	**3.611**	**5.666180e-14**
Crlf1	cytokine receptor-like factor 1 [Source:MGI Symbol;Acc:MGI:1340030]	3.339	1.595426e-20
Defb6	defensin beta 6 [Source:MGI Symbol;Acc:MGI:2151044]	3.269	2.294213e-10
Krt84	keratin 84 [Source:MGI Symbol;Acc:MGI:96700]	3.083	1.300029e-13
Krtdap	keratinocyte differentiation associated protein [Source:MGI Symbol;Acc:MGI:1928282]	3.031	5.706178e-11
Slc38a4	solute carrier family 38, member 4 [Source:MGI Symbol;Acc:MGI:1916604]	2.988	1.735890e-05
**Pappa**	**pregnancy-associated plasma protein A [Source:MGI Symbol;Acc:MGI:97479]**	**2.983**	**7.112746e-20**
Calm4	calmodulin 4 [Source:MGI Symbol;Acc:MGI:1931464]	2.948	1.979296e-08
**Dkk2**	**dickkopf WNT signaling pathway inhibitor 2 [Source:MGI Symbol;Acc:MGI:1890663]**	**2.918**	**3.042162e-15**

Significant Genes

Runx1 and Runx2 Knock-out

Gene Name	Description	log2 FC	Adj p-value
**Bcl11b**	**B cell leukemia/lymphoma 11B [Source:MGI Symbol;Acc:MGI:1929913]**	**2.914**	**7.103497e-24**
**Fn1**	**fibronectin 1 [Source:MGI Symbol;Acc:MGI:95566]**	**2.857**	**8.909268e-16**
Gm47316	predicted gene, 47316 [Source:MGI Symbol;Acc:MGI:6096197]	2.837	2.433792e-08
**Krt42**	**keratin 42 [Source:MGI Symbol;Acc:MGI:1915489]**	**2.823**	**7.236687e-11**
Glns-ps1	glutamine synthetase pseudogene 1 [Source:MGI Symbol;Acc:MGI:95740]	2.688	6.456330e-08
Cstdc5	cystatin domain containing 5 [Source:MGI Symbol;Acc:MGI:3696883]	2.685	7.306330e-05
Rnase4	ribonuclease, RNase A family 4 [Source:MGI Symbol;Acc:MGI:1926217]	2.684	4.066095e-11
**Il24**	**interleukin 24 [Source:MGI Symbol;Acc:MGI:2135548]**	**2.638**	**1.145243e-19**
Gsdmc	gasdermin C [Source:MGI Symbol;Acc:MGI:1933176]	2.559	1.340711e-04
Sprr1b	small proline-rich protein 1B [Source:MGI Symbol;Acc:MGI:106659]	2.557	3.643877e-06
Egr4	early growth response 4 [Source:MGI Symbol;Acc:MGI:99252]	2.543	6.496261e-08
**Tgfbi**	**transforming growth factor, beta induced [Source:MGI Symbol;Acc:MGI:99959]**	**2.499**	**2.842488e-15**
Aldh1a1	aldehyde dehydrogenase family 1, subfamily A1 [Source:MGI Symbol;Acc:MGI:1353450]	2.489	9.397669e-06

Significant Genes

Runx1 and Runx2 Knock-out

Gene Name	Description	log2 FC	Adj p-value
Fst	follistatin [Source:MGI Symbol;Acc:MGI:95586]	2.487	1.633103e-12
Trim12a	tripartite motif-containing 12A [Source:MGI Symbol;Acc:MGI:1923931]	2.453	3.084043e-04
Dpysl3	dihydropyrimidinase-like 3 [Source:MGI Symbol;Acc:MGI:1349762]	2.428	9.362484e-07
**H19**	**H19, imprinted maternally expressed transcript [Source:MGI Symbol;Acc:MGI:95891]**	**2.425**	**6.106591e-07**
Gas1	growth arrest specific 1 [Source:MGI Symbol;Acc:MGI:95655]	2.403	3.100578e-07
**Slc27a3**	**solute carrier family 27 (fatty acid transporter), member 3 [Source:MGI Symbol;Acc:MGI:1347358]**	**2.391**	**1.300785e-10**
Gm47024	predicted gene, 47024 [Source:MGI Symbol;Acc:MGI:6095713]	2.332	7.114657e-12
Zfp991	zinc finger protein 991 [Source:MGI Symbol;Acc:MGI:3701604]	2.322	2.414431e-06
Gm37949	predicted gene, 37949 [Source:MGI Symbol;Acc:MGI:5611177]	2.318	2.093829e-10
**Prdm6**	**PR domain containing 6 [Source:MGI Symbol;Acc:MGI:2684938]**	**2.301**	**7.388315e-09**
Gm15987	predicted gene 15987 [Source:MGI Symbol;Acc:MGI:3801849]	2.300	7.711873e-06
Gm38217	predicted gene, 38217 [Source:MGI Symbol;Acc:MGI:5611445]	2.294	5.792567e-08
**Lrch2**	**leucine-rich repeats and calponin homology (CH) domain containing 2**	**2.265**	**2.490415e-13**

Significant Genes

Runx1 and Runx2 Knock-out

Gene Name	Description	log2 FC	Adj p-value
	**[Source:MGI Symbol;Acc:MGI:2147870]**		
C1s1	complement component 1, s subcomponent 1 [Source:MGI Symbol;Acc:MGI:1355312]	2.241	4.205924e-05
Rgs16	regulator of G-protein signaling 16 [Source:MGI Symbol;Acc:MGI:108407]	2.234	3.357679e-07
Postn	periostin, osteoblast specific factor [Source:MGI Symbol;Acc:MGI:1926321]	2.205	1.567877e-07
Stom	stomatin [Source:MGI Symbol;Acc:MGI:95403]	2.190	2.812637e-08
Col4a1	collagen, type IV, alpha 1 [Source:MGI Symbol;Acc:MGI:88454]	2.186	2.846708e-06
Sfrp2	secreted frizzled-related protein 2 [Source:MGI Symbol;Acc:MGI:108078]	2.183	1.249935e-07
**Sh2d5**	**SH2 domain containing 5 [Source:MGI Symbol;Acc:MGI:2446215]**	**2.181**	**2.210652e-14**
**Egfl6**	**EGF-like-domain, multiple 6 [Source:MGI Symbol;Acc:MGI:1858599]**	**2.181**	**4.271002e-07**
Arc	activity regulated cytoskeletal-associated protein [Source:MGI Symbol;Acc:MGI:88067]	2.169	5.572180e-07
Lmo1	LIM domain only 1 [Source:MGI Symbol;Acc:MGI:102812]	2.166	6.713783e-06
**Col7a1**	**collagen, type VII, alpha 1 [Source:MGI Symbol;Acc:MGI:88462]**	**2.159**	**2.485769e-10**
**Ifitm3**	**interferon induced transmembrane protein 3 [Source:MGI Symbol;Acc:MGI:1913391]**	**2.143**	**5.792567e-08**
Gm47341	predicted gene, 47341 [Source:MGI Symbol;Acc:MGI:6096234]	2.115	1.637116e-08

Significant Genes

Runx1 and Runx2 Knock-out

Gene Name	Description	log2 FC	Adj p-value
Ugt1a5	UDP glucuronosyltransferase 1 family, polypeptide A5 [Source:MGI Symbol;Acc:MGI:3032634]	2.110	6.308302e-05
Lrrn1	leucine rich repeat protein 1, neuronal [Source:MGI Symbol;Acc:MGI:106038]	2.109	2.023463e-05
Gm14328	predicted gene 14328 [Source:MGI Symbol;Acc:MGI:3651951]	2.108	2.037953e-05
**Fas**	**Fas (TNF receptor superfamily member 6) [Source:MGI Symbol;Acc:MGI:95484]**	**2.107**	**7.110281e-23**
6820402A03Rik	RIKEN cDNA 6820402A03 gene [Source:MGI Symbol;Acc:MGI:1922992]	2.106	6.995960e-07
Sema3c	sema domain, immunoglobulin domain (Ig), short basic domain, secreted, (semaphorin) 3C [Source:MGI Symbol;Acc:MGI:107557]	2.089	8.240688e-19
Gm47026	predicted gene, 47026 [Source:MGI Symbol;Acc:MGI:6095717]	2.084	3.030163e-07
Nlrc5	NLR family, CARD domain containing 5 [Source:MGI Symbol;Acc:MGI:3612191]	2.070	6.737897e-06
**Chl1**	**cell adhesion molecule L1-like [Source:MGI Symbol;Acc:MGI:1098266]**	**2.069**	**2.425533e-18**
Mst1r	macrophage stimulating 1 receptor (c-met-related tyrosine kinase) [Source:MGI Symbol;Acc:MGI:99614]	2.065	1.134207e-09
Gm47342	predicted gene, 47342 [Source:MGI Symbol;Acc:MGI:6096236]	2.056	4.831539e-05
Clic6	chloride intracellular channel 6 [Source:MGI Symbol;Acc:MGI:2146607]	2.052	8.661525e-10

Significant Genes

Runx1 and Runx2 Knock-out

Gene Name	Description	log2 FC	Adj p-value
Aox4	aldehyde oxidase 4 [Source:MGI Symbol;Acc:MGI:1919122]	2.037	2.414431e-06
Ackr4	atypical chemokine receptor 4 [Source:MGI Symbol;Acc:MGI:2181676]	2.018	3.323028e-06
Crispld2	cysteine-rich secretory protein LCCL domain containing 2 [Source:MGI Symbol;Acc:MGI:1926142]	2.014	5.013183e-08
Artn	artemin [Source:MGI Symbol;Acc:MGI:1333791]	2.009	2.949033e-06
Prnp	prion protein [Source:MGI Symbol;Acc:MGI:97769]	2.006	8.547966e-05
**Tspan18**	**tetraspanin 18 [Source:MGI Symbol;Acc:MGI:1917186]**	**2.000**	**8.079353e-10**
Gm13055	predicted gene 13055 [Source:MGI Symbol;Acc:MGI:3649999]	1.999	4.273091e-04
Ptgs1	prostaglandin-endoperoxide synthase 1 [Source:MGI Symbol;Acc:MGI:97797]	1.990	2.981328e-06
H2-Q7	histocompatibility 2, Q region locus 7 [Source:MGI Symbol;Acc:MGI:95936]	1.988	2.393177e-04
Gm15726	predicted gene 15726 [Source:MGI Symbol;Acc:MGI:3783169]	1.982	5.851791e-04
Col17a1	collagen, type XVII, alpha 1 [Source:MGI Symbol;Acc:MGI:88450]	1.963	1.593545e-06
**Cntfr**	**ciliary neurotrophic factor receptor [Source:MGI Symbol;Acc:MGI:99605]**	**1.962**	**3.416541e-06**
Krt15	keratin 15 [Source:MGI Symbol;Acc:MGI:96689]	1.946	7.352358e-05

Significant Genes

Runx1 and Runx2 Knock-out

Gene Name	Description	log2 FC	Adj p-value
Rcn1	reticulocalbin 1 [Source:MGI Symbol;Acc:MGI:104559]	1.926	9.756002e-16
Fgfbp1	fibroblast growth factor binding protein 1 [Source:MGI Symbol;Acc:MGI:1096350]	1.913	4.700661e-04
Gm4949	predicted gene 4949 [Source:MGI Symbol;Acc:MGI:3646890]	1.913	8.287774e-06
Cxcl10	chemokine (C-X-C motif) ligand 10 [Source:MGI Symbol;Acc:MGI:1352450]	1.912	4.755280e-05
Pthlh	parathyroid hormone-like peptide [Source:MGI Symbol;Acc:MGI:97800]	1.908	2.067919e-10
Glul	glutamate-ammonia ligase (glutamine synthetase) [Source:MGI Symbol;Acc:MGI:95739]	1.907	2.541358e-05
Nrg1	neuregulin 1 [Source:MGI Symbol;Acc:MGI:96083]	1.902	7.606700e-07
Peli2	pellino 2 [Source:MGI Symbol;Acc:MGI:1891445]	1.901	4.483581e-11
2310046K23Rik	RIKEN cDNA 2310046K23 gene [Source:MGI Symbol;Acc:MGI:1924218]	1.899	1.485511e-03
Sox7	SRY (sex determining region Y)-box 7 [Source:MGI Symbol;Acc:MGI:98369]	1.898	1.961956e-05
Sparc	secreted acidic cysteine rich glycoprotein [Source:MGI Symbol;Acc:MGI:98373]	1.887	2.746186e-06
Col4a2	collagen, type IV, alpha 2 [Source:MGI Symbol;Acc:MGI:88455]	1.866	6.238842e-06
Dsc1	desmocollin 1 [Source:MGI Symbol;Acc:MGI:109173]	1.862	1.222881e-04

Significant Genes

Runx1 and Runx2 Knock-out

Gene Name	Description	log2 FC	Adj p-value
Fabp5	fatty acid binding protein 5, epidermal [Source:MGI Symbol;Acc:MGI:101790]	1.859	2.864644e-04
Serpinb3b	serine (or cysteine) peptidase inhibitor, clade B (ovalbumin), member 3B [Source:MGI Symbol;Acc:MGI:2683293]	1.857	2.785737e-03
Gm44441	predicted gene, 44441 [Source:MGI Symbol;Acc:MGI:5690833]	1.853	2.799071e-04
Pxdc1	PX domain containing 1 [Source:MGI Symbol;Acc:MGI:1914145]	1.844	2.882604e-06
**Nod2**	**nucleotide-binding oligomerization domain containing 2 [Source:MGI Symbol;Acc:MGI:2429397]**	**1.833**	**8.578110e-11**
**Porcn**	**porcupine O-acyltransferase [Source:MGI Symbol;Acc:MGI:1890212]**	**1.828**	**3.418081e-15**
Nsmce1	NSE1 homolog, SMC5-SMC6 complex component [Source:MGI Symbol;Acc:MGI:1914961]	1.822	3.897570e-07
Krt14	keratin 14 [Source:MGI Symbol;Acc:MGI:96688]	1.817	4.908340e-05
Pou3f1	POU domain, class 3, transcription factor 1 [Source:MGI Symbol;Acc:MGI:101896]	1.814	8.363408e-05
Spock2	sparc/osteonectin, cwcv and kazal-like domains proteoglycan 2 [Source:MGI Symbol;Acc:MGI:1891351]	1.814	1.459178e-05
**Serpinb10**	**serine (or cysteine) peptidase inhibitor, clade B (ovalbumin), member 10 [Source:MGI Symbol;Acc:MGI:2138648]**	**1.802**	**2.656280e-05**

Significant Genes

Runx1 and Runx2 Knock-out

Gene Name	Description	log2 FC	Adj p-value
Bdnf	brain derived neurotrophic factor [Source:MGI Symbol;Acc:MGI:88145]	1.798	4.144468e-08
**Snai2**	**snail family zinc finger 2 [Source:MGI Symbol;Acc:MGI:1096393]**	**1.793**	**1.663489e-06**
Gm37783	predicted gene, 37783 [Source:MGI Symbol;Acc:MGI:5611011]	1.793	4.253557e-07
Pdpn	podoplanin [Source:MGI Symbol;Acc:MGI:103098]	1.780	1.136991e-07
Gm37674	predicted gene, 37674 [Source:MGI Symbol;Acc:MGI:5610902]	1.765	6.198052e-06
**P4ha1**	**procollagen-proline, 2-oxoglutarate 4-dioxygenase (proline 4-hydroxylase), alpha 1 polypeptide [Source:MGI Symbol;Acc:MGI:97463]**	**1.759**	**1.464343e-10**
Mfsd2a	major facilitator superfamily domain containing 2A [Source:MGI Symbol;Acc:MGI:1923824]	1.758	5.413171e-06
Maf	avian musculoaponeurotic fibrosarcoma oncogene homolog [Source:MGI Symbol;Acc:MGI:96909]	1.757	7.955548e-06
Gm26626	predicted gene, 26626 [Source:MGI Symbol;Acc:MGI:5477120]	1.756	6.236086e-07
Fam83b	family with sequence similarity 83, member B [Source:MGI Symbol;Acc:MGI:2685362]	1.755	5.706178e-11
Art4	ADP-ribosyltransferase 4 [Source:MGI Symbol;Acc:MGI:1202710]	1.738	8.016090e-07
Arhgdib	Rho, GDP dissociation inhibitor (GDI) beta [Source:MGI Symbol;Acc:MGI:101940]	1.729	1.739611e-05

Significant Genes

Runx1 and Runx2 Knock-out

Gene Name	Description	log2 FC	Adj p-value
Gm45774	predicted gene 45774 [Source:MGI Symbol;Acc:MGI:5804889]	1.726	1.272683e-05
Trim32	tripartite motif-containing 32 [Source:MGI Symbol;Acc:MGI:1917057]	1.718	1.379670e-06
Stac	src homology three (SH3) and cysteine rich domain [Source:MGI Symbol;Acc:MGI:1201400]	1.718	1.889512e-06
**Car12**	**carbonic anhydrase 12 [Source:MGI Symbol;Acc:MGI:1923709]**	**1.715**	**3.636920e-09**
Gm48611	predicted gene, 48611 [Source:MGI Symbol;Acc:MGI:6098198]	1.712	1.903521e-03
Plod2	procollagen lysine, 2-oxoglutarate 5-dioxygenase 2 [Source:MGI Symbol;Acc:MGI:1347007]	1.706	1.486118e-16
Serpine1	serine (or cysteine) peptidase inhibitor, clade E, member 1 [Source:MGI Symbol;Acc:MGI:97608]	1.705	1.296518e-11
Gm15684	predicted gene 15684 [Source:MGI Symbol;Acc:MGI:3783126]	1.689	1.485511e-03
Klk7	kallikrein related-peptidase 7 (chymotryptic, stratum corneum) [Source:MGI Symbol;Acc:MGI:1346336]	1.682	4.358439e-04
Mt2	metallothionein 2 [Source:MGI Symbol;Acc:MGI:97172]	1.682	1.665751e-05
Ptges	prostaglandin E synthase [Source:MGI Symbol;Acc:MGI:1927593]	1.674	4.513753e-06
Hif1a	hypoxia inducible factor 1, alpha subunit [Source:MGI Symbol;Acc:MGI:106918]	1.674	9.390584e-06

Significant Genes<br>Runx1 and Runx2 Knock-out			
Gene Name	Description	log2 FC	Adj p-value
Plxna2	plexin A2 [Source:MGI Symbol;Acc:MGI:107684]	1.673	7.112746e-20
Serpinb11	serine (or cysteine) peptidase inhibitor, clade B (ovalbumin), member 11 [Source:MGI Symbol;Acc:MGI:1914207]	1.668	7.670593e-04
Glo1-ps	glyoxalase 1, pseudogene [Source:MGI Symbol;Acc:MGI:3649615]	1.663	1.196691e-04
Gm26788	predicted gene, 26788 [Source:MGI Symbol;Acc:MGI:5477282]	1.657	1.909120e-12
Tns3	tensin 3 [Source:MGI Symbol;Acc:MGI:2443012]	1.656	2.564516e-08
Il19	interleukin 19 [Source:MGI Symbol;Acc:MGI:1890472]	1.656	2.847963e-03
Aqp9	aquaporin 9 [Source:MGI Symbol;Acc:MGI:1891066]	1.647	7.868093e-06
S1pr3	sphingosine-1-phosphate receptor 3 [Source:MGI Symbol;Acc:MGI:1339365]	1.640	1.782209e-04
Dact1	dishevelled-binding antagonist of beta-catenin 1 [Source:MGI Symbol;Acc:MGI:1891740]	1.638	9.188008e-05
Tagln3	transgelin 3 [Source:MGI Symbol;Acc:MGI:1926784]	1.634	3.620793e-06
Fscn1	fascin actin-bundling protein 1 [Source:MGI Symbol;Acc:MGI:1352745]	1.630	3.437216e-04
Prss12	protease, serine 12 neurotrypsin (motopsin) [Source:MGI Symbol;Acc:MGI:1100881]	1.628	1.430048e-04
**Mapk4**	**mitogen-activated protein kinase 4 [Source:MGI Symbol;Acc:MGI:2444559]**	**1.626**	**5.042888e-06**

Significant Genes

Runx1 and Runx2 Knock-out

Gene Name	Description	log2 FC	Adj p-value
Gm31727	predicted gene, 31727 [Source:MGI Symbol;Acc:MGI:5590886]	1.623	3.994175e-03
Tifa	TRAF-interacting protein with forkhead-associated domain [Source:MGI Symbol;Acc:MGI:2182965]	1.619	1.557112e-04
**P2ry1**	**purinergic receptor P2Y, G-protein coupled 1 [Source:MGI Symbol;Acc:MGI:105049]**	**1.614**	**4.710832e-08**
Sting1	stimulator of interferon response cGAMP interactor 1 [Source:MGI Symbol;Acc:MGI:1919762]	1.614	3.888090e-10
9530013L04Rik	RIKEN cDNA 9530013L04 gene [Source:MGI Symbol;Acc:MGI:1924658]	1.599	3.286760e-03
**Tns4**	**tensin 4 [Source:MGI Symbol;Acc:MGI:2144377]**	**1.595**	**3.341269e-05**
**Plch2**	**phospholipase C, eta 2 [Source:MGI Symbol;Acc:MGI:2443078]**	**1.594**	**3.023420e-11**
H2-Q6	histocompatibility 2, Q region locus 6 [Source:MGI Symbol;Acc:MGI:95935]	1.590	5.104165e-03
Fam241a	family with sequence similarity 241, member A [Source:MGI Symbol;Acc:MGI:1917867]	1.590	7.216480e-08
Gm6166	predicted gene 6166 [Source:MGI Symbol;Acc:MGI:3645893]	1.588	4.639052e-03
Ahr	aryl-hydrocarbon receptor [Source:MGI Symbol;Acc:MGI:105043]	1.587	1.110826e-08
Apobec1	apolipoprotein B mRNA editing enzyme, catalytic polypeptide 1 [Source:MGI Symbol;Acc:MGI:103298]	1.582	1.035862e-06

Significant Genes

Runx1 and Runx2 Knock-out

Gene Name	Description	log2 FC	Adj p-value
Krt79	keratin 79 [Source:MGI Symbol;Acc:MGI:2385030]	1.574	1.164293e-04
Emb	embigin [Source:MGI Symbol;Acc:MGI:95321]	1.571	1.762588e-04
Gm18212	predicted gene, 18212 [Source:MGI Symbol;Acc:MGI:5010397]	1.566	1.973321e-03
Stfa3	stefin A3 [Source:MGI Symbol;Acc:MGI:106196]	1.560	7.629189e-03
Krt17	keratin 17 [Source:MGI Symbol;Acc:MGI:96691]	1.555	3.518870e-03
Rny1	RNA, Y1 small cytoplasmic, Ro-associated [Source:MGI Symbol;Acc:MGI:97995]	1.554	8.229272e-03
Ctsc	cathepsin C [Source:MGI Symbol;Acc:MGI:109553]	1.544	2.703721e-04
Gm43654	predicted gene 43654 [Source:MGI Symbol;Acc:MGI:5663791]	1.542	3.977409e-06
Smim3	small integral membrane protein 3 [Source:MGI Symbol;Acc:MGI:1917088]	1.541	4.424549e-08
Cadps	Ca2+-dependent secretion activator [Source:MGI Symbol;Acc:MGI:1350922]	1.539	2.835274e-04
**Tpm2**	**tropomyosin 2, beta [Source:MGI Symbol;Acc:MGI:98810]**	**1.533**	**1.392582e-06**
Gm38036	predicted gene, 38036 [Source:MGI Symbol;Acc:MGI:5611264]	1.531	4.932590e-06
Gm43803	predicted gene 43803 [Source:MGI Symbol;Acc:MGI:5663940]	1.529	6.898492e-03
Glis1	GLIS family zinc finger 1 [Source:MGI Symbol;Acc:MGI:2386723]	1.526	2.368000e-06

Significant Genes

Runx1 and Runx2 Knock-out

Gene Name	Description	log2 FC	Adj p-value
**Large2**	**LARGE xylosyl- and glucuronyltransferase 2 [Source:MGI Symbol;Acc:MGI:2443769]**	**1.525**	**4.175515e-07**
**Pde4dip**	**phosphodiesterase 4D interacting protein (myomegalin) [Source:MGI Symbol;Acc:MGI:1891434]**	**1.522**	**1.179543e-25**
Oaf	out at first homolog [Source:MGI Symbol;Acc:MGI:94852]	1.520	7.858228e-05
Runx1	runt related transcription factor 1 [Source:MGI Symbol;Acc:MGI:99852]	1.516	3.258348e-07
Pfkp	phosphofructokinase, platelet [Source:MGI Symbol;Acc:MGI:1891833]	1.516	4.402566e-08
Cbr3	carbonyl reductase 3 [Source:MGI Symbol;Acc:MGI:1309992]	1.516	5.203268e-04
Krt5	keratin 5 [Source:MGI Symbol;Acc:MGI:96702]	1.504	3.355827e-03
Myef2	myelin basic protein expression factor 2, repressor [Source:MGI Symbol;Acc:MGI:104592]	1.504	1.242863e-09
Rbms3	RNA binding motif, single stranded interacting protein [Source:MGI Symbol;Acc:MGI:2444477]	1.496	2.039745e-04
Gm28373	predicted gene 28373 [Source:MGI Symbol;Acc:MGI:5579079]	1.494	2.746845e-03
Gm47693	predicted gene, 47693 [Source:MGI Symbol;Acc:MGI:6096801]	1.493	2.829773e-04
Gm44416	predicted gene, 44416 [Source:MGI Symbol;Acc:MGI:5690808]	1.490	3.028306e-04
Mir100hg	Mir100 Mirlet7a-2 Mir125b-1 cluster host gene [Source:MGI Symbol;Acc:MGI:1920394]	1.488	5.564188e-03

Significant Genes

Runx1 and Runx2 Knock-out

Gene Name	Description	log2 FC	Adj p-value
Adamts9	a disintegrin-like and metallopeptidase (reprolysin type) with thrombospondin type 1 motif, 9 [Source:MGI Symbol;Acc:MGI:1916320]	1.486	3.567785e-04
Fam83f	family with sequence similarity 83, member F [Source:MGI Symbol;Acc:MGI:2146227]	1.483	1.761410e-05
Gm15283	predicted gene 15283 [Source:MGI Symbol;Acc:MGI:3705161]	1.479	7.846203e-04
Stac2	SH3 and cysteine rich domain 2 [Source:MGI Symbol;Acc:MGI:2144518]	1.477	7.568938e-10
Insyn1	inhibitory synaptic factor 1 [Source:MGI Symbol;Acc:MGI:2442108]	1.475	1.625993e-04
Mvb12b	multivesicular body subunit 12B [Source:MGI Symbol;Acc:MGI:1919793]	1.468	9.242312e-05
Elk3	ELK3, member of ETS oncogene family [Source:MGI Symbol;Acc:MGI:101762]	1.467	3.326061e-07
B4galt6	UDP-Gal:betaGlcNAc beta 1,4-galactosyltransferase, polypeptide 6 [Source:MGI Symbol;Acc:MGI:1928380]	1.464	1.595627e-08
Mpp2	membrane protein, palmitoylated 2 (MAGUK p55 subfamily member 2) [Source:MGI Symbol;Acc:MGI:1858257]	1.462	2.268868e-08
Ccdc80	coiled-coil domain containing 80 [Source:MGI Symbol;Acc:MGI:1915146]	1.457	1.352894e-03
Gm10564	predicted gene 10564 [Source:MGI Symbol;Acc:MGI:3704240]	1.454	2.486582e-03
Gm44414	predicted gene, 44414 [Source:MGI Symbol;Acc:MGI:5690806]	1.445	2.360468e-03

Significant Genes

Runx1 and Runx2 Knock-out

Gene Name	Description	log2 FC	Adj p-value
Gm43305	predicted gene 43305 [Source:MGI Symbol;Acc:MGI:5663442]	1.445	4.843596e-03
Abcc4	ATP-binding cassette, sub-family C (CFTR/MRP), member 4 [Source:MGI Symbol;Acc:MGI:2443111]	1.445	1.958169e-03
Prickle2	prickle planar cell polarity protein 2 [Source:MGI Symbol;Acc:MGI:1925144]	1.437	5.845930e-13
Gm44417	predicted gene, 44417 [Source:MGI Symbol;Acc:MGI:5690809]	1.436	2.012274e-03
**Flrt1**	**fibronectin leucine rich transmembrane protein 1 [Source:MGI Symbol;Acc:MGI:3026647]**	**1.427**	**7.726656e-05**
C130075A20Rik	RIKEN cDNA C130075A20 gene [Source:MGI Symbol;Acc:MGI:2443940]	1.425	9.056731e-04
**Antxr1**	**anthrax toxin receptor 1 [Source:MGI Symbol;Acc:MGI:1916788]**	**1.424**	**4.523522e-04**
Cpne8	copine VIII [Source:MGI Symbol;Acc:MGI:1914121]	1.423	2.933040e-05
Gm49307	predicted gene, 49307 [Source:MGI Symbol;Acc:MGI:6118808]	1.422	8.845645e-06
Phldb2	pleckstrin homology like domain, family B, member 2 [Source:MGI Symbol;Acc:MGI:2444981]	1.417	7.100561e-09
Elovl4	elongation of very long chain fatty acids (FEN1/Elo2, SUR4/Elo3, yeast)-like 4 [Source:MGI Symbol;Acc:MGI:1933331]	1.414	9.653624e-04
Spaca6	sperm acrosome associated 6 [Source:MGI Symbol;Acc:MGI:1922452]	1.408	2.761120e-13

Significant Genes

Runx1 and Runx2 Knock-out

Gene Name	Description	log2 FC	Adj p-value
Ltb4r2	leukotriene B4 receptor 2 [Source:MGI Symbol;Acc:MGI:1888501]	1.406	1.127956e-04
Car13	carbonic anhydrase 13 [Source:MGI Symbol;Acc:MGI:1931322]	1.402	1.917092e-06
Csgalnact1	chondroitin sulfate N-acetylgalactosaminyltransferase 1 [Source:MGI Symbol;Acc:MGI:2442354]	1.398	1.308330e-11
Nnat	neuronatin [Source:MGI Symbol;Acc:MGI:104716]	1.395	4.048594e-04
Arhgap24	Rho GTPase activating protein 24 [Source:MGI Symbol;Acc:MGI:1922647]	1.395	1.662436e-04
Nr4a3	nuclear receptor subfamily 4, group A, member 3 [Source:MGI Symbol;Acc:MGI:1352457]	1.392	3.117461e-05
Lama1	laminin, alpha 1 [Source:MGI Symbol;Acc:MGI:99892]	1.390	2.573018e-04
Dkk3	dickkopf WNT signaling pathway inhibitor 3 [Source:MGI Symbol;Acc:MGI:1354952]	1.390	3.857186e-04
Gm42898	predicted gene 42898 [Source:MGI Symbol;Acc:MGI:5663035]	1.387	5.309859e-03
Glo1	glyoxalase 1 [Source:MGI Symbol;Acc:MGI:95742]	1.384	2.992457e-11
Mex3b	mex3 RNA binding family member B [Source:MGI Symbol;Acc:MGI:1918252]	1.383	7.775241e-06
Psph	phosphoserine phosphatase [Source:MGI Symbol;Acc:MGI:97788]	1.380	1.272284e-05
Lgr6	leucine-rich repeat-containing G protein-coupled receptor 6 [Source:MGI	1.376	2.631300e-03

Significant Genes

Runx1 and Runx2 Knock-out

Gene Name	Description	log2 FC	Adj p-value
	Symbol;Acc:MGI:2441805]		
Dnah8	dynein, axonemal, heavy chain 8 [Source:MGI Symbol;Acc:MGI:107714]	1.375	8.806387e-03
Rnf217	ring finger protein 217 [Source:MGI Symbol;Acc:MGI:3610311]	1.375	7.447254e-05
Pcdh20	protocadherin 20 [Source:MGI Symbol;Acc:MGI:2443376]	1.374	4.634123e-03
Cavin1	caveolae associated 1 [Source:MGI Symbol;Acc:MGI:1277968]	1.370	1.239529e-04
Dzip1l	DAZ interacting protein 1-like [Source:MGI Symbol;Acc:MGI:1919757]	1.369	4.459171e-15
Dsg3	desmoglein 3 [Source:MGI Symbol;Acc:MGI:99499]	1.368	7.136341e-05
Vegfa	vascular endothelial growth factor A [Source:MGI Symbol;Acc:MGI:103178]	1.366	1.716672e-03
Gm44439	predicted gene, 44439 [Source:MGI Symbol;Acc:MGI:5690831]	1.365	6.940749e-03
**Has2**	**hyaluronan synthase 2 [Source:MGI Symbol;Acc:MGI:107821]**	**1.364**	**3.573497e-05**
Mycbp2	MYC binding protein 2, E3 ubiquitin protein ligase [Source:MGI Symbol;Acc:MGI:2179432]	1.362	2.650104e-07
Gm37018	predicted gene, 37018 [Source:MGI Symbol;Acc:MGI:5610246]	1.359	5.915820e-03
Chsy1	chondroitin sulfate synthase 1 [Source:MGI Symbol;Acc:MGI:2681120]	1.359	1.303555e-03
Arhgef4	Rho guanine nucleotide exchange factor (GEF) 4 [Source:MGI Symbol;Acc:MGI:2442507]	1.357	2.680497e-04

Significant Genes

Runx1 and Runx2 Knock-out

Gene Name	Description	log2 FC	Adj p-value
Gm9833	predicted gene 9833 [Source:MGI Symbol;Acc:MGI:3641855]	1.355	5.203268e-04
Ociad2	OCIA domain containing 2 [Source:MGI Symbol;Acc:MGI:1916377]	1.353	1.580885e-05
Gm7909	predicted gene 7909 [Source:MGI Symbol;Acc:MGI:3645643]	1.352	4.843596e-03
Gm45290	predicted gene 45290 [Source:MGI Symbol;Acc:MGI:5791126]	1.347	2.874211e-05
Ccl2	chemokine (C-C motif) ligand 2 [Source:MGI Symbol;Acc:MGI:98259]	1.347	1.037098e-02
Ugdh	UDP-glucose dehydrogenase [Source:MGI Symbol;Acc:MGI:1306785]	1.346	3.048788e-11
Epb41l3	erythrocyte membrane protein band 4.1 like 3 [Source:MGI Symbol;Acc:MGI:103008]	1.343	7.352358e-05
Grip1	glutamate receptor interacting protein 1 [Source:MGI Symbol;Acc:MGI:1921303]	1.341	3.342458e-05
Ppp1r12b	protein phosphatase 1, regulatory subunit 12B [Source:MGI Symbol;Acc:MGI:1916417]	1.337	6.131296e-09
Fam162a	family with sequence similarity 162, member A [Source:MGI Symbol;Acc:MGI:1917436]	1.334	7.176439e-04
Parp8	poly (ADP-ribose) polymerase family, member 8 [Source:MGI Symbol;Acc:MGI:1098713]	1.333	3.620793e-06
Lrp4	low density lipoprotein receptor-related protein 4 [Source:MGI Symbol;Acc:MGI:2442252]	1.333	4.662275e-03
Irf1	interferon regulatory factor 1 [Source:MGI Symbol;Acc:MGI:96590]	1.327	8.690126e-04

Significant Genes

Runx1 and Runx2 Knock-out

Gene Name	Description	log2 FC	Adj p-value
C130089K02Rik	RIKEN cDNA C130089K02 gene [Source:MGI Symbol;Acc:MGI:2444420]	1.326	2.931434e-03
St3gal2	ST3 beta-galactoside alpha-2,3-sialyltransferase 2 [Source:MGI Symbol;Acc:MGI:99427]	1.321	9.539359e-06
Gm45153	predicted gene 45153 [Source:MGI Symbol;Acc:MGI:5753729]	1.320	1.642269e-02
**Adamts17**	**a disintegrin-like and metallopeptidase (reprolysin type) with thrombospondin type 1 motif, 17 [Source:MGI Symbol;Acc:MGI:3588195]**	**1.317**	**4.039451e-06**
Pak6	p21 (RAC1) activated kinase 6 [Source:MGI Symbol;Acc:MGI:2679420]	1.316	8.580340e-07
9530086P17Rik	RIKEN cDNA 9530086P17 gene [Source:MGI Symbol;Acc:MGI:1925992]	1.312	1.926653e-04
Gm23722	predicted gene, 23722 [Source:MGI Symbol;Acc:MGI:5453499]	1.311	6.111508e-03
Nrep	neuronal regeneration related protein [Source:MGI Symbol;Acc:MGI:99444]	1.303	6.801444e-05
6430548M08Rik	RIKEN cDNA 6430548M08 gene [Source:MGI Symbol;Acc:MGI:2443793]	1.303	4.454310e-05
Slc66a2	solute carrier family 66 member 2 [Source:MGI Symbol;Acc:MGI:1914193]	1.294	1.062758e-03
Rnf150	ring finger protein 150 [Source:MGI Symbol;Acc:MGI:2443860]	1.290	1.930827e-05
Sbspon	somatomedin B and thrombospondin, type 1 domain containing [Source:MGI Symbol;Acc:MGI:2684952]	1.288	8.229272e-03

Significant Genes

Runx1 and Runx2 Knock-out

Gene Name	Description	log2 FC	Adj p-value
Ift57	intraflagellar transport 57 [Source:MGI Symbol;Acc:MGI:1921166]	1.288	3.854899e-06
Gm38387	predicted gene, 38387 [Source:MGI Symbol;Acc:MGI:5613622]	1.285	6.534189e-04
Gm47061	predicted gene, 47061 [Source:MGI Symbol;Acc:MGI:6095771]	1.284	3.285576e-03
Lrp8	low density lipoprotein receptor-related protein 8, apolipoprotein e receptor [Source:MGI Symbol;Acc:MGI:1340044]	1.281	3.136523e-06
Mt1	metallothionein 1 [Source:MGI Symbol;Acc:MGI:97171]	1.281	1.093215e-03
Degs1	delta(4)-desaturase, sphingolipid 1 [Source:MGI Symbol;Acc:MGI:1097711]	1.281	9.416611e-04
Gm42899	predicted gene 42899 [Source:MGI Symbol;Acc:MGI:5663036]	1.280	9.377422e-03
Cxcl9	chemokine (C-X-C motif) ligand 9 [Source:MGI Symbol;Acc:MGI:1352449]	1.278	2.058017e-02
Gm37943	predicted gene, 37943 [Source:MGI Symbol;Acc:MGI:5611171]	1.277	2.703721e-04
Dmkn	dermokine [Source:MGI Symbol;Acc:MGI:1920962]	1.276	4.798248e-03
Moxd1	monooxygenase, DBH-like 1 [Source:MGI Symbol;Acc:MGI:1921582]	1.276	5.250050e-03
Carmn	cardiac mesoderm enhancer-associated non-coding RNA [Source:MGI Symbol;Acc:MGI:4439832]	1.274	2.070716e-02
Gm37258	predicted gene, 37258 [Source:MGI Symbol;Acc:MGI:5610486]	1.272	4.077974e-03

Significant Genes

Runx1 and Runx2 Knock-out

Gene Name	Description	log2 FC	Adj p-value
**Slc4a11**	**solute carrier family 4, sodium bicarbonate transporter-like, member 11 [Source:MGI Symbol;Acc:MGI:2138987]**	**1.269**	**7.094925e-10**
Evl	Ena-vasodilator stimulated phosphoprotein [Source:MGI Symbol;Acc:MGI:1194884]	1.267	4.872736e-04
Cav1	caveolin 1, caveolae protein [Source:MGI Symbol;Acc:MGI:102709]	1.263	2.403103e-05
Lamc2	laminin, gamma 2 [Source:MGI Symbol;Acc:MGI:99913]	1.262	2.117183e-04
Gm43805	predicted gene 43805 [Source:MGI Symbol;Acc:MGI:5663942]	1.253	1.201675e-02
Mark1	MAP/microtubule affinity regulating kinase 1 [Source:MGI Symbol;Acc:MGI:2664902]	1.251	3.686789e-04
Pcdh7	protocadherin 7 [Source:MGI Symbol;Acc:MGI:1860487]	1.251	3.419844e-08
Irx4	Iroquois homeobox 4 [Source:MGI Symbol;Acc:MGI:1355275]	1.250	1.057688e-02
Gm43804	predicted gene 43804 [Source:MGI Symbol;Acc:MGI:5663941]	1.249	1.803658e-02
**Phf24**	**PHD finger protein 24 [Source:MGI Symbol;Acc:MGI:2140712]**	**1.247**	**8.675014e-06**
Hdgfl3	HDGF like 3 [Source:MGI Symbol;Acc:MGI:1352760]	1.246	2.072221e-05
Enah	ENAH actin regulator [Source:MGI Symbol;Acc:MGI:108360]	1.244	2.627177e-04
Gm16045	predicted gene 16045 [Source:MGI Symbol;Acc:MGI:3801990]	1.244	4.422373e-03

Significant Genes

Runx1 and Runx2 Knock-out

Gene Name	Description	log2 FC	Adj p-value
B2m	beta-2 microglobulin [Source:MGI Symbol;Acc:MGI:88127]	1.238	2.703661e-03
Nlgn2	neuroligin 2 [Source:MGI Symbol;Acc:MGI:2681835]	1.237	1.771686e-11
Gm15414	predicted gene 15414 [Source:MGI Symbol;Acc:MGI:3705307]	1.236	1.656728e-02
St6gal1	beta galactoside alpha 2,6 sialyltransferase 1 [Source:MGI Symbol;Acc:MGI:108470]	1.234	1.151768e-03
Gm47586	predicted gene, 47586 [Source:MGI Symbol;Acc:MGI:6096627]	1.233	6.958659e-03
Gm48602	predicted gene, 48602 [Source:MGI Symbol;Acc:MGI:6098182]	1.229	9.283855e-03
Akap11	A kinase (PRKA) anchor protein 11 [Source:MGI Symbol;Acc:MGI:2684060]	1.227	7.206924e-04
Egr2	early growth response 2 [Source:MGI Symbol;Acc:MGI:95296]	1.226	1.302879e-04
Gm29481	predicted gene 29481 [Source:MGI Symbol;Acc:MGI:5580187]	1.225	2.370599e-04
Camta1	calmodulin binding transcription activator 1 [Source:MGI Symbol;Acc:MGI:2140230]	1.216	9.447629e-27
Kansl1l	KAT8 regulatory NSL complex subunit 1-like [Source:MGI Symbol;Acc:MGI:1915941]	1.213	2.610621e-05
Efhd1	EF hand domain containing 1 [Source:MGI Symbol;Acc:MGI:1921607]	1.213	7.467866e-04
Gpr55	G protein-coupled receptor 55 [Source:MGI Symbol;Acc:MGI:2685064]	1.212	2.381554e-03

Significant Genes

Runx1 and Runx2 Knock-out

Gene Name	Description	log2 FC	Adj p-value
Ralgps2	Ral GEF with PH domain and SH3 binding motif 2 [Source:MGI Symbol;Acc:MGI:1925505]	1.208	2.665125e-05
Pcdh17	protocadherin 17 [Source:MGI Symbol;Acc:MGI:2684924]	1.204	8.334350e-03
Siglec15	sialic acid binding Ig-like lectin 15 [Source:MGI Symbol;Acc:MGI:3646642]	1.203	2.367142e-02
Gm49310	predicted gene, 49310 [Source:MGI Symbol;Acc:MGI:6118813]	1.199	3.309237e-03
Inka1	inka box actin regulator 1 [Source:MGI Symbol;Acc:MGI:1915426]	1.199	9.326750e-03
Sh3rf2	SH3 domain containing ring finger 2 [Source:MGI Symbol;Acc:MGI:2444628]	1.196	1.221538e-05
G530011O06Rik	RIKEN cDNA G530011O06 gene [Source:MGI Symbol;Acc:MGI:3603513]	1.196	2.051749e-02
Sostdc1	sclerostin domain containing 1 [Source:MGI Symbol;Acc:MGI:1913292]	1.185	3.235491e-03
Tbx3	T-box 3 [Source:MGI Symbol;Acc:MGI:98495]	1.184	1.902819e-03
Ap1ar	adaptor-related protein complex 1 associated regulatory protein [Source:MGI Symbol;Acc:MGI:2384822]	1.184	3.461791e-07
Fam83c	family with sequence similarity 83, member C [Source:MGI Symbol;Acc:MGI:1918655]	1.182	4.125159e-04
Gm43621	predicted gene 43621 [Source:MGI Symbol;Acc:MGI:5663758]	1.180	2.297039e-02
Fbln1	fibulin 1 [Source:MGI Symbol;Acc:MGI:95487]	1.179	1.656728e-02

Significant Genes

Runx1 and Runx2 Knock-out

Gene Name	Description	log2 FC	Adj p-value
Slc2a9	solute carrier family 2 (facilitated glucose transporter), member 9 [Source:MGI Symbol;Acc:MGI:2152844]	1.178	7.134593e-06
Cd109	CD109 antigen [Source:MGI Symbol;Acc:MGI:2445221]	1.172	8.033432e-06
Fabp5l2	fatty acid binding protein 5-like 2 [Source:MGI Symbol;Acc:MGI:3644282]	1.172	2.656300e-02
Oat	ornithine aminotransferase [Source:MGI Symbol;Acc:MGI:97394]	1.167	1.127956e-04
C130074G19Rik	RIKEN cDNA C130074G19 gene [Source:MGI Symbol;Acc:MGI:2444831]	1.165	8.806688e-04
Auts2	autism susceptibility candidate 2 [Source:MGI Symbol;Acc:MGI:1919847]	1.159	4.548171e-05
Avpi1	arginine vasopressin-induced 1 [Source:MGI Symbol;Acc:MGI:1916784]	1.159	9.234132e-03
Gm38001	predicted gene, 38001 [Source:MGI Symbol;Acc:MGI:5611229]	1.157	6.077997e-03
Gm42995	predicted gene 42995 [Source:MGI Symbol;Acc:MGI:5663132]	1.156	1.879884e-02
C79798	expressed sequence C79798 [Source:MGI Symbol;Acc:MGI:2139494]	1.155	6.333206e-03
Zfp385a	zinc finger protein 385A [Source:MGI Symbol;Acc:MGI:1352495]	1.150	2.861102e-03
Slco4a1	solute carrier organic anion transporter family, member 4a1 [Source:MGI Symbol;Acc:MGI:1351866]	1.149	7.094633e-05
Dcbld2	discoidin, CUB and LCCL domain containing 2 [Source:MGI Symbol;Acc:MGI:1920629]	1.144	7.727561e-10

Significant Genes

Runx1 and Runx2 Knock-out

Gene Name	Description	log2 FC	Adj p-value
Cxcr4	chemokine (C-X-C motif) receptor 4 [Source:MGI Symbol;Acc:MGI:109563]	1.142	6.262628e-05
Il4ra	interleukin 4 receptor, alpha [Source:MGI Symbol;Acc:MGI:105367]	1.139	1.310319e-02
Fndc4	fibronectin type III domain containing 4 [Source:MGI Symbol;Acc:MGI:1917195]	1.139	2.979583e-05
Rps6ka4	ribosomal protein S6 kinase, polypeptide 4 [Source:MGI Symbol;Acc:MGI:1930076]	1.136	2.512935e-03
Fto	fat mass and obesity associated [Source:MGI Symbol;Acc:MGI:1347093]	1.134	4.859127e-04
Gm47603	predicted gene, 47603 [Source:MGI Symbol;Acc:MGI:6096658]	1.134	2.288114e-02
Gm17334	predicted gene, 17334 [Source:MGI Symbol;Acc:MGI:4936968]	1.133	1.055862e-02
Sorcs2	sortilin-related VPS10 domain containing receptor 2 [Source:MGI Symbol;Acc:MGI:1932289]	1.131	1.417471e-04
Kank1	KN motif and ankyrin repeat domains 1 [Source:MGI Symbol;Acc:MGI:2147707]	1.127	1.839759e-03
Trim35	tripartite motif-containing 35 [Source:MGI Symbol;Acc:MGI:1914104]	1.126	6.422847e-04
Thbd	thrombomodulin [Source:MGI Symbol;Acc:MGI:98736]	1.121	1.963862e-02
Trim13	tripartite motif-containing 13 [Source:MGI Symbol;Acc:MGI:1913847]	1.119	4.597686e-03
Adora2b	adenosine A2b receptor [Source:MGI Symbol;Acc:MGI:99403]	1.118	3.911271e-03

Significant Genes

Runx1 and Runx2 Knock-out

Gene Name	Description	log2 FC	Adj p-value
Rell1	RELT-like 1 [Source:MGI Symbol;Acc:MGI:2140767]	1.118	4.142988e-03
Gm37747	predicted gene, 37747 [Source:MGI Symbol;Acc:MGI:5610975]	1.117	7.632337e-03
Matn2	matrilin 2 [Source:MGI Symbol;Acc:MGI:109613]	1.114	2.465741e-03
1700109K24Rik	RIKEN cDNA 1700109K24 gene [Source:MGI Symbol;Acc:MGI:1921553]	1.113	5.938939e-04
Appl1	adaptor protein, phosphotyrosine interaction, PH domain and leucine zipper containing 1 [Source:MGI Symbol;Acc:MGI:1920243]	1.112	6.589527e-05
Diaph2	diaphanous related formin 2 [Source:MGI Symbol;Acc:MGI:1858500]	1.111	1.155448e-10
Robo2	roundabout guidance receptor 2 [Source:MGI Symbol;Acc:MGI:1890110]	1.109	2.472283e-03
Rbp1	retinol binding protein 1, cellular [Source:MGI Symbol;Acc:MGI:97876]	1.104	2.469465e-02
Gas2l3	growth arrest-specific 2 like 3 [Source:MGI Symbol;Acc:MGI:1918780]	1.104	1.621464e-03
Gm37465	predicted gene, 37465 [Source:MGI Symbol;Acc:MGI:5610693]	1.103	1.495047e-02
L3mbtl3	L3MBTL3 histone methyl-lysine binding protein [Source:MGI Symbol;Acc:MGI:2143628]	1.103	6.926622e-09
Cdca7	cell division cycle associated 7 [Source:MGI Symbol;Acc:MGI:1914203]	1.097	4.387588e-06
Gm14052	predicted gene 14052 [Source:MGI Symbol;Acc:MGI:3650009]	1.097	1.206351e-02

Significant Genes<br>Runx1 and Runx2 Knock-out			
Gene Name	Description	log2 FC	Adj p-value
**Meis1**	**Meis homeobox 1 [Source:MGI Symbol;Acc:MGI:104717]**	**1.095**	**1.482615e-04**
Gm15819	predicted gene 15819 [Source:MGI Symbol;Acc:MGI:3802175]	1.094	1.969521e-02
Ctnnal1	catenin (cadherin associated protein), alpha-like 1 [Source:MGI Symbol;Acc:MGI:1859649]	1.093	1.308928e-04
Gm20008	predicted gene, 20008 [Source:MGI Symbol;Acc:MGI:5012193]	1.092	2.462325e-04
Atp1b3	ATPase, Na+/K+ transporting, beta 3 polypeptide [Source:MGI Symbol;Acc:MGI:107788]	1.089	4.464511e-03
Gm29480	predicted gene 29480 [Source:MGI Symbol;Acc:MGI:5580186]	1.088	3.844219e-03
Tfap2b	transcription factor AP-2 beta [Source:MGI Symbol;Acc:MGI:104672]	1.087	1.947963e-03
Slc14a1	solute carrier family 14 (urea transporter), member 1 [Source:MGI Symbol;Acc:MGI:1351654]	1.084	3.841725e-02
Gm43787	predicted gene 43787 [Source:MGI Symbol;Acc:MGI:5663924]	1.083	7.183699e-03
Gm43917	predicted gene, 43917 [Source:MGI Symbol;Acc:MGI:5690309]	1.083	2.146330e-03
Tsku	tsukushi, small leucine rich proteoglycan [Source:MGI Symbol;Acc:MGI:2443855]	1.081	2.717714e-04
Ccdc68	coiled-coil domain containing 68 [Source:MGI Symbol;Acc:MGI:3612676]	1.080	2.468963e-04
Etv4	ets variant 4 [Source:MGI Symbol;Acc:MGI:99423]	1.079	1.969521e-02

Significant Genes

Runx1 and Runx2 Knock-out

Gene Name	Description	log2 FC	Adj p-value
Prr7	proline rich 7 (synaptic) [Source:MGI Symbol;Acc:MGI:3487246]	1.071	2.660013e-02
Tnfaip8	tumor necrosis factor, alpha-induced protein 8 [Source:MGI Symbol;Acc:MGI:2147191]	1.071	2.566498e-06
Ak4	adenylate kinase 4 [Source:MGI Symbol;Acc:MGI:87979]	1.070	1.752567e-03
Lmo4	LIM domain only 4 [Source:MGI Symbol;Acc:MGI:109360]	1.067	2.045420e-03
Gm42996	predicted gene 42996 [Source:MGI Symbol;Acc:MGI:5663133]	1.067	1.561886e-02
Ahi1	Abelson helper integration site 1 [Source:MGI Symbol;Acc:MGI:87971]	1.066	5.611943e-09
Mir205hg	Mir205 host gene [Source:MGI Symbol;Acc:MGI:1918050]	1.064	4.639052e-03
Alpk1	alpha-kinase 1 [Source:MGI Symbol;Acc:MGI:1918731]	1.063	3.673128e-02
Lipg	lipase, endothelial [Source:MGI Symbol;Acc:MGI:1341803]	1.063	8.321805e-03
Kremen2	kringle containing transmembrane protein 2 [Source:MGI Symbol;Acc:MGI:1920266]	1.061	1.708249e-02
Gm48342	predicted gene, 48342 [Source:MGI Symbol;Acc:MGI:6097804]	1.060	1.973165e-02
Slc30a2	solute carrier family 30 (zinc transporter), member 2 [Source:MGI Symbol;Acc:MGI:106637]	1.056	1.089484e-03
Rgcc	regulator of cell cycle [Source:MGI Symbol;Acc:MGI:1913464]	1.054	1.918886e-03

Significant Genes

Runx1 and Runx2 Knock-out

Gene Name	Description	log2 FC	Adj p-value
B4galnt4	beta-1,4-N-acetyl-galactosaminyl transferase 4 [Source:MGI Symbol;Acc:MGI:2652891]	1.053	8.373218e-05
Lama3	laminin, alpha 3 [Source:MGI Symbol;Acc:MGI:99909]	1.053	1.250329e-02
Tshz2	teashirt zinc finger family member 2 [Source:MGI Symbol;Acc:MGI:2153084]	1.048	2.325964e-02
Slc26a2	solute carrier family 26 (sulfate transporter), member 2 [Source:MGI Symbol;Acc:MGI:892977]	1.048	8.074242e-06
**Ptprv**	**protein tyrosine phosphatase, receptor type, V [Source:MGI Symbol;Acc:MGI:108027]**	**1.046**	**7.141753e-03**
4831440D22Rik	RIKEN cDNA 4831440D22 gene [Source:MGI Symbol;Acc:MGI:2444771]	1.044	2.697171e-02
Begain	brain-enriched guanylate kinase-associated [Source:MGI Symbol;Acc:MGI:3044626]	1.044	1.634627e-03
Gm37537	predicted gene, 37537 [Source:MGI Symbol;Acc:MGI:5610765]	1.043	1.976966e-02
Igfbp4	insulin-like growth factor binding protein 4 [Source:MGI Symbol;Acc:MGI:96439]	1.040	6.940749e-03
Gm13394	predicted gene 13394 [Source:MGI Symbol;Acc:MGI:3651848]	1.031	3.072193e-02
Rragd	Ras-related GTP binding D [Source:MGI Symbol;Acc:MGI:1098604]	1.030	8.149079e-03
Atp8b2	ATPase, class I, type 8B, member 2 [Source:MGI Symbol;Acc:MGI:1859660]	1.026	1.238461e-11
Gm47602	predicted gene, 47602 [Source:MGI Symbol;Acc:MGI:6096656]	1.021	2.059055e-02

Significant Genes

Runx1 and Runx2 Knock-out

Gene Name	Description	log2 FC	Adj p-value
**Gata2**	**GATA binding protein 2 [Source:MGI Symbol;Acc:MGI:95662]**	**1.021**	**5.037953e-03**
Tnk1	tyrosine kinase, non-receptor, 1 [Source:MGI Symbol;Acc:MGI:1930958]	1.020	2.816501e-03
Hand2	heart and neural crest derivatives expressed 2 [Source:MGI Symbol;Acc:MGI:103580]	1.019	4.488111e-02
Eno1	enolase 1, alpha non-neuron [Source:MGI Symbol;Acc:MGI:95393]	1.019	6.388224e-03
Ak1	adenylate kinase 1 [Source:MGI Symbol;Acc:MGI:87977]	1.018	2.164748e-02
5430420F09Rik	RIKEN cDNA 5430420F09 gene [Source:MGI Symbol;Acc:MGI:1921741]	1.017	2.575340e-02
Tfap2a	transcription factor AP-2, alpha [Source:MGI Symbol;Acc:MGI:104671]	1.015	8.492290e-03
Igsf9	immunoglobulin superfamily, member 9 [Source:MGI Symbol;Acc:MGI:2135283]	1.014	4.015497e-04
Hyal1	hyaluronoglucosaminidase 1 [Source:MGI Symbol;Acc:MGI:96298]	1.014	5.172439e-03
Il23a	interleukin 23, alpha subunit p19 [Source:MGI Symbol;Acc:MGI:1932410]	1.013	2.057334e-02
Stox2	storkhead box 2 [Source:MGI Symbol;Acc:MGI:1918319]	1.012	2.595753e-04
Fbn2	fibrillin 2 [Source:MGI Symbol;Acc:MGI:95490]	1.012	1.086418e-03
Lnx2	ligand of numb-protein X 2 [Source:MGI Symbol;Acc:MGI:2155959]	1.009	6.112760e-04
Kirrel3	kirre like nephrin family adhesion molecule 3 [Source:MGI Symbol;Acc:MGI:1914953]	1.008	2.793357e-02

Significant Genes

Runx1 and Runx2 Knock-out

Gene Name	Description	log2 FC	Adj p-value
D730045A05Rik	RIKEN cDNA D730045A05 gene [Source:MGI Symbol;Acc:MGI:1925105]	1.007	1.105668e-02
Masp1	mannan-binding lectin serine peptidase 1 [Source:MGI Symbol;Acc:MGI:88492]	1.007	4.899793e-02
Trim24	tripartite motif-containing 24 [Source:MGI Symbol;Acc:MGI:109275]	1.006	7.216480e-08
Xrcc5	X-ray repair complementing defective repair in Chinese hamster cells 5 [Source:MGI Symbol;Acc:MGI:104517]	1.006	4.125159e-04
4930599N23Rik	RIKEN cDNA 4930599N23 gene [Source:MGI Symbol;Acc:MGI:1922629]	1.004	2.627759e-02
E330011O21Rik	RIKEN cDNA E330011O21 gene [Source:MGI Symbol;Acc:MGI:3759668]	1.003	2.822560e-03
Dsg1b	desmoglein 1 beta [Source:MGI Symbol;Acc:MGI:2664357]	1.000	2.250455e-02
Cbx2	chromobox 2 [Source:MGI Symbol;Acc:MGI:88289]	0.999	3.351098e-03
Arid5a	AT rich interactive domain 5A (MRF1-like) [Source:MGI Symbol;Acc:MGI:2443039]	0.999	5.595748e-03
Gm43186	predicted gene 43186 [Source:MGI Symbol;Acc:MGI:5663323]	0.997	2.072744e-02
Lgr4	leucine-rich repeat-containing G protein-coupled receptor 4 [Source:MGI Symbol;Acc:MGI:1891468]	0.995	1.950265e-02
Them5	thioesterase superfamily member 5 [Source:MGI Symbol;Acc:MGI:1913448]	0.993	3.526310e-02
Asap2	ArfGAP with SH3 domain, ankyrin repeat and PH domain 2 [Source:MGI	0.992	1.454843e-06

Significant Genes			
Runx1 and Runx2 Knock-out			
Gene Name	Description	log2 FC	Adj p-value
	Symbol;Acc:MGI:2685438]		
Gm37570	predicted gene, 37570 [Source:MGI Symbol;Acc:MGI:5610798]	0.992	1.223488e-02
Gem	GTP binding protein (gene overexpressed in skeletal muscle) [Source:MGI Symbol;Acc:MGI:99844]	0.991	1.995258e-02
Gsdma	gasdermin A [Source:MGI Symbol;Acc:MGI:1889509]	0.991	1.803658e-02
Lhfp	lipoma HMGIC fusion partner [Source:MGI Symbol;Acc:MGI:1920048]	0.986	1.328003e-02
Gm3848	predicted gene 3848 [Source:MGI Symbol;Acc:MGI:3782020]	0.986	2.263785e-02
Gltp	glycolipid transfer protein [Source:MGI Symbol;Acc:MGI:1929253]	0.985	2.345121e-02
Dock11	dedicator of cytokinesis 11 [Source:MGI Symbol;Acc:MGI:1923224]	0.984	5.221403e-04
Nectin3	nectin cell adhesion molecule 3 [Source:MGI Symbol;Acc:MGI:1930171]	0.982	6.308302e-05
Gm43275	predicted gene 43275 [Source:MGI Symbol;Acc:MGI:5663412]	0.981	1.805736e-02
Tcf4	transcription factor 4 [Source:MGI Symbol;Acc:MGI:98506]	0.981	1.437928e-02
Gm48601	predicted gene, 48601 [Source:MGI Symbol;Acc:MGI:6098180]	0.978	2.446726e-02
Tdrp	testis development related protein [Source:MGI Symbol;Acc:MGI:1919398]	0.977	3.214294e-03

Significant Genes

Runx1 and Runx2 Knock-out

Gene Name	Description	log2 FC	Adj p-value
Gm19402	predicted gene, 19402 [Source:MGI Symbol;Acc:MGI:5011587]	0.975	3.009732e-02
Ppif	peptidylprolyl isomerase F (cyclophilin F) [Source:MGI Symbol;Acc:MGI:2145814]	0.972	3.306386e-02
Zfp677	zinc finger protein 677 [Source:MGI Symbol;Acc:MGI:3053207]	0.972	1.258969e-02
Ptprs	protein tyrosine phosphatase, receptor type, S [Source:MGI Symbol;Acc:MGI:97815]	0.970	5.104165e-03
Gm45294	predicted gene 45294 [Source:MGI Symbol;Acc:MGI:5791130]	0.970	9.170849e-03
Gm37048	predicted gene, 37048 [Source:MGI Symbol;Acc:MGI:5610276]	0.967	8.819159e-03
Gm45292	predicted gene 45292 [Source:MGI Symbol;Acc:MGI:5791128]	0.966	1.649626e-02
Icam1	intercellular adhesion molecule 1 [Source:MGI Symbol;Acc:MGI:96392]	0.965	3.295368e-02
Gm7775	predicted gene 7775 [Source:MGI Symbol;Acc:MGI:3646054]	0.965	1.558888e-02
Man2a2	mannosidase 2, alpha 2 [Source:MGI Symbol;Acc:MGI:2150656]	0.965	3.630199e-05
Gm37285	predicted gene, 37285 [Source:MGI Symbol;Acc:MGI:5610513]	0.964	2.801900e-02
Klhl29	kelch-like 29 [Source:MGI Symbol;Acc:MGI:2683857]	0.963	3.610457e-02
Hcar2	hydroxycarboxylic acid receptor 2 [Source:MGI Symbol;Acc:MGI:1933383]	0.963	1.035903e-02

Significant Genes

Runx1 and Runx2 Knock-out

Gene Name	Description	log2 FC	Adj p-value
Gm10557	predicted gene 10557 [Source:MGI Symbol;Acc:MGI:3708638]	0.959	2.006360e-03
Nr4a1	nuclear receptor subfamily 4, group A, member 1 [Source:MGI Symbol;Acc:MGI:1352454]	0.956	1.866506e-02
Gm38377	predicted gene, 38377 [Source:MGI Symbol;Acc:MGI:5611605]	0.955	2.124864e-02
Mef2a	myocyte enhancer factor 2A [Source:MGI Symbol;Acc:MGI:99532]	0.955	1.335533e-03
Ggta1	glycoprotein galactosyltransferase alpha 1, 3 [Source:MGI Symbol;Acc:MGI:95704]	0.955	3.009732e-02
Asap1	ArfGAP with SH3 domain, ankyrin repeat and PH domain1 [Source:MGI Symbol;Acc:MGI:1342335]	0.953	7.998843e-05
Rasgef1b	RasGEF domain family, member 1B [Source:MGI Symbol;Acc:MGI:2443755]	0.953	7.095902e-03
Marveld1	MARVEL (membrane-associating) domain containing 1 [Source:MGI Symbol;Acc:MGI:2147570]	0.952	7.992631e-05
Il13ra1	interleukin 13 receptor, alpha 1 [Source:MGI Symbol;Acc:MGI:105052]	0.951	1.244089e-02
Chst15	carbohydrate sulfotransferase 15 [Source:MGI Symbol;Acc:MGI:1924840]	0.948	4.305041e-03
Vav3	vav 3 oncogene [Source:MGI Symbol;Acc:MGI:1888518]	0.947	2.279687e-02
Ttc39c	tetratricopeptide repeat domain 39C [Source:MGI Symbol;Acc:MGI:1919997]	0.944	8.893227e-03

Significant Genes

Runx1 and Runx2 Knock-out

Gene Name	Description	log2 FC	Adj p-value
Gm36938	predicted gene, 36938 [Source:MGI Symbol;Acc:MGI:5610166]	0.943	1.430108e-02
Pcdhgc3	protocadherin gamma subfamily C, 3 [Source:MGI Symbol;Acc:MGI:1935201]	0.943	2.295363e-02
Cdc14a	CDC14 cell division cycle 14A [Source:MGI Symbol;Acc:MGI:2442676]	0.942	2.610814e-03
Pfdn4	prefoldin 4 [Source:MGI Symbol;Acc:MGI:1923512]	0.942	7.390611e-03
Cxcl12	chemokine (C-X-C motif) ligand 12 [Source:MGI Symbol;Acc:MGI:103556]	0.941	2.586673e-02
Gm10222	predicted gene 10222 [Source:MGI Symbol;Acc:MGI:3642643]	0.937	3.634571e-02
Tenm4	teneurin transmembrane protein 4 [Source:MGI Symbol;Acc:MGI:2447063]	0.937	1.803658e-02
Ttyh2	tweety family member 2 [Source:MGI Symbol;Acc:MGI:2157091]	0.936	2.785861e-04
Trim11	tripartite motif-containing 11 [Source:MGI Symbol;Acc:MGI:2137355]	0.936	2.675757e-03
Aopep	aminopeptidase O [Source:MGI Symbol;Acc:MGI:1919311]	0.936	7.933910e-03
Gm26688	predicted gene, 26688 [Source:MGI Symbol;Acc:MGI:5477182]	0.935	1.331016e-02
Itga5	integrin alpha 5 (fibronectin receptor alpha) [Source:MGI Symbol;Acc:MGI:96604]	0.935	2.214071e-03
Bdh1	3-hydroxybutyrate dehydrogenase, type 1 [Source:MGI Symbol;Acc:MGI:1919161]	0.934	1.223044e-02

Significant Genes

Runx1 and Runx2 Knock-out

Gene Name	Description	log2 FC	Adj p-value
Kif3c	kinesin family member 3C [Source:MGI Symbol;Acc:MGI:107979]	0.934	1.244199e-04
Gm37755	predicted gene, 37755 [Source:MGI Symbol;Acc:MGI:5610983]	0.934	2.638783e-02
Sec24d	Sec24 related gene family, member D (S. cerevisiae) [Source:MGI Symbol;Acc:MGI:1916858]	0.932	4.022009e-05
Gm11944	predicted gene 11944 [Source:MGI Symbol;Acc:MGI:3702312]	0.928	3.248234e-02
Adgrg1	adhesion G protein-coupled receptor G1 [Source:MGI Symbol;Acc:MGI:1340051]	0.927	1.505910e-02
Ngfr	nerve growth factor receptor (TNFR superfamily, member 16) [Source:MGI Symbol;Acc:MGI:97323]	0.925	4.229172e-02
Fgf2	fibroblast growth factor 2 [Source:MGI Symbol;Acc:MGI:95516]	0.925	2.820613e-03
Gm44667	predicted gene 44667 [Source:MGI Symbol;Acc:MGI:5753243]	0.924	2.160127e-02
Sh3kbp1	SH3-domain kinase binding protein 1 [Source:MGI Symbol;Acc:MGI:1889583]	0.924	9.362960e-04
Sema3b	sema domain, immunoglobulin domain (Ig), short basic domain, secreted, (semaphorin) 3B [Source:MGI Symbol;Acc:MGI:107561]	0.924	1.410385e-02
Gm6081	predicted gene 6081 [Source:MGI Symbol;Acc:MGI:3645683]	0.924	4.927582e-02
Impact	impact, RWD domain protein [Source:MGI Symbol;Acc:MGI:1098233]	0.922	2.857632e-03

Significant Genes

Runx1 and Runx2 Knock-out

Gene Name	Description	log2 FC	Adj p-value
Rasl11b	RAS-like, family 11, member B [Source:MGI Symbol;Acc:MGI:1916189]	0.922	1.110460e-02
Palmd	palmdelphin [Source:MGI Symbol;Acc:MGI:2148896]	0.921	2.686152e-05
Bard1	BRCA1 associated RING domain 1 [Source:MGI Symbol;Acc:MGI:1328361]	0.918	9.721574e-08
Hmgcs1	3-hydroxy-3-methylglutaryl-Coenzyme A synthase 1 [Source:MGI Symbol;Acc:MGI:107592]	0.915	3.857570e-02
Steap4	STEAP family member 4 [Source:MGI Symbol;Acc:MGI:1923560]	0.913	2.012080e-04
Edaradd	EDAR (ectodysplasin-A receptor)-associated death domain [Source:MGI Symbol;Acc:MGI:1931001]	0.912	1.277782e-02
Gm15832	predicted gene 15832 [Source:MGI Symbol;Acc:MGI:3834078]	0.912	2.185427e-02
Kcnc4	potassium voltage gated channel, Shaw-related subfamily, member 4 [Source:MGI Symbol;Acc:MGI:96670]	0.908	4.571135e-02
Prkacb	protein kinase, cAMP dependent, catalytic, beta [Source:MGI Symbol;Acc:MGI:97594]	0.908	1.626559e-03
Rgs12	regulator of G-protein signaling 12 [Source:MGI Symbol;Acc:MGI:1918979]	0.907	1.497947e-02
Cyp26b1	cytochrome P450, family 26, subfamily b, polypeptide 1 [Source:MGI Symbol;Acc:MGI:2176159]	0.906	1.025874e-02
Il17rc	interleukin 17 receptor C [Source:MGI Symbol;Acc:MGI:2159336]	0.905	1.879884e-02

Significant Genes

Runx1 and Runx2 Knock-out

Gene Name	Description	log2 FC	Adj p-value
Prdx6	peroxiredoxin 6 [Source:MGI Symbol;Acc:MGI:894320]	0.905	1.250329e-02
Macroh2a2	macroH2A.2 histone [Source:MGI Symbol;Acc:MGI:3037658]	0.903	2.862322e-02
Rassf8	Ras association (RalGDS/AF-6) domain family (N-terminal) member 8 [Source:MGI Symbol;Acc:MGI:1918573]	0.903	1.131869e-02
Gm17207	predicted gene 17207 [Source:MGI Symbol;Acc:MGI:4938034]	0.901	2.425561e-02
Gm42571	predicted gene 42571 [Source:MGI Symbol;Acc:MGI:5662708]	0.901	1.429798e-02
C030005K06Rik	RIKEN cDNA C030005K06 gene [Source:MGI Symbol;Acc:MGI:1925945]	0.900	8.412372e-03
Ppp1r18	protein phosphatase 1, regulatory subunit 18 [Source:MGI Symbol;Acc:MGI:1923698]	0.899	8.423430e-04
Cpxm2	carboxypeptidase X 2 (M14 family) [Source:MGI Symbol;Acc:MGI:1926006]	0.896	4.571135e-02
Plpp3	phospholipid phosphatase 3 [Source:MGI Symbol;Acc:MGI:1915166]	0.892	4.590561e-02
Rnf149	ring finger protein 149 [Source:MGI Symbol;Acc:MGI:2677438]	0.891	3.827954e-02
Socs3	suppressor of cytokine signaling 3 [Source:MGI Symbol;Acc:MGI:1201791]	0.890	4.100337e-02
Ttc9	tetratricopeptide repeat domain 9 [Source:MGI Symbol;Acc:MGI:1916730]	0.889	2.683928e-02
Schip1	schwannomin interacting protein 1 [Source:MGI Symbol;Acc:MGI:1353557]	0.886	1.412437e-04

Significant Genes

Runx1 and Runx2 Knock-out

Gene Name	Description	log2 FC	Adj p-value
Mctp2	multiple C2 domains, transmembrane 2 [Source:MGI Symbol;Acc:MGI:2685335]	0.885	1.571334e-02
Gm38251	predicted gene, 38251 [Source:MGI Symbol;Acc:MGI:5611479]	0.881	1.438728e-02
Pbx3	pre B cell leukemia homeobox 3 [Source:MGI Symbol;Acc:MGI:97496]	0.880	4.713292e-04
Eya1	EYA transcriptional coactivator and phosphatase 1 [Source:MGI Symbol;Acc:MGI:109344]	0.877	3.369974e-03
Gcc1	golgi coiled coil 1 [Source:MGI Symbol;Acc:MGI:1921625]	0.877	2.348158e-03
**Lacc1**	**laccase domain containing 1 [Source:MGI Symbol;Acc:MGI:2445077]**	**0.877**	**9.056731e-04**
Gm43019	predicted gene 43019 [Source:MGI Symbol;Acc:MGI:5663156]	0.876	4.442660e-02
Slc16a10	solute carrier family 16 (monocarboxylic acid transporters), member 10 [Source:MGI Symbol;Acc:MGI:1919722]	0.875	2.412463e-02
Gk	glycerol kinase [Source:MGI Symbol;Acc:MGI:106594]	0.871	3.496950e-02
Ankrd29	ankyrin repeat domain 29 [Source:MGI Symbol;Acc:MGI:2687055]	0.870	2.058017e-02
2610035D17Rik	RIKEN cDNA 2610035D17 gene [Source:MGI Symbol;Acc:MGI:1919636]	0.870	3.096819e-02
Epgn	epithelial mitogen [Source:MGI Symbol;Acc:MGI:1919170]	0.869	1.558888e-02
Gm45640	predicted gene 45640 [Source:MGI Symbol;Acc:MGI:5791476]	0.866	3.124982e-02

Significant Genes

Runx1 and Runx2 Knock-out

Gene Name	Description	log2 FC	Adj p-value
Plcl1	phospholipase C-like 1 [Source:MGI Symbol;Acc:MGI:3036262]	0.866	4.091891e-02
Epha4	Eph receptor A4 [Source:MGI Symbol;Acc:MGI:98277]	0.865	2.283125e-02
Fblim1	filamin binding LIM protein 1 [Source:MGI Symbol;Acc:MGI:1921452]	0.864	3.820296e-04
Gm43807	predicted gene 43807 [Source:MGI Symbol;Acc:MGI:5663944]	0.864	4.242094e-02
Cenpa	centromere protein A [Source:MGI Symbol;Acc:MGI:88375]	0.863	1.359883e-04
Dtl	denticleless E3 ubiquitin protein ligase [Source:MGI Symbol;Acc:MGI:1924093]	0.862	6.984356e-04
Fam43a	family with sequence similarity 43, member A [Source:MGI Symbol;Acc:MGI:2676309]	0.862	3.784957e-02
Gm14853	predicted gene 14853 [Source:MGI Symbol;Acc:MGI:3708095]	0.862	2.627759e-02
Gm42892	predicted gene 42892 [Source:MGI Symbol;Acc:MGI:5663029]	0.861	1.091794e-02
Map3k14	mitogen-activated protein kinase kinase kinase 14 [Source:MGI Symbol;Acc:MGI:1858204]	0.861	9.815844e-05
Gm8319	predicted gene 8319 [Source:MGI Symbol;Acc:MGI:3646693]	0.860	2.939665e-02
Map3k5	mitogen-activated protein kinase kinase kinase 5 [Source:MGI Symbol;Acc:MGI:1346876]	0.859	2.004778e-02
Capn2	calpain 2 [Source:MGI Symbol;Acc:MGI:88264]	0.855	1.215779e-02

Significant Genes

Runx1 and Runx2 Knock-out

Gene Name	Description	log2 FC	Adj p-value
Gm28661	predicted gene 28661 [Source:MGI Symbol;Acc:MGI:5579367]	0.854	2.297039e-02
Stil	Scl/Tal1 interrupting locus [Source:MGI Symbol;Acc:MGI:107477]	0.854	4.701300e-05
Usp24	ubiquitin specific peptidase 24 [Source:MGI Symbol;Acc:MGI:1919936]	0.853	7.933910e-03
Has1	hyaluronan synthase 1 [Source:MGI Symbol;Acc:MGI:106590]	0.852	3.904100e-02
Arl4a	ADP-ribosylation factor-like 4A [Source:MGI Symbol;Acc:MGI:99437]	0.851	2.631300e-03
Mllt3	myeloid/lymphoid or mixed-lineage leukemia; translocated to, 3 [Source:MGI Symbol;Acc:MGI:1917372]	0.850	5.564188e-03
Gm8378	predicted gene 8378 [Source:MGI Symbol;Acc:MGI:3646777]	0.849	9.618245e-04
Gsta4	glutathione S-transferase, alpha 4 [Source:MGI Symbol;Acc:MGI:1309515]	0.849	4.571135e-02
Cadm1	cell adhesion molecule 1 [Source:MGI Symbol;Acc:MGI:1889272]	0.848	1.891636e-04
Gm45221	predicted gene 45221 [Source:MGI Symbol;Acc:MGI:5753797]	0.848	1.624537e-02
**Axl**	**AXL receptor tyrosine kinase [Source:MGI Symbol;Acc:MGI:1347244]**	**0.847**	**1.849490e-03**
Wdr91	WD repeat domain 91 [Source:MGI Symbol;Acc:MGI:2141558]	0.845	1.298054e-03
Knl1	kinetochore scaffold 1 [Source:MGI Symbol;Acc:MGI:1923714]	0.845	1.879884e-02

Significant Genes

Runx1 and Runx2 Knock-out

Gene Name	Description	log2 FC	Adj p-value
Gm49125	predicted gene, 49125 [Source:MGI Symbol;Acc:MGI:6118530]	0.842	1.826605e-02
Rnf141	ring finger protein 141 [Source:MGI Symbol;Acc:MGI:1914400]	0.841	7.059915e-07
Gm43581	predicted gene 43581 [Source:MGI Symbol;Acc:MGI:5663718]	0.840	3.166884e-02
Psat1	phosphoserine aminotransferase 1 [Source:MGI Symbol;Acc:MGI:2183441]	0.839	4.691692e-02
Gm4876	predicted gene 4876 [Source:MGI Symbol;Acc:MGI:3647654]	0.838	3.358520e-03
Piga	phosphatidylinositol glycan anchor biosynthesis, class A [Source:MGI Symbol;Acc:MGI:99461]	0.837	1.592817e-04
Gm43187	predicted gene 43187 [Source:MGI Symbol;Acc:MGI:5663324]	0.837	2.694799e-02
Septin10	septin 10 [Source:MGI Symbol;Acc:MGI:1918110]	0.835	5.595748e-03
Smad1	SMAD family member 1 [Source:MGI Symbol;Acc:MGI:109452]	0.833	1.656728e-02
Cxadr	coxsackie virus and adenovirus receptor [Source:MGI Symbol;Acc:MGI:1201679]	0.833	3.925454e-02
Zfp386	zinc finger protein 386 (Kruppel-like) [Source:MGI Symbol;Acc:MGI:1930708]	0.830	8.070655e-03
2610307P16Rik	RIKEN cDNA 2610307P16 gene [Source:MGI Symbol;Acc:MGI:1919768]	0.828	2.236511e-02
Gxylt2	glucoside xylosyltransferase 2 [Source:MGI Symbol;Acc:MGI:2682940]	0.824	3.295483e-02

Significant Genes

Runx1 and Runx2 Knock-out

Gene Name	Description	log2 FC	Adj p-value
Plek2	pleckstrin 2 [Source:MGI Symbol;Acc:MGI:1351466]	0.824	1.735890e-05
Tsc22d1	TSC22 domain family, member 1 [Source:MGI Symbol;Acc:MGI:109127]	0.821	2.949228e-02
Pdlim7	PDZ and LIM domain 7 [Source:MGI Symbol;Acc:MGI:1914649]	0.820	6.779876e-03
Gm45289	predicted gene 45289 [Source:MGI Symbol;Acc:MGI:5791125]	0.818	3.526310e-02
Zbtb10	zinc finger and BTB domain containing 10 [Source:MGI Symbol;Acc:MGI:2139883]	0.816	1.123985e-02
Gm13092	predicted gene 13092 [Source:MGI Symbol;Acc:MGI:3649661]	0.816	4.336610e-02
Ifnar1	interferon (alpha and beta) receptor 1 [Source:MGI Symbol;Acc:MGI:107658]	0.813	1.350513e-03
Vangl1	VANGL planar cell polarity 1 [Source:MGI Symbol;Acc:MGI:2159344]	0.811	2.853954e-04
Ckap2	cytoskeleton associated protein 2 [Source:MGI Symbol;Acc:MGI:1931797]	0.810	2.044069e-04
Rbbp8	retinoblastoma binding protein 8, endonuclease [Source:MGI Symbol;Acc:MGI:2442995]	0.804	3.115520e-02
Itpk1	inositol 1,3,4-triphosphate 5/6 kinase [Source:MGI Symbol;Acc:MGI:2446159]	0.802	2.635441e-02
Gm2366	predicted gene 2366 [Source:MGI Symbol;Acc:MGI:3780534]	0.801	4.416047e-03
Gria3	glutamate receptor, ionotropic, AMPA3 (alpha 3) [Source:MGI Symbol;Acc:MGI:95810]	0.801	2.638557e-02

Significant Genes

Runx1 and Runx2 Knock-out

Gene Name	Description	log2 FC	Adj p-value
Gm28439	predicted gene 28439 [Source:MGI Symbol;Acc:MGI:5579145]	0.799	2.642686e-02
Mybl2	myeloblastosis oncogene-like 2 [Source:MGI Symbol;Acc:MGI:101785]	0.799	1.704936e-03
Mb21d2	Mab-21 domain containing 2 [Source:MGI Symbol;Acc:MGI:1917028]	0.794	1.991900e-03
Eya2	EYA transcriptional coactivator and phosphatase 2 [Source:MGI Symbol;Acc:MGI:109341]	0.794	8.826262e-03
Ltb4r1	leukotriene B4 receptor 1 [Source:MGI Symbol;Acc:MGI:1309472]	0.789	2.923814e-02
Ttc39b	tetratricopeptide repeat domain 39B [Source:MGI Symbol;Acc:MGI:1917113]	0.789	1.239941e-02
Ssbp2	single-stranded DNA binding protein 2 [Source:MGI Symbol;Acc:MGI:1914220]	0.786	3.118967e-03
Lpin2	lipin 2 [Source:MGI Symbol;Acc:MGI:1891341]	0.786	4.307626e-03
Gm44891	predicted gene 44891 [Source:MGI Symbol;Acc:MGI:5753467]	0.785	3.731256e-02
Aspm	abnormal spindle microtubule assembly [Source:MGI Symbol;Acc:MGI:1334448]	0.779	1.460344e-02
Dlx4	distal-less homeobox 4 [Source:MGI Symbol;Acc:MGI:94904]	0.776	3.663173e-02
Plk2	polo like kinase 2 [Source:MGI Symbol;Acc:MGI:1099790]	0.776	1.228241e-02
Coro1c	coronin, actin binding protein 1C [Source:MGI Symbol;Acc:MGI:1345964]	0.775	2.628668e-02

Significant Genes

Runx1 and Runx2 Knock-out

Gene Name	Description	log2 FC	Adj p-value
Csrp2	cysteine and glycine-rich protein 2 [Source:MGI Symbol;Acc:MGI:1202907]	0.775	3.389146e-03
Mapk11	mitogen-activated protein kinase 11 [Source:MGI Symbol;Acc:MGI:1338024]	0.768	2.120300e-02
Urah	urate (5-hydroxyiso-) hydrolase [Source:MGI Symbol;Acc:MGI:1916142]	0.768	3.249410e-02
Pcdhgb5	protocadherin gamma subfamily B, 5 [Source:MGI Symbol;Acc:MGI:1935196]	0.768	1.666066e-02
Narf	nuclear prelamin A recognition factor [Source:MGI Symbol;Acc:MGI:1914858]	0.767	1.225582e-05
Il1rap	interleukin 1 receptor accessory protein [Source:MGI Symbol;Acc:MGI:104975]	0.761	6.060334e-04
Abhd10	abhydrolase domain containing 10 [Source:MGI Symbol;Acc:MGI:2442422]	0.760	8.593739e-03
Gpr161	G protein-coupled receptor 161 [Source:MGI Symbol;Acc:MGI:2685054]	0.757	3.084043e-04
Neto2	neuropilin (NRP) and tolloid (TLL)-like 2 [Source:MGI Symbol;Acc:MGI:1921763]	0.756	9.147887e-03
Idh1	isocitrate dehydrogenase 1 (NADP+), soluble [Source:MGI Symbol;Acc:MGI:96413]	0.755	6.854660e-03
Hspg2	perlecan (heparan sulfate proteoglycan 2) [Source:MGI Symbol;Acc:MGI:96257]	0.753	4.384960e-02
Fam49b	family with sequence similarity 49, member B [Source:MGI Symbol;Acc:MGI:1923520]	0.752	3.061981e-02
Pros1	protein S (alpha) [Source:MGI Symbol;Acc:MGI:1095733]	0.750	2.044069e-04

Significant Genes

Runx1 and Runx2 Knock-out

Gene Name	Description	log2 FC	Adj p-value
Prss48	protease, serine 48 [Source:MGI Symbol;Acc:MGI:2685865]	0.748	3.416060e-02
Dctn4	dynactin 4 [Source:MGI Symbol;Acc:MGI:1914915]	0.747	1.338855e-02
Lrrfip2	leucine rich repeat (in FLII) interacting protein 2 [Source:MGI Symbol;Acc:MGI:1918518]	0.747	1.409198e-02
Gm43679	predicted gene 43679 [Source:MGI Symbol;Acc:MGI:5663816]	0.745	7.390611e-03
Tspan3	tetraspanin 3 [Source:MGI Symbol;Acc:MGI:1928098]	0.745	1.492042e-02
Npc2	NPC intracellular cholesterol transporter 2 [Source:MGI Symbol;Acc:MGI:1915213]	0.742	2.057334e-02
Homer3	homer scaffolding protein 3 [Source:MGI Symbol;Acc:MGI:1347359]	0.740	6.763934e-03
Lrig3	leucine-rich repeats and immunoglobulin-like domains 3 [Source:MGI Symbol;Acc:MGI:2443955]	0.739	1.179504e-03
Zfp566	zinc finger protein 566 [Source:MGI Symbol;Acc:MGI:1919806]	0.735	1.551598e-02
Rbfox2	RNA binding protein, fox-1 homolog (C. elegans) 2 [Source:MGI Symbol;Acc:MGI:1933973]	0.735	7.200606e-03
Acpp	acid phosphatase, prostate [Source:MGI Symbol;Acc:MGI:1928480]	0.734	1.681783e-02
Enpp1	ectonucleotide pyrophosphatase/phosphodiesterase 1 [Source:MGI Symbol;Acc:MGI:97370]	0.734	4.238787e-02

Significant Genes

Runx1 and Runx2 Knock-out

Gene Name	Description	log2 FC	Adj p-value
Dnajb5	DnaJ heat shock protein family (Hsp40) member B5 [Source:MGI Symbol;Acc:MGI:1930018]	0.733	8.492290e-03
Gm43274	predicted gene 43274 [Source:MGI Symbol;Acc:MGI:5663411]	0.731	4.660532e-02
Mcam	melanoma cell adhesion molecule [Source:MGI Symbol;Acc:MGI:1933966]	0.730	2.403005e-02
Stx7	syntaxin 7 [Source:MGI Symbol;Acc:MGI:1858210]	0.726	9.500297e-03
Pcdhgc4	protocadherin gamma subfamily C, 4 [Source:MGI Symbol;Acc:MGI:1935203]	0.724	1.338855e-02
Filip1l	filamin A interacting protein 1-like [Source:MGI Symbol;Acc:MGI:1925999]	0.724	1.101968e-03
Cux1	cut-like homeobox 1 [Source:MGI Symbol;Acc:MGI:88568]	0.722	3.816116e-03
Itga3	integrin alpha 3 [Source:MGI Symbol;Acc:MGI:96602]	0.721	4.881847e-02
Hs6st1	heparan sulfate 6-O-sulfotransferase 1 [Source:MGI Symbol;Acc:MGI:1354958]	0.721	4.451001e-02
Slco3a1	solute carrier organic anion transporter family, member 3a1 [Source:MGI Symbol;Acc:MGI:1351867]	0.720	7.795827e-03
Omg	oligodendrocyte myelin glycoprotein [Source:MGI Symbol;Acc:MGI:106586]	0.720	1.250329e-02
Gm45537	predicted gene 45537 [Source:MGI Symbol;Acc:MGI:5791373]	0.719	4.899793e-02
Nfe2l2	nuclear factor, erythroid derived 2, like 2 [Source:MGI Symbol;Acc:MGI:108420]	0.717	3.880954e-02

Significant Genes

Runx1 and Runx2 Knock-out

Gene Name	Description	log2 FC	Adj p-value
Fdft1	farnesyl diphosphate farnesyl transferase 1 [Source:MGI Symbol;Acc:MGI:102706]	0.717	4.399283e-02
Net1	neuroepithelial cell transforming gene 1 [Source:MGI Symbol;Acc:MGI:1927138]	0.716	4.714570e-02
Pcdhgb4	protocadherin gamma subfamily B, 4 [Source:MGI Symbol;Acc:MGI:1935173]	0.715	3.841725e-02
Disp1	dispatched RND transporter family member 1 [Source:MGI Symbol;Acc:MGI:1916147]	0.712	4.138712e-02
Eva1a	eva-1 homolog A (C. elegans) [Source:MGI Symbol;Acc:MGI:2385247]	0.711	2.236402e-02
Pola1	polymerase (DNA directed), alpha 1 [Source:MGI Symbol;Acc:MGI:99660]	0.708	1.205359e-02
Mapre2	microtubule-associated protein, RP/EB family, member 2 [Source:MGI Symbol;Acc:MGI:106271]	0.704	1.339671e-02
Slc19a1	solute carrier family 19 (folate transporter), member 1 [Source:MGI Symbol;Acc:MGI:103182]	0.703	2.550363e-02
Pinx1	PIN2/TERF1 interacting, telomerase inhibitor 1 [Source:MGI Symbol;Acc:MGI:1919650]	0.701	3.469350e-04
Tnik	TRAF2 and NCK interacting kinase [Source:MGI Symbol;Acc:MGI:1916264]	0.699	7.904282e-04
Tiam1	T cell lymphoma invasion and metastasis 1 [Source:MGI Symbol;Acc:MGI:103306]	0.698	4.307555e-02
1700025G04Rik	RIKEN cDNA 1700025G04 gene [Source:MGI Symbol;Acc:MGI:1916649]	0.698	1.977087e-03
**6530402F18Rik**	**RIKEN cDNA 6530402F18 gene [Source:MGI Symbol;Acc:MGI:1923470]**	**0.696**	**3.620619e-02**

Significant Genes

Runx1 and Runx2 Knock-out

Gene Name	Description	log2 FC	Adj p-value
Gm37802	predicted gene, 37802 [Source:MGI Symbol;Acc:MGI:5611030]	0.695	4.530012e-02
Clec2d	C-type lectin domain family 2, member d [Source:MGI Symbol;Acc:MGI:2135589]	0.695	2.905871e-02
Tes	testis derived transcript [Source:MGI Symbol;Acc:MGI:105081]	0.694	4.126566e-02
Sh3d19	SH3 domain protein D19 [Source:MGI Symbol;Acc:MGI:1350923]	0.693	2.537490e-02
Gm48600	predicted gene, 48600 [Source:MGI Symbol;Acc:MGI:6098178]	0.692	4.821748e-02
Ccdc88a	coiled coil domain containing 88A [Source:MGI Symbol;Acc:MGI:1925177]	0.691	1.396144e-02
Msi1	musashi RNA-binding protein 1 [Source:MGI Symbol;Acc:MGI:107376]	0.689	1.630435e-02
Nckap5l	NCK-associated protein 5-like [Source:MGI Symbol;Acc:MGI:3609653]	0.689	4.571135e-02
Caskin2	CASK-interacting protein 2 [Source:MGI Symbol;Acc:MGI:2157062]	0.688	8.229272e-03
Egln1	egl-9 family hypoxia-inducible factor 1 [Source:MGI Symbol;Acc:MGI:1932286]	0.687	7.076969e-03
Frmd4a	FERM domain containing 4A [Source:MGI Symbol;Acc:MGI:1919850]	0.686	2.013647e-02
Nfatc1	nuclear factor of activated T cells, cytoplasmic, calcineurin dependent 1 [Source:MGI Symbol;Acc:MGI:102469]	0.685	2.065342e-02
Slc23a2	solute carrier family 23 (nucleobase transporters), member 2 [Source:MGI Symbol;Acc:MGI:1859682]	0.684	1.397085e-02

Significant Genes

Runx1 and Runx2 Knock-out

Gene Name	Description	log2 FC	Adj p-value
Mtmr11	myotubularin related protein 11 [Source:MGI Symbol;Acc:MGI:2652817]	0.682	1.423084e-02
Zfp462	zinc finger protein 462 [Source:MGI Symbol;Acc:MGI:107690]	0.682	8.822965e-03
Lima1	LIM domain and actin binding 1 [Source:MGI Symbol;Acc:MGI:1920992]	0.681	1.773091e-02
Slc25a27	solute carrier family 25, member 27 [Source:MGI Symbol;Acc:MGI:1921261]	0.678	9.788464e-04
Dpysl2	dihydropyrimidinase-like 2 [Source:MGI Symbol;Acc:MGI:1349763]	0.676	2.801900e-02
Zwint	ZW10 interactor [Source:MGI Symbol;Acc:MGI:1289227]	0.674	2.666213e-02
Vdr	vitamin D (1,25-dihydroxyvitamin D3) receptor [Source:MGI Symbol;Acc:MGI:103076]	0.671	2.787416e-02
Lzts3	leucine zipper, putative tumor suppressor family member 3 [Source:MGI Symbol;Acc:MGI:2656976]	0.670	2.302708e-02
Zfp365	zinc finger protein 365 [Source:MGI Symbol;Acc:MGI:2143676]	0.669	1.166893e-02
Nipal4	NIPA-like domain containing 4 [Source:MGI Symbol;Acc:MGI:2444671]	0.667	2.609178e-02
Trpv4	transient receptor potential cation channel, subfamily V, member 4 [Source:MGI Symbol;Acc:MGI:1926945]	0.662	2.061744e-02
Rab11fip2	RAB11 family interacting protein 2 (class I) [Source:MGI Symbol;Acc:MGI:1922248]	0.660	1.363086e-02
Nckap5los	NCK-associated protein 5-like, opposite strand [Source:MGI Symbol;Acc:MGI:1918162]	0.654	3.979738e-02

Significant Genes

Runx1 and Runx2 Knock-out

Gene Name	Description	log2 FC	Adj p-value
Ccdc58	coiled-coil domain containing 58 [Source:MGI Symbol;Acc:MGI:2146423]	0.652	4.347219e-03
Gm49759	predicted gene, 49759 [Source:MGI Symbol;Acc:MGI:6215263]	0.650	2.237845e-02
Nbl1	NBL1, DAN family BMP antagonist [Source:MGI Symbol;Acc:MGI:104591]	0.650	2.465238e-02
Lrrc8c	leucine rich repeat containing 8 family, member C [Source:MGI Symbol;Acc:MGI:2140839]	0.649	7.934281e-03
Prxl2a	peroxiredoxin like 2A [Source:MGI Symbol;Acc:MGI:1917814]	0.647	7.232250e-03
Tia1	cytotoxic granule-associated RNA binding protein 1 [Source:MGI Symbol;Acc:MGI:107914]	0.643	5.708471e-03
Cenpe	centromere protein E [Source:MGI Symbol;Acc:MGI:1098230]	0.643	2.542925e-02
Anxa11	annexin A11 [Source:MGI Symbol;Acc:MGI:108481]	0.640	2.763832e-02
Fam126a	family with sequence similarity 126, member A [Source:MGI Symbol;Acc:MGI:2149839]	0.639	1.485511e-03
Gm49968	predicted gene, 49968 [Source:MGI Symbol;Acc:MGI:6275235]	0.638	2.793357e-02
Slc12a7	solute carrier family 12, member 7 [Source:MGI Symbol;Acc:MGI:1342283]	0.635	6.075862e-04
Mxi1	MAX interactor 1, dimerization protein [Source:MGI Symbol;Acc:MGI:97245]	0.634	4.931565e-02
Bloc1s2	biogenesis of lysosomal organelles complex-1, subunit 2 [Source:MGI	0.632	2.769228e-02

Significant Genes<br>Runx1 and Runx2 Knock-out			
Gene Name	Description	log2 FC	Adj p-value
	Symbol;Acc:MGI:1920939]		
Slc6a6	solute carrier family 6 (neurotransmitter transporter, taurine), member 6 [Source:MGI Symbol;Acc:MGI:98488]	0.632	1.634855e-02
Acad11	acyl-Coenzyme A dehydrogenase family, member 11 [Source:MGI Symbol;Acc:MGI:2143169]	0.631	2.911276e-06
Ankrd10	ankyrin repeat domain 10 [Source:MGI Symbol;Acc:MGI:1921840]	0.630	2.087615e-02
Acvr2b	activin receptor IIB [Source:MGI Symbol;Acc:MGI:87912]	0.627	4.204233e-02
Acadsb	acyl-Coenzyme A dehydrogenase, short/branched chain [Source:MGI Symbol;Acc:MGI:1914135]	0.627	8.400569e-04
Gm50069	predicted gene, 50069 [Source:MGI Symbol;Acc:MGI:6275393]	0.626	1.515510e-02
Arid5b	AT rich interactive domain 5B (MRF1-like) [Source:MGI Symbol;Acc:MGI:2175912]	0.614	4.912345e-02
Ckap2l	cytoskeleton associated protein 2-like [Source:MGI Symbol;Acc:MGI:1917716]	0.607	2.801900e-02
Hmgb1-ps1	high mobility group box 1, pseudogene 1 [Source:MGI Symbol;Acc:MGI:3768536]	0.604	4.821748e-02
Mitd1	MIT, microtubule interacting and transport, domain containing 1 [Source:MGI Symbol;Acc:MGI:1916278]	0.602	5.786813e-04
Rap1gds1	RAP1, GTP-GDP dissociation stimulator 1 [Source:MGI Symbol;Acc:MGI:2385189]	0.600	3.535504e-02

Significant Genes

Runx1 and Runx2 Knock-out

Gene Name	Description	log2 FC	Adj p-value
Emsy	EMSY, BRCA2-interacting transcriptional repressor [Source:MGI Symbol;Acc:MGI:1924203]	0.599	1.352894e-03
Kif18a	kinesin family member 18A [Source:MGI Symbol;Acc:MGI:2446977]	0.599	1.131869e-02
Zfp292	zinc finger protein 292 [Source:MGI Symbol;Acc:MGI:1353423]	0.596	2.655975e-02
Gm44668	predicted gene 44668 [Source:MGI Symbol;Acc:MGI:5753244]	0.592	4.691692e-02
Psrc1	proline/serine-rich coiled-coil 1 [Source:MGI Symbol;Acc:MGI:1913099]	0.590	2.057334e-02
Adgrl3	adhesion G protein-coupled receptor L3 [Source:MGI Symbol;Acc:MGI:2441950]	0.589	3.248234e-02
Maoa	monoamine oxidase A [Source:MGI Symbol;Acc:MGI:96915]	0.586	1.120475e-02
Ipo13	importin 13 [Source:MGI Symbol;Acc:MGI:2385205]	0.586	5.470169e-04
Snapc3	small nuclear RNA activating complex, polypeptide 3 [Source:MGI Symbol;Acc:MGI:1916338]	0.586	4.777001e-03
Letm2	leucine zipper-EF-hand containing transmembrane protein 2 [Source:MGI Symbol;Acc:MGI:2444979]	−0.587	3.453990e-02
Zdhhc14	zinc finger, DHHC domain containing 14 [Source:MGI Symbol;Acc:MGI:2653229]	−0.588	2.694988e-02
Lrrfip1	leucine rich repeat (in FLII) interacting protein 1 [Source:MGI Symbol;Acc:MGI:1342770]	−0.589	9.761458e-03

Significant Genes

Runx1 and Runx2 Knock-out

Gene Name	Description	log2 FC	Adj p-value
Col4a6	collagen, type IV, alpha 6 [Source:MGI Symbol;Acc:MGI:2152695]	−0.593	2.018604e-02
5830432E09Rik	RIKEN cDNA 5830432E09 gene [Source:MGI Symbol;Acc:MGI:1915015]	−0.594	4.326979e-02
Msrb3	methionine sulfoxide reductase B3 [Source:MGI Symbol;Acc:MGI:2443538]	−0.599	3.048207e-02
Dact2	dishevelled-binding antagonist of beta-catenin 2 [Source:MGI Symbol;Acc:MGI:1920347]	−0.600	2.862322e-02
Kcnc3	potassium voltage gated channel, Shaw-related subfamily, member 3 [Source:MGI Symbol;Acc:MGI:96669]	−0.611	7.629189e-03
Gm43499	predicted gene 43499 [Source:MGI Symbol;Acc:MGI:5663636]	−0.618	3.708144e-02
Ccdc120	coiled-coil domain containing 120 [Source:MGI Symbol;Acc:MGI:1859619]	−0.623	2.801900e-02
Zfp568	zinc finger protein 568 [Source:MGI Symbol;Acc:MGI:2142347]	−0.627	3.304296e-03
Mturn	maturin, neural progenitor differentiation regulator homolog (Xenopus) [Source:MGI Symbol;Acc:MGI:1915485]	−0.628	4.753325e-02
Ptprk	protein tyrosine phosphatase, receptor type, K [Source:MGI Symbol;Acc:MGI:103310]	−0.630	2.489904e-02
Dclk1	doublecortin-like kinase 1 [Source:MGI Symbol;Acc:MGI:1330861]	−0.631	1.126604e-02
Cers2	ceramide synthase 2 [Source:MGI Symbol;Acc:MGI:1924143]	−0.632	1.655628e-02

Significant Genes<br>Runx1 and Runx2 Knock-out			
Gene Name	Description	log2 FC	Adj p-value
Myadm	myeloid-associated differentiation marker [Source:MGI Symbol;Acc:MGI:1355332]	−0.633	1.988683e-02
Scly	selenocysteine lyase [Source:MGI Symbol;Acc:MGI:1355310]	−0.641	4.384960e-02
Dnajb13	DnaJ heat shock protein family (Hsp40) member B13 [Source:MGI Symbol;Acc:MGI:1916637]	−0.646	2.473061e-03
Pkig	protein kinase inhibitor, gamma [Source:MGI Symbol;Acc:MGI:1343086]	−0.647	1.040366e-03
Sort1	sortilin 1 [Source:MGI Symbol;Acc:MGI:1338015]	−0.650	5.330508e-03
Pbxip1	pre B cell leukemia transcription factor interacting protein 1 [Source:MGI Symbol;Acc:MGI:2441670]	−0.655	2.178933e-03
Nod1	nucleotide-binding oligomerization domain containing 1 [Source:MGI Symbol;Acc:MGI:1341839]	−0.656	4.416047e-03
Plcd3	phospholipase C, delta 3 [Source:MGI Symbol;Acc:MGI:107451]	−0.666	8.229272e-03
Frk	fyn-related kinase [Source:MGI Symbol;Acc:MGI:103265]	−0.666	1.201360e-02
Tbc1d4	TBC1 domain family, member 4 [Source:MGI Symbol;Acc:MGI:2429660]	−0.675	1.352894e-03
**Eml5**	**echinoderm microtubule associated protein like 5 [Source:MGI Symbol;Acc:MGI:2442513]**	**−0.676**	**1.626559e-03**
Slc43a2	solute carrier family 43, member 2 [Source:MGI Symbol;Acc:MGI:2442746]	−0.677	3.153252e-02

Significant Genes<br>Runx1 and Runx2 Knock-out			
Gene Name	Description	log2 FC	Adj p-value
Pak3	p21 (RAC1) activated kinase 3 [Source:MGI Symbol;Acc:MGI:1339656]	−0.679	2.358227e-03
Epb41l1	erythrocyte membrane protein band 4.1 like 1 [Source:MGI Symbol;Acc:MGI:103010]	−0.681	6.928448e-03
Arhgef18	rho/rac guanine nucleotide exchange factor (GEF) 18 [Source:MGI Symbol;Acc:MGI:2142567]	−0.690	1.089484e-03
2310030G06Rik	RIKEN cDNA 2310030G06 gene [Source:MGI Symbol;Acc:MGI:1914202]	−0.691	1.718898e-02
Plekha2	pleckstrin homology domain-containing, family A (phosphoinositide binding specific) member 2 [Source:MGI Symbol;Acc:MGI:1928144]	−0.695	1.239941e-02
2610306M01Rik	RIKEN cDNA 2610306M01 gene [Source:MGI Symbol;Acc:MGI:1914420]	−0.696	4.319564e-02
Sdc2	syndecan 2 [Source:MGI Symbol;Acc:MGI:1349165]	−0.698	1.916861e-02
2010300C02Rik	RIKEN cDNA 2010300C02 gene [Source:MGI Symbol;Acc:MGI:1919347]	−0.702	2.881576e-02
B930095G15Rik	RIKEN cDNA B930095G15 gene [Source:MGI Symbol;Acc:MGI:2443701]	−0.703	1.773091e-02
Tmem268	transmembrane protein 268 [Source:MGI Symbol;Acc:MGI:1913920]	−0.705	2.503194e-04
9330188P03Rik	RIKEN cDNA 9330188P03 gene [Source:MGI Symbol;Acc:MGI:2686435]	−0.706	2.701496e-02
Irx1	Iroquois homeobox 1 [Source:MGI Symbol;Acc:MGI:1197515]	−0.708	3.784957e-02

Significant Genes

Runx1 and Runx2 Knock-out

Gene Name	Description	log2 FC	Adj p-value
Tcta	T cell leukemia translocation altered gene [Source:MGI Symbol;Acc:MGI:1918829]	−0.709	1.879884e-02
Abca3	ATP-binding cassette, sub-family A (ABC1), member 3 [Source:MGI Symbol;Acc:MGI:1351617]	−0.723	3.301900e-04
Liph	lipase, member H [Source:MGI Symbol;Acc:MGI:2388029]	−0.723	4.275470e-02
**Eps8l1**	**EPS8-like 1 [Source:MGI Symbol;Acc:MGI:1914675]**	**−0.723**	**2.243988e-03**
Exoc6	exocyst complex component 6 [Source:MGI Symbol;Acc:MGI:1351611]	−0.734	1.095178e-02
Tcn2	transcobalamin 2 [Source:MGI Symbol;Acc:MGI:98534]	−0.734	3.228775e-02
Gm41442	predicted gene, 41442 [Source:MGI Symbol;Acc:MGI:5624327]	−0.735	1.572375e-02
Dlc1	deleted in liver cancer 1 [Source:MGI Symbol;Acc:MGI:1354949]	−0.736	1.086418e-03
Trmo	tRNA methyltransferase O [Source:MGI Symbol;Acc:MGI:1922003]	−0.740	4.510733e-02
Gm45552	predicted gene 45552 [Source:MGI Symbol;Acc:MGI:5791388]	−0.744	2.303390e-02
Tst	thiosulfate sulfurtransferase, mitochondrial [Source:MGI Symbol;Acc:MGI:98852]	−0.745	2.668064e-02
0610040J01Rik	RIKEN cDNA 0610040J01 gene [Source:MGI Symbol;Acc:MGI:1923511]	−0.746	3.711265e-03
Nkpd1	NTPase, KAP family P-loop domain containing 1 [Source:MGI Symbol;Acc:MGI:1916797]	−0.752	4.099825e-02

Significant Genes

Runx1 and Runx2 Knock-out

Gene Name	Description	log2 FC	Adj p-value
Scnn1a	sodium channel, nonvoltage-gated 1 alpha [Source:MGI Symbol;Acc:MGI:101782]	−0.753	2.862322e-02
Rnf39	ring finger protein 39 [Source:MGI Symbol;Acc:MGI:2156378]	−0.756	2.542925e-02
Arhgef16	Rho guanine nucleotide exchange factor (GEF) 16 [Source:MGI Symbol;Acc:MGI:2446219]	−0.763	1.206351e-02
Smpdl3a	sphingomyelin phosphodiesterase, acid-like 3A [Source:MGI Symbol;Acc:MGI:1931437]	−0.765	6.898492e-03
Agpat2	1-acylglycerol-3-phosphate O-acyltransferase 2 (lysophosphatidic acid acyltransferase, beta) [Source:MGI Symbol;Acc:MGI:1914762]	−0.765	6.898492e-03
Fignl2	fidgetin-like 2 [Source:MGI Symbol;Acc:MGI:3646919]	−0.766	4.933759e-02
Ctsh	cathepsin H [Source:MGI Symbol;Acc:MGI:107285]	−0.768	3.681197e-02
Anxa3	annexin A3 [Source:MGI Symbol;Acc:MGI:1201378]	−0.772	4.275470e-02
Mtmr7	myotubularin related protein 7 [Source:MGI Symbol;Acc:MGI:1891693]	−0.773	1.679581e-02
Serinc3	serine incorporator 3 [Source:MGI Symbol;Acc:MGI:1349457]	−0.776	2.801900e-02
Kcnk1	potassium channel, subfamily K, member 1 [Source:MGI Symbol;Acc:MGI:109322]	−0.779	2.250656e-02
Bin1	bridging integrator 1 [Source:MGI Symbol;Acc:MGI:108092]	−0.779	9.653846e-03
Rab3ip	RAB3A interacting protein [Source:MGI Symbol;Acc:MGI:105933]	−0.782	1.653938e-02

Significant Genes

Runx1 and Runx2 Knock-out

Gene Name	Description	log2 FC	Adj p-value
Tns2	tensin 2 [Source:MGI Symbol;Acc:MGI:2387586]	−0.782	5.755382e-03
Mfap3l	microfibrillar-associated protein 3-like [Source:MGI Symbol;Acc:MGI:1918556]	−0.784	1.124934e-02
**Nabp1**	**nucleic acid binding protein 1 [Source:MGI Symbol;Acc:MGI:1923258]**	**−0.786**	**2.701496e-02**
Arhgap27	Rho GTPase activating protein 27 [Source:MGI Symbol;Acc:MGI:1916903]	−0.792	2.091232e-02
Uaca	uveal autoantigen with coiled-coil domains and ankyrin repeats [Source:MGI Symbol;Acc:MGI:1919815]	−0.793	7.556352e-03
Vmac	vimentin-type intermediate filament associated coiled-coil protein [Source:MGI Symbol;Acc:MGI:2146912]	−0.797	1.759646e-03
Cavin2	caveolae associated 2 [Source:MGI Symbol;Acc:MGI:99513]	−0.798	3.354740e-02
Glis3	GLIS family zinc finger 3 [Source:MGI Symbol;Acc:MGI:2444289]	−0.799	2.578215e-03
Sdr42e1	short chain dehydrogenase/reductase family 42E, member 1 [Source:MGI Symbol;Acc:MGI:1921282]	−0.800	1.910186e-02
Stk39	serine/threonine kinase 39 [Source:MGI Symbol;Acc:MGI:1858416]	−0.802	1.068606e-03
Gm37163	predicted gene, 37163 [Source:MGI Symbol;Acc:MGI:5610391]	−0.805	2.862322e-02
Txnip	thioredoxin interacting protein [Source:MGI Symbol;Acc:MGI:1889549]	−0.813	3.367085e-02

Significant Genes

Runx1 and Runx2 Knock-out

Gene Name	Description	log2 FC	Adj p-value
Gm47694	predicted gene, 47694 [Source:MGI Symbol;Acc:MGI:6096803]	−0.814	1.514398e-02
Rnf130	ring finger protein 130 [Source:MGI Symbol;Acc:MGI:1891717]	−0.814	4.284202e-05
Pdzrn3	PDZ domain containing RING finger 3 [Source:MGI Symbol;Acc:MGI:1933157]	−0.817	6.441589e-03
Trim68	tripartite motif-containing 68 [Source:MGI Symbol;Acc:MGI:2142077]	−0.818	9.208819e-03
D2hgdh	D-2-hydroxyglutarate dehydrogenase [Source:MGI Symbol;Acc:MGI:2138209]	−0.822	8.321805e-03
Car2	carbonic anhydrase 2 [Source:MGI Symbol;Acc:MGI:88269]	−0.822	4.770931e-02
Rhcg	Rhesus blood group-associated C glycoprotein [Source:MGI Symbol;Acc:MGI:1888517]	−0.823	4.539985e-02
Il17re	interleukin 17 receptor E [Source:MGI Symbol;Acc:MGI:1889371]	−0.827	1.031223e-02
Chchd7	coiled-coil-helix-coiled-coil-helix domain containing 7 [Source:MGI Symbol;Acc:MGI:1913683]	−0.829	4.843596e-03
Bid	BH3 interacting domain death agonist [Source:MGI Symbol;Acc:MGI:108093]	−0.834	5.595748e-03
Mtus1	mitochondrial tumor suppressor 1 [Source:MGI Symbol;Acc:MGI:2142572]	−0.845	2.704903e-04
Arrdc1	arrestin domain containing 1 [Source:MGI Symbol;Acc:MGI:2446136]	−0.846	1.523284e-02
Pck2	phosphoenolpyruvate carboxykinase 2 (mitochondrial) [Source:MGI	−0.846	1.344409e-02

Significant Genes

Runx1 and Runx2 Knock-out

Gene Name	Description	log2 FC	Adj p-value
	Symbol;Acc:MGI:1860456]		
Pik3c2b	phosphatidylinositol-4-phosphate 3-kinase catalytic subunit type 2 beta [Source:MGI Symbol;Acc:MGI:2685045]	−0.847	2.070901e-03
Foxn1	forkhead box N1 [Source:MGI Symbol;Acc:MGI:102949]	−0.848	3.792967e-02
Scrn2	secernin 2 [Source:MGI Symbol;Acc:MGI:1343092]	−0.849	3.295368e-02
Met	met proto-oncogene [Source:MGI Symbol;Acc:MGI:96969]	−0.852	1.655628e-02
Dipk1a	divergent protein kinase domain 1A [Source:MGI Symbol;Acc:MGI:1914516]	−0.853	2.544972e-02
Thap6	THAP domain containing 6 [Source:MGI Symbol;Acc:MGI:1922436]	−0.860	4.055789e-04
Sh3bgrl2	SH3 domain binding glutamic acid-rich protein like 2 [Source:MGI Symbol;Acc:MGI:1915350]	−0.864	7.469578e-04
Tmem106c	transmembrane protein 106C [Source:MGI Symbol;Acc:MGI:1196384]	−0.865	5.972308e-03
Ogfrl1	opioid growth factor receptor-like 1 [Source:MGI Symbol;Acc:MGI:1917405]	−0.871	3.072177e-04
Fam49a	family with sequence similarity 49, member A [Source:MGI Symbol;Acc:MGI:1261783]	−0.875	1.673194e-02
P3h4	prolyl 3-hydroxylase family member 4 (non-enzymatic) [Source:MGI Symbol;Acc:MGI:1913430]	−0.877	2.024245e-02
Svip	small VCP/p97-interacting protein [Source:MGI Symbol;Acc:MGI:1922994]	−0.884	8.979764e-03

Significant Genes

Runx1 and Runx2 Knock-out

Gene Name	Description	log2 FC	Adj p-value
Gpr157	G protein-coupled receptor 157 [Source:MGI Symbol;Acc:MGI:2442046]	−0.887	2.369302e-02
Gm16576	predicted gene 16576 [Source:MGI Symbol;Acc:MGI:4414996]	−0.888	3.479240e-02
Ptdss2	phosphatidylserine synthase 2 [Source:MGI Symbol;Acc:MGI:1351664]	−0.891	1.232443e-05
Tspan1	tetraspanin 1 [Source:MGI Symbol;Acc:MGI:1914055]	−0.892	4.691692e-02
Slc28a3	solute carrier family 28 (sodium-coupled nucleoside transporter), member 3 [Source:MGI Symbol;Acc:MGI:2137361]	−0.895	3.916736e-02
Bglap3	bone gamma-carboxyglutamate protein 3 [Source:MGI Symbol;Acc:MGI:88155]	−0.896	2.661233e-02
Dtx4	deltex 4, E3 ubiquitin ligase [Source:MGI Symbol;Acc:MGI:2672905]	−0.896	1.057688e-02
Pon3	paraoxonase 3 [Source:MGI Symbol;Acc:MGI:106686]	−0.897	3.593805e-02
Amot	angiomotin [Source:MGI Symbol;Acc:MGI:108440]	−0.899	4.377215e-03
Prkaa2	protein kinase, AMP-activated, alpha 2 catalytic subunit [Source:MGI Symbol;Acc:MGI:1336173]	−0.903	1.759360e-02
Cblc	Casitas B-lineage lymphoma c [Source:MGI Symbol;Acc:MGI:1931457]	−0.905	1.558587e-06
Sidt1	SID1 transmembrane family, member 1 [Source:MGI Symbol;Acc:MGI:2443155]	−0.908	3.248234e-02
Map1s	microtubule-associated protein 1S [Source:MGI Symbol;Acc:MGI:2443304]	−0.912	2.244630e-03

Significant Genes

Runx1 and Runx2 Knock-out

Gene Name	Description	log2 FC	Adj p-value
Shroom1	shroom family member 1 [Source:MGI Symbol;Acc:MGI:1919024]	−0.916	8.738395e-03
Proser2	proline and serine rich 2 [Source:MGI Symbol;Acc:MGI:2442238]	−0.917	2.472283e-03
Adamts15	a disintegrin-like and metallopeptidase (reprolysin type) with thrombospondin type 1 motif, 15 [Source:MGI Symbol;Acc:MGI:2449569]	−0.920	1.248539e-04
Bend7	BEN domain containing 7 [Source:MGI Symbol;Acc:MGI:2443100]	−0.920	1.691460e-02
Ecm1	extracellular matrix protein 1 [Source:MGI Symbol;Acc:MGI:103060]	−0.921	3.368549e-03
Gm10575	predicted gene 10575 [Source:MGI Symbol;Acc:MGI:3708765]	−0.921	1.228241e-02
Cldn23	claudin 23 [Source:MGI Symbol;Acc:MGI:1919158]	−0.925	3.443284e-02
Hid1	HID1 domain containing [Source:MGI Symbol;Acc:MGI:2445087]	−0.931	4.626142e-02
Rab4a	RAB4A, member RAS oncogene family [Source:MGI Symbol;Acc:MGI:105069]	−0.935	5.059065e-03
Peak1	pseudopodium-enriched atypical kinase 1 [Source:MGI Symbol;Acc:MGI:2442366]	−0.936	2.621610e-07
Tmem62	transmembrane protein 62 [Source:MGI Symbol;Acc:MGI:2139461]	−0.936	1.098558e-03
Manba	mannosidase, beta A, lysosomal [Source:MGI Symbol;Acc:MGI:88175]	−0.937	1.513415e-02
Grtp1	GH regulated TBC protein 1 [Source:MGI Symbol;Acc:MGI:1914040]	−0.938	2.852973e-05

Significant Genes

Runx1 and Runx2 Knock-out

Gene Name	Description	log2 FC	Adj p-value
Bmp6	bone morphogenetic protein 6 [Source:MGI Symbol;Acc:MGI:88182]	−0.940	7.882703e-03
Dapp1	dual adaptor for phosphotyrosine and 3-phosphoinositides 1 [Source:MGI Symbol;Acc:MGI:1347063]	−0.943	4.549240e-03
Pacs1	phosphofurin acidic cluster sorting protein 1 [Source:MGI Symbol;Acc:MGI:1277113]	−0.943	4.792976e-07
Pla2g2e	phospholipase A2, group IIE [Source:MGI Symbol;Acc:MGI:1349660]	−0.944	4.397184e-02
Paqr6	progestin and adipoQ receptor family member VI [Source:MGI Symbol;Acc:MGI:1916207]	−0.945	3.416060e-02
Appl2	adaptor protein, phosphotyrosine interaction, PH domain and leucine zipper containing 2 [Source:MGI Symbol;Acc:MGI:2384914]	−0.948	5.488144e-05
Pgpep1	pyroglutamyl-peptidase I [Source:MGI Symbol;Acc:MGI:1913772]	−0.949	2.618639e-05
Rundc3a	RUN domain containing 3A [Source:MGI Symbol;Acc:MGI:1858752]	−0.949	1.351835e-02
Lgals2	lectin, galactose-binding, soluble 2 [Source:MGI Symbol;Acc:MGI:895068]	−0.950	2.447459e-02
C130021I20Rik	Riken cDNA C130021I20 gene [Source:MGI Symbol;Acc:MGI:3639863]	−0.951	1.131869e-02
Optn	optineurin [Source:MGI Symbol;Acc:MGI:1918898]	−0.951	9.887075e-03
Hgsnat	heparan-alpha-glucosaminide N-acetyltransferase [Source:MGI Symbol;Acc:MGI:1196297]	−0.952	1.954124e-05

Significant Genes<br>Runx1 and Runx2 Knock-out			
Gene Name	Description	log2 FC	Adj p-value
Snta1	syntrophin, acidic 1 [Source:MGI Symbol;Acc:MGI:101772]	−0.953	3.828109e-04
Ikzf2	IKAROS family zinc finger 2 [Source:MGI Symbol;Acc:MGI:1342541]	−0.954	2.490771e-02
Zfp990	zinc finger protein 990 [Source:MGI Symbol;Acc:MGI:3652161]	−0.957	1.973904e-02
Mgat4a	mannoside acetylglucosaminyltransferase 4, isoenzyme A [Source:MGI Symbol;Acc:MGI:2662992]	−0.960	4.371984e-05
Arrb1	arrestin, beta 1 [Source:MGI Symbol;Acc:MGI:99473]	−0.962	2.644135e-04
Usp43	ubiquitin specific peptidase 43 [Source:MGI Symbol;Acc:MGI:2444541]	−0.965	7.331618e-03
Ly6e	lymphocyte antigen 6 complex, locus E [Source:MGI Symbol;Acc:MGI:106651]	−0.967	2.079544e-02
Fgfrl1	fibroblast growth factor receptor-like 1 [Source:MGI Symbol;Acc:MGI:2150920]	−0.968	1.056774e-03
Sox6	SRY (sex determining region Y)-box 6 [Source:MGI Symbol;Acc:MGI:98368]	−0.973	1.780484e-02
Pamr1	peptidase domain containing associated with muscle regeneration 1 [Source:MGI Symbol;Acc:MGI:2445082]	−0.977	4.887622e-02
Zfp469	zinc finger protein 469 [Source:MGI Symbol;Acc:MGI:2684868]	−0.979	6.066780e-03
Ikzf1	IKAROS family zinc finger 1 [Source:MGI Symbol;Acc:MGI:1342540]	−0.982	4.690495e-02
Dock8	dedicator of cytokinesis 8 [Source:MGI Symbol;Acc:MGI:1921396]	−0.984	2.339612e-10

Significant Genes

Runx1 and Runx2 Knock-out

Gene Name	Description	log2 FC	Adj p-value
Stat5b	signal transducer and activator of transcription 5B [Source:MGI Symbol;Acc:MGI:103035]	−0.985	8.394092e-05
Rnf180	ring finger protein 180 [Source:MGI Symbol;Acc:MGI:1919066]	−0.986	8.769699e-03
Igfals	insulin-like growth factor binding protein, acid labile subunit [Source:MGI Symbol;Acc:MGI:107973]	−0.986	1.020571e-02
Orai3	ORAI calcium release-activated calcium modulator 3 [Source:MGI Symbol;Acc:MGI:3039586]	−0.987	2.384682e-03
Itpr1	inositol 1,4,5-trisphosphate receptor 1 [Source:MGI Symbol;Acc:MGI:96623]	−0.989	5.308099e-11
Col6a3	collagen, type VI, alpha 3 [Source:MGI Symbol;Acc:MGI:88461]	−0.990	4.851128e-02
2200002D01Rik	RIKEN cDNA 2200002D01 gene [Source:MGI Symbol;Acc:MGI:1919525]	−0.996	1.478041e-03
Spink5	serine peptidase inhibitor, Kazal type 5 [Source:MGI Symbol;Acc:MGI:1919682]	−0.997	1.197665e-02
Zfp787	zinc finger protein 787 [Source:MGI Symbol;Acc:MGI:1914359]	−0.998	1.420258e-05
Fmn1	formin 1 [Source:MGI Symbol;Acc:MGI:101815]	−0.999	7.216574e-04
Irs1	insulin receptor substrate 1 [Source:MGI Symbol;Acc:MGI:99454]	−0.999	2.070716e-02
Entpd8	ectonucleoside triphosphate diphosphohydrolase 8 [Source:MGI Symbol;Acc:MGI:1919340]	−1.002	4.691692e-02

Significant Genes

Runx1 and Runx2 Knock-out

Gene Name	Description	log2 FC	Adj p-value
Erfe	erythroferrone [Source:MGI Symbol;Acc:MGI:3606476]	−1.003	2.104732e-04
Them4	thioesterase superfamily member 4 [Source:MGI Symbol;Acc:MGI:1923028]	−1.007	1.089522e-02
Fgg	fibrinogen gamma chain [Source:MGI Symbol;Acc:MGI:95526]	−1.010	4.899793e-02
Rnaset2a	ribonuclease T2A [Source:MGI Symbol;Acc:MGI:1915445]	−1.013	3.295483e-02
Phactr1	phosphatase and actin regulator 1 [Source:MGI Symbol;Acc:MGI:2659021]	−1.013	3.783306e-02
Nbea	neurobeachin [Source:MGI Symbol;Acc:MGI:1347075]	−1.014	8.283603e-06
Proca1	protein interacting with cyclin A1 [Source:MGI Symbol;Acc:MGI:1918274]	−1.017	1.284175e-02
Ccdc162	coiled-coil domain containing 162 [Source:MGI Symbol;Acc:MGI:1923223]	−1.017	4.785048e-02
Marchf8	membrane associated ring-CH-type finger 8 [Source:MGI Symbol;Acc:MGI:1919029]	−1.020	3.293994e-09
Grk3	G protein-coupled receptor kinase 3 [Source:MGI Symbol;Acc:MGI:87941]	−1.020	1.575640e-02
P2ry2	purinergic receptor P2Y, G-protein coupled 2 [Source:MGI Symbol;Acc:MGI:105107]	−1.021	2.004778e-02
Fcgbp	Fc fragment of IgG binding protein [Source:MGI Symbol;Acc:MGI:2444336]	−1.022	4.642193e-02
Pacsin1	protein kinase C and casein kinase substrate in neurons 1 [Source:MGI Symbol;Acc:MGI:1345181]	−1.022	4.241583e-02

Significant Genes

Runx1 and Runx2 Knock-out

Gene Name	Description	log2 FC	Adj p-value
Prkca	protein kinase C, alpha [Source:MGI Symbol;Acc:MGI:97595]	−1.023	6.236086e-07
Prxl2c	peroxiredoxin like 2C [Source:MGI Symbol;Acc:MGI:1913379]	−1.024	8.374758e-06
Lgals12	lectin, galactose binding, soluble 12 [Source:MGI Symbol;Acc:MGI:1929094]	−1.025	2.176523e-02
Gfra2	glial cell line derived neurotrophic factor family receptor alpha 2 [Source:MGI Symbol;Acc:MGI:1195462]	−1.026	4.488111e-02
Lrrc15	leucine rich repeat containing 15 [Source:MGI Symbol;Acc:MGI:1921738]	−1.027	3.684216e-02
Krt39	keratin 39 [Source:MGI Symbol;Acc:MGI:3588208]	−1.033	3.068760e-02
Cryl1	crystallin, lambda 1 [Source:MGI Symbol;Acc:MGI:1915881]	−1.033	9.354956e-04
Mtss2	MTSS I-BAR domain containing 2 [Source:MGI Symbol;Acc:MGI:3039591]	−1.037	4.676993e-03
Chadl	chondroadherin-like [Source:MGI Symbol;Acc:MGI:3036284]	−1.042	1.302727e-02
Slc35f2	solute carrier family 35, member F2 [Source:MGI Symbol;Acc:MGI:1919272]	−1.043	1.035903e-02
Nupr1	nuclear protein transcription regulator 1 [Source:MGI Symbol;Acc:MGI:1891834]	−1.046	9.943744e-03
Hivep3	human immunodeficiency virus type I enhancer binding protein 3 [Source:MGI Symbol;Acc:MGI:106589]	−1.048	1.436589e-05
Abcc3	ATP-binding cassette, sub-family C (CFTR/MRP), member 3 [Source:MGI	−1.048	2.004778e-02

Significant Genes

Runx1 and Runx2 Knock-out

Gene Name	Description	log2 FC	Adj p-value
	Symbol;Acc:MGI:1923658]		
Marveld3	MARVEL (membrane-associating) domain containing 3 [Source:MGI Symbol;Acc:MGI:1920858]	−1.052	1.154005e-05
S100a3	S100 calcium binding protein A3 [Source:MGI Symbol;Acc:MGI:1338849]	−1.052	3.632062e-02
Rnf157	ring finger protein 157 [Source:MGI Symbol;Acc:MGI:2442484]	−1.053	3.408195e-02
Greb1l	growth regulation by estrogen in breast cancer-like [Source:MGI Symbol;Acc:MGI:3576497]	−1.054	2.178237e-02
Cd55	CD55 molecule, decay accelerating factor for complement [Source:MGI Symbol;Acc:MGI:104850]	−1.061	8.914164e-04
Pik3cg	phosphatidylinositol-4,5-bisphosphate 3-kinase catalytic subunit gamma [Source:MGI Symbol;Acc:MGI:1353576]	−1.061	4.025555e-02
Nes	nestin [Source:MGI Symbol;Acc:MGI:101784]	−1.062	1.873001e-03
Gm42528	predicted gene 42528 [Source:MGI Symbol;Acc:MGI:5662665]	−1.063	3.197334e-02
Gpd1	glycerol-3-phosphate dehydrogenase 1 (soluble) [Source:MGI Symbol;Acc:MGI:95679]	−1.064	7.465353e-03
Gm45267	predicted gene 45267 [Source:MGI Symbol;Acc:MGI:5791103]	−1.065	2.164748e-02
Rasgrf1	RAS protein-specific guanine nucleotide-releasing factor 1 [Source:MGI Symbol;Acc:MGI:99694]	−1.065	3.967355e-02

Significant Genes

Runx1 and Runx2 Knock-out

Gene Name	Description	log2 FC	Adj p-value
Capn8	calpain 8 [Source:MGI Symbol;Acc:MGI:2181366]	−1.065	4.013467e-02
Lpin3	lipin 3 [Source:MGI Symbol;Acc:MGI:1891342]	−1.065	1.116444e-05
Fads6	fatty acid desaturase domain family, member 6 [Source:MGI Symbol;Acc:MGI:3039592]	−1.068	3.925372e-02
Rap1gap	Rap1 GTPase-activating protein [Source:MGI Symbol;Acc:MGI:109338]	−1.068	8.818120e-03
Kifc3	kinesin family member C3 [Source:MGI Symbol;Acc:MGI:109202]	−1.072	7.141753e-03
Samd10	sterile alpha motif domain containing 10 [Source:MGI Symbol;Acc:MGI:2443872]	−1.072	3.938834e-04
Gm35853	predicted gene, 35853 [Source:MGI Symbol;Acc:MGI:5595012]	−1.072	2.634947e-02
Gm16351	predicted gene 16351 [Source:MGI Symbol;Acc:MGI:3840153]	−1.072	3.883349e-02
Ccdc141	coiled-coil domain containing 141 [Source:MGI Symbol;Acc:MGI:1919735]	−1.072	7.992631e-05
Asrgl1	asparaginase like 1 [Source:MGI Symbol;Acc:MGI:1913764]	−1.073	6.709838e-04
Scarf2	scavenger receptor class F, member 2 [Source:MGI Symbol;Acc:MGI:1858430]	−1.073	6.339128e-03
Pde4d	phosphodiesterase 4D, cAMP specific [Source:MGI Symbol;Acc:MGI:99555]	−1.075	7.250851e-06
Rab11fip1	RAB11 family interacting protein 1 (class I) [Source:MGI Symbol;Acc:MGI:1923017]	−1.081	8.076122e-03
Gm38910	predicted gene, 38910 [Source:MGI Symbol;Acc:MGI:5621795]	−1.082	3.825392e-02

Significant Genes

Runx1 and Runx2 Knock-out

Gene Name	Description	log2 FC	Adj p-value
Lpcat1	lysophosphatidylcholine acyltransferase 1 [Source:MGI Symbol;Acc:MGI:2384812]	−1.084	6.678335e-07
Unc13b	unc-13 homolog B [Source:MGI Symbol;Acc:MGI:1342278]	−1.084	4.695374e-04
C1qtnf1	C1q and tumor necrosis factor related protein 1 [Source:MGI Symbol;Acc:MGI:1919254]	−1.085	2.912910e-03
Marchf3	membrane associated ring-CH-type finger 3 [Source:MGI Symbol;Acc:MGI:2443667]	−1.086	2.339785e-03
Clic5	chloride intracellular channel 5 [Source:MGI Symbol;Acc:MGI:1917912]	−1.090	2.471616e-02
Tspan12	tetraspanin 12 [Source:MGI Symbol;Acc:MGI:1889818]	−1.093	1.319644e-04
Fam20a	family with sequence similarity 20, member A [Source:MGI Symbol;Acc:MGI:2388266]	−1.096	1.126417e-03
AU018091	expressed sequence AU018091 [Source:MGI Symbol;Acc:MGI:2142124]	−1.096	2.303501e-02
Il18r1	interleukin 18 receptor 1 [Source:MGI Symbol;Acc:MGI:105383]	−1.101	8.732706e-03
Magi3	membrane associated guanylate kinase, WW and PDZ domain containing 3 [Source:MGI Symbol;Acc:MGI:1923484]	−1.102	1.043798e-05
Gm44260	predicted gene, 44260 [Source:MGI Symbol;Acc:MGI:5690652]	−1.104	1.151768e-03
Tspan7	tetraspanin 7 [Source:MGI Symbol;Acc:MGI:1298407]	−1.107	2.268868e-08
Pdlim2	PDZ and LIM domain 2 [Source:MGI Symbol;Acc:MGI:2384850]	−1.109	7.367189e-04

Significant Genes

Runx1 and Runx2 Knock-out

Gene Name	Description	log2 FC	Adj p-value
Ebf4	early B cell factor 4 [Source:MGI Symbol;Acc:MGI:2385972]	−1.111	3.295483e-02
Kcnj12	potassium inwardly-rectifying channel, subfamily J, member 12 [Source:MGI Symbol;Acc:MGI:108495]	−1.111	2.160127e-02
Ppp1r1b	protein phosphatase 1, regulatory inhibitor subunit 1B [Source:MGI Symbol;Acc:MGI:94860]	−1.113	3.153252e-02
Myh14	myosin, heavy polypeptide 14 [Source:MGI Symbol;Acc:MGI:1919210]	−1.114	1.454443e-02
Timp2	tissue inhibitor of metalloproteinase 2 [Source:MGI Symbol;Acc:MGI:98753]	−1.117	1.837451e-04
C530043A13Rik	RIKEN cDNA C530043A13 gene [Source:MGI Symbol;Acc:MGI:2441914]	−1.118	3.448910e-02
G930009F23Rik	RIKEN cDNA G930009F23 gene [Source:MGI Symbol;Acc:MGI:3642701]	−1.119	6.378841e-03
Wnt16	wingless-type MMTV integration site family, member 16 [Source:MGI Symbol;Acc:MGI:2136018]	−1.122	4.392490e-03
Plb1	phospholipase B1 [Source:MGI Symbol;Acc:MGI:1922406]	−1.124	3.416292e-02
Cd82	CD82 antigen [Source:MGI Symbol;Acc:MGI:104651]	−1.124	8.278264e-03
Pld2	phospholipase D2 [Source:MGI Symbol;Acc:MGI:892877]	−1.125	1.080850e-03
Ermp1	endoplasmic reticulum metallopeptidase 1 [Source:MGI Symbol;Acc:MGI:106250]	−1.127	1.665384e-04

Significant Genes

Runx1 and Runx2 Knock-out

Gene Name	Description	log2 FC	Adj p-value
**Gprc5a**	**G protein-coupled receptor, family C, group 5, member A [Source:MGI Symbol;Acc:MGI:1891250]**	**−1.135**	**5.188626e-03**
Nav3	neuron navigator 3 [Source:MGI Symbol;Acc:MGI:2183703]	−1.136	1.049459e-02
Bbox1	butyrobetaine (gamma), 2-oxoglutarate dioxygenase 1 (gamma-butyrobetaine hydroxylase) [Source:MGI Symbol;Acc:MGI:1891372]	−1.139	2.411033e-02
Pald1	phosphatase domain containing, paladin 1 [Source:MGI Symbol;Acc:MGI:1351623]	−1.140	1.853220e-05
Gpr68	G protein-coupled receptor 68 [Source:MGI Symbol;Acc:MGI:2441763]	−1.141	8.016177e-03
AI854703	expressed sequence AI854703 [Source:MGI Symbol;Acc:MGI:2141510]	−1.147	2.639590e-02
Bicdl2	BICD family like cargo adaptor 2 [Source:MGI Symbol;Acc:MGI:2388267]	−1.150	5.410777e-03
Agtr1a	angiotensin II receptor, type 1a [Source:MGI Symbol;Acc:MGI:87964]	−1.152	2.810435e-02
Slc29a1	solute carrier family 29 (nucleoside transporters), member 1 [Source:MGI Symbol;Acc:MGI:1927073]	−1.153	4.873416e-05
Tln2	talin 2 [Source:MGI Symbol;Acc:MGI:1917799]	−1.154	6.236086e-07
Crat	carnitine acetyltransferase [Source:MGI Symbol;Acc:MGI:109501]	−1.154	4.779655e-04
Npas3	neuronal PAS domain protein 3 [Source:MGI Symbol;Acc:MGI:1351610]	−1.156	2.627961e-02

Significant Genes

Runx1 and Runx2 Knock-out

Gene Name	Description	log2 FC	Adj p-value
Chrnb1	cholinergic receptor, nicotinic, beta polypeptide 1 (muscle) [Source:MGI Symbol;Acc:MGI:87890]	−1.160	1.947963e-03
**Cryab**	**crystallin, alpha B [Source:MGI Symbol;Acc:MGI:88516]**	**−1.163**	**8.013027e-03**
Nipal3	NIPA-like domain containing 3 [Source:MGI Symbol;Acc:MGI:1921802]	−1.163	4.024799e-09
Unc5cl	unc-5 family C-terminal like [Source:MGI Symbol;Acc:MGI:1923839]	−1.166	2.801900e-02
Dock9	dedicator of cytokinesis 9 [Source:MGI Symbol;Acc:MGI:106321]	−1.166	3.024860e-09
Caskin1	CASK interacting protein 1 [Source:MGI Symbol;Acc:MGI:2442952]	−1.167	2.415473e-02
Acot7	acyl-CoA thioesterase 7 [Source:MGI Symbol;Acc:MGI:1917275]	−1.168	6.866083e-03
Il1r1	interleukin 1 receptor, type I [Source:MGI Symbol;Acc:MGI:96545]	−1.174	2.023499e-15
Pkdcc	protein kinase domain containing, cytoplasmic [Source:MGI Symbol;Acc:MGI:2147077]	−1.176	1.072544e-02
Krtap11-1	keratin associated protein 11-1 [Source:MGI Symbol;Acc:MGI:99447]	−1.179	2.862322e-02
Gna14	guanine nucleotide binding protein, alpha 14 [Source:MGI Symbol;Acc:MGI:95769]	−1.180	7.882703e-03
Gucy1a2	guanylate cyclase 1, soluble, alpha 2 [Source:MGI Symbol;Acc:MGI:2660877]	−1.180	2.287148e-02
Rnase1	ribonuclease, RNase A family, 1 (pancreatic) [Source:MGI Symbol;Acc:MGI:97919]	−1.181	2.822532e-02

Significant Genes

Runx1 and Runx2 Knock-out

Gene Name	Description	log2 FC	Adj p-value
Tgm6	transglutaminase 6 [Source:MGI Symbol;Acc:MGI:3044321]	−1.182	1.509127e-02
Padi1	peptidyl arginine deiminase, type I [Source:MGI Symbol;Acc:MGI:1338893]	−1.186	1.295212e-02
Prrt1	proline-rich transmembrane protein 1 [Source:MGI Symbol;Acc:MGI:1932118]	−1.187	1.863563e-03
Hapln3	hyaluronan and proteoglycan link protein 3 [Source:MGI Symbol;Acc:MGI:1914916]	−1.191	2.769228e-02
Wipi1	WD repeat domain, phosphoinositide interacting 1 [Source:MGI Symbol;Acc:MGI:1261864]	−1.191	6.315247e-07
Heyl	hairy/enhancer-of-split related with YRPW motif-like [Source:MGI Symbol;Acc:MGI:1860511]	−1.193	1.317288e-03
Mgat3	mannoside acetylglucosaminyltransferase 3 [Source:MGI Symbol;Acc:MGI:104532]	−1.194	2.124864e-02
Csn3	casein kappa [Source:MGI Symbol;Acc:MGI:107461]	−1.201	7.672566e-03
Cacna1i	calcium channel, voltage-dependent, alpha 1I subunit [Source:MGI Symbol;Acc:MGI:2178051]	−1.206	8.400721e-03
Cited1	Cbp/p300-interacting transactivator with Glu/Asp-rich carboxy-terminal domain 1 [Source:MGI Symbol;Acc:MGI:108023]	−1.214	2.145099e-02
Rilp	Rab interacting lysosomal protein [Source:MGI Symbol;Acc:MGI:2144271]	−1.214	2.514473e-02
Gm6420	predicted gene 6420 [Source:MGI Symbol;Acc:MGI:3644588]	−1.215	9.904012e-04

Significant Genes

Runx1 and Runx2 Knock-out

Gene Name	Description	log2 FC	Adj p-value
Zfp395	zinc finger protein 395 [Source:MGI Symbol;Acc:MGI:2682318]	−1.216	4.235479e-04
Shpk	sedoheptulokinase [Source:MGI Symbol;Acc:MGI:1921887]	−1.216	1.823636e-02
Ldlrad4	low density lipoprotein receptor class A domain containing 4 [Source:MGI Symbol;Acc:MGI:1277150]	−1.217	3.054455e-04
Tspan15	tetraspanin 15 [Source:MGI Symbol;Acc:MGI:1917673]	−1.217	1.984146e-02
Tph1	tryptophan hydroxylase 1 [Source:MGI Symbol;Acc:MGI:98796]	−1.218	2.367142e-02
Tead2	TEA domain family member 2 [Source:MGI Symbol;Acc:MGI:104904]	−1.223	1.153193e-07
Slc16a12	solute carrier family 16 (monocarboxylic acid transporters), member 12 [Source:MGI Symbol;Acc:MGI:2147716]	−1.225	4.517961e-03
Fer1l4	fer-1-like 4 (C. elegans) [Source:MGI Symbol;Acc:MGI:1921812]	−1.226	2.495529e-02
D7Ertd443e	DNA segment, Chr 7, ERATO Doi 443, expressed [Source:MGI Symbol;Acc:MGI:1196431]	−1.230	1.423084e-02
**Cbx7**	**chromobox 7 [Source:MGI Symbol;Acc:MGI:1196439]**	**−1.230**	**1.681694e-05**
Gabrg3	gamma-aminobutyric acid (GABA) A receptor, subunit gamma 3 [Source:MGI Symbol;Acc:MGI:95624]	−1.235	2.373109e-02
Tmem184a	transmembrane protein 184a [Source:MGI Symbol;Acc:MGI:2385897]	−1.237	2.388126e-05

Significant Genes

Runx1 and Runx2 Knock-out

Gene Name	Description	log2 FC	Adj p-value
Cdsn	corneodesmosin [Source:MGI Symbol;Acc:MGI:3505689]	−1.237	1.126145e-03
Plekhb1	pleckstrin homology domain containing, family B (evectins) member 1 [Source:MGI Symbol;Acc:MGI:1351469]	−1.240	5.323622e-05
Gm19461	predicted gene, 19461 [Source:MGI Symbol;Acc:MGI:5011646]	−1.241	2.374257e-02
**Map2**	**microtubule-associated protein 2 [Source:MGI Symbol;Acc:MGI:97175]**	**−1.245**	**6.308302e-05**
Lrg1	leucine-rich alpha-2-glycoprotein 1 [Source:MGI Symbol;Acc:MGI:1924155]	−1.246	3.259620e-03
Sec14l4	SEC14-like lipid binding 4 [Source:MGI Symbol;Acc:MGI:2144095]	−1.246	2.298874e-02
Chmp4c	charged multivesicular body protein 4C [Source:MGI Symbol;Acc:MGI:1913621]	−1.247	7.213857e-06
Fkbp5	FK506 binding protein 5 [Source:MGI Symbol;Acc:MGI:104670]	−1.254	6.448226e-07
Eci1	enoyl-Coenzyme A delta isomerase 1 [Source:MGI Symbol;Acc:MGI:94871]	−1.256	7.845017e-05
Acsbg1	acyl-CoA synthetase bubblegum family member 1 [Source:MGI Symbol;Acc:MGI:2385656]	−1.260	8.356341e-04
Igsf5	immunoglobulin superfamily, member 5 [Source:MGI Symbol;Acc:MGI:1919308]	−1.262	2.070855e-02
Megf6	multiple EGF-like-domains 6 [Source:MGI Symbol;Acc:MGI:1919351]	−1.263	2.087615e-02
Stc2	stanniocalcin 2 [Source:MGI Symbol;Acc:MGI:1316731]	−1.263	7.331618e-03

Significant Genes

Runx1 and Runx2 Knock-out

Gene Name	Description	log2 FC	Adj p-value
Tle2	transducin-like enhancer of split 2 [Source:MGI Symbol;Acc:MGI:104635]	−1.264	4.229535e-03
Pxylp1	2-phosphoxylose phosphatase 1 [Source:MGI Symbol;Acc:MGI:2442444]	−1.264	5.722560e-05
Arhgap44	Rho GTPase activating protein 44 [Source:MGI Symbol;Acc:MGI:2144423]	−1.267	7.216574e-04
B3gnt3	UDP-GlcNAc:betaGal beta-1,3-N-acetylglucosaminyltransferase 3 [Source:MGI Symbol;Acc:MGI:2152535]	−1.268	9.943744e-03
Slc40a1	solute carrier family 40 (iron-regulated transporter), member 1 [Source:MGI Symbol;Acc:MGI:1315204]	−1.269	1.115541e-03
Zfp775	zinc finger protein 775 [Source:MGI Symbol;Acc:MGI:2683557]	−1.272	3.892739e-03
1700086D15Rik	RIKEN cDNA 1700086D15 gene [Source:MGI Symbol;Acc:MGI:1921532]	−1.273	1.196641e-03
**Barx2**	**BarH-like homeobox 2 [Source:MGI Symbol;Acc:MGI:109617]**	**−1.274**	**2.135190e-03**
Galc	galactosylceramidase [Source:MGI Symbol;Acc:MGI:95636]	−1.274	9.078282e-08
B230118H07Rik	RIKEN cDNA B230118H07 gene [Source:MGI Symbol;Acc:MGI:1915420]	−1.276	6.238842e-06
Gask1b	golgi associated kinase 1B [Source:MGI Symbol;Acc:MGI:1915909]	−1.278	2.018604e-02
Gm44250	predicted gene, 44250 [Source:MGI Symbol;Acc:MGI:5690642]	−1.278	6.031714e-04
Slc45a3	solute carrier family 45, member 3 [Source:MGI Symbol;Acc:MGI:1922082]	−1.278	5.087704e-04

Significant Genes

Runx1 and Runx2 Knock-out

Gene Name	Description	log2 FC	Adj p-value
Nfatc2	nuclear factor of activated T cells, cytoplasmic, calcineurin dependent 2 [Source:MGI Symbol;Acc:MGI:102463]	−1.279	1.027218e-03
Cpq	carboxypeptidase Q [Source:MGI Symbol;Acc:MGI:1889205]	−1.280	1.656728e-02
Misp	mitotic spindle positioning [Source:MGI Symbol;Acc:MGI:1926156]	−1.281	1.626438e-02
Gab2	growth factor receptor bound protein 2-associated protein 2 [Source:MGI Symbol;Acc:MGI:1333854]	−1.282	4.442858e-03
Rasgef1c	RasGEF domain family, member 1C [Source:MGI Symbol;Acc:MGI:1921813]	−1.284	1.253863e-02
Erbb4	erb-b2 receptor tyrosine kinase 4 [Source:MGI Symbol;Acc:MGI:104771]	−1.286	2.017454e-02
Hmgcs2	3-hydroxy-3-methylglutaryl-Coenzyme A synthase 2 [Source:MGI Symbol;Acc:MGI:101939]	−1.288	2.058017e-02
Stk10	serine/threonine kinase 10 [Source:MGI Symbol;Acc:MGI:1099439]	−1.291	5.164081e-05
**Krt80**	**keratin 80 [Source:MGI Symbol;Acc:MGI:1921377]**	**−1.295**	**1.927504e-03**
Ttpa	tocopherol (alpha) transfer protein [Source:MGI Symbol;Acc:MGI:1354168]	−1.296	7.527033e-04
Npas2	neuronal PAS domain protein 2 [Source:MGI Symbol;Acc:MGI:109232]	−1.297	7.536205e-05
Inhbb	inhibin beta-B [Source:MGI Symbol;Acc:MGI:96571]	−1.298	1.995722e-03

Significant Genes

Runx1 and Runx2 Knock-out

Gene Name	Description	log2 FC	Adj p-value
5330417C22Rik	RIKEN cDNA 5330417C22 gene [Source:MGI Symbol;Acc:MGI:1923930]	−1.301	1.989292e-02
Arhgap27os3	Rho GTPase activating protein 27, opposite strand 3 [Source:MGI Symbol;Acc:MGI:3650159]	−1.303	6.632290e-03
Abhd14b	abhydrolase domain containing 14b [Source:MGI Symbol;Acc:MGI:1923741]	−1.303	1.352894e-03
Pkn1	protein kinase N1 [Source:MGI Symbol;Acc:MGI:108022]	−1.305	1.331239e-04
Gm44956	predicted gene 44956 [Source:MGI Symbol;Acc:MGI:5753532]	−1.306	1.206469e-02
Plaat3	phospholipase A and acyltransferase 3 [Source:MGI Symbol;Acc:MGI:2179715]	−1.306	1.497947e-02
Dapk1	death associated protein kinase 1 [Source:MGI Symbol;Acc:MGI:1916885]	−1.306	5.454498e-07
Ncald	neurocalcin delta [Source:MGI Symbol;Acc:MGI:1196326]	−1.307	1.295212e-02
Tgfb3	transforming growth factor, beta 3 [Source:MGI Symbol;Acc:MGI:98727]	−1.311	8.394092e-05
Sox11	SRY (sex determining region Y)-box 11 [Source:MGI Symbol;Acc:MGI:98359]	−1.311	1.686160e-03
Ppm1h	protein phosphatase 1H (PP2C domain containing) [Source:MGI Symbol;Acc:MGI:2442087]	−1.312	1.246334e-03
Bhlhe41	basic helix-loop-helix family, member e41 [Source:MGI Symbol;Acc:MGI:1930704]	−1.315	8.793396e-05
Vegfc	vascular endothelial growth factor C [Source:MGI Symbol;Acc:MGI:109124]	−1.316	1.741096e-03

Significant Genes

Runx1 and Runx2 Knock-out

Gene Name	Description	log2 FC	Adj p-value
Crip1	cysteine-rich protein 1 (intestinal) [Source:MGI Symbol;Acc:MGI:88501]	−1.317	1.220215e-03
Gm13807	predicted gene 13807 [Source:MGI Symbol;Acc:MGI:3650023]	−1.319	1.811276e-04
Hs3st6	heparan sulfate (glucosamine) 3-O-sulfotransferase 6 [Source:MGI Symbol;Acc:MGI:3580487]	−1.324	1.206469e-02
Rnf125	ring finger protein 125 [Source:MGI Symbol;Acc:MGI:1914914]	−1.331	4.125159e-04
Gm42583	predicted gene 42583 [Source:MGI Symbol;Acc:MGI:5662720]	−1.335	1.339671e-02
Cmtm8	CKLF-like MARVEL transmembrane domain containing 8 [Source:MGI Symbol;Acc:MGI:2447167]	−1.336	3.075851e-03
Reps2	RALBP1 associated Eps domain containing protein 2 [Source:MGI Symbol;Acc:MGI:2663511]	−1.340	4.565899e-03
**Itih5**	**inter-alpha (globulin) inhibitor H5 [Source:MGI Symbol;Acc:MGI:1925751]**	**−1.340**	**4.294100e-05**
Plekhh1	pleckstrin homology domain containing, family H (with MyTH4 domain) member 1 [Source:MGI Symbol;Acc:MGI:2144989]	−1.342	2.400180e-05
Slc25a23	solute carrier family 25 (mitochondrial carrier; phosphate carrier), member 23 [Source:MGI Symbol;Acc:MGI:1914222]	−1.343	1.514759e-03
Rims2	regulating synaptic membrane exocytosis 2 [Source:MGI Symbol;Acc:MGI:2152972]	−1.344	1.521876e-02

Significant Genes

Runx1 and Runx2 Knock-out

Gene Name	Description	log2 FC	Adj p-value
Rab17	RAB17, member RAS oncogene family [Source:MGI Symbol;Acc:MGI:104640]	−1.348	6.393484e-03
**Apobec3**	**apolipoprotein B mRNA editing enzyme, catalytic polypeptide 3 [Source:MGI Symbol;Acc:MGI:1933111]**	**−1.352**	**1.436427e-03**
Pdk2	pyruvate dehydrogenase kinase, isoenzyme 2 [Source:MGI Symbol;Acc:MGI:1343087]	−1.353	5.070242e-03
Smpd1	sphingomyelin phosphodiesterase 1, acid lysosomal [Source:MGI Symbol;Acc:MGI:98325]	−1.354	1.314469e-03
Krt4	keratin 4 [Source:MGI Symbol;Acc:MGI:96701]	−1.356	6.228468e-03
Gm38157	predicted gene, 38157 [Source:MGI Symbol;Acc:MGI:5611385]	−1.360	1.995722e-03
Spns3	spinster homolog 3 [Source:MGI Symbol;Acc:MGI:1924827]	−1.360	1.605800e-02
Zbtb7c	zinc finger and BTB domain containing 7C [Source:MGI Symbol;Acc:MGI:2443302]	−1.361	4.125159e-04
Cpeb3	cytoplasmic polyadenylation element binding protein 3 [Source:MGI Symbol;Acc:MGI:2443075]	−1.362	4.513753e-06
Hes2	hes family bHLH transcription factor 2 [Source:MGI Symbol;Acc:MGI:1098624]	−1.362	7.372002e-03
Arhgap8	Rho GTPase activating protein 8 [Source:MGI Symbol;Acc:MGI:1920417]	−1.363	4.473591e-04
Parvb	parvin, beta [Source:MGI Symbol;Acc:MGI:2153063]	−1.363	4.513753e-06
Gdpd1	glycerophosphodiester phosphodiesterase domain containing 1 [Source:MGI	−1.364	4.701300e-05

Significant Genes

Runx1 and Runx2 Knock-out

Gene Name	Description	log2 FC	Adj p-value
	Symbol;Acc:MGI:1913819]		
Krt82	keratin 82 [Source:MGI Symbol;Acc:MGI:2149248]	−1.373	1.004647e-02
Gprc5c	G protein-coupled receptor, family C, group 5, member C [Source:MGI Symbol;Acc:MGI:1917605]	−1.373	1.003732e-05
Tlr4	toll-like receptor 4 [Source:MGI Symbol;Acc:MGI:96824]	−1.377	2.746560e-03
Dusp18	dual specificity phosphatase 18 [Source:MGI Symbol;Acc:MGI:1922469]	−1.377	1.626559e-03
Muc15	mucin 15 [Source:MGI Symbol;Acc:MGI:2442110]	−1.378	2.820613e-03
**Tfcp2l1**	**transcription factor CP2-like 1 [Source:MGI Symbol;Acc:MGI:2444691]**	**−1.381**	**2.758201e-15**
Rusc2	RUN and SH3 domain containing 2 [Source:MGI Symbol;Acc:MGI:2140371]	−1.385	6.198052e-06
Fut9	fucosyltransferase 9 [Source:MGI Symbol;Acc:MGI:1330859]	−1.387	1.382990e-02
Vill	villin-like [Source:MGI Symbol;Acc:MGI:1201781]	−1.387	3.419501e-04
Gm45457	predicted gene 45457 [Source:MGI Symbol;Acc:MGI:5791293]	−1.389	8.149342e-03
Cdc42ep5	CDC42 effector protein (Rho GTPase binding) 5 [Source:MGI Symbol;Acc:MGI:1929745]	−1.398	7.087221e-05
Amt	aminomethyltransferase [Source:MGI Symbol;Acc:MGI:3646700]	−1.399	2.634698e-07

Significant Genes

Runx1 and Runx2 Knock-out

Gene Name	Description	log2 FC	Adj p-value
Fam189a2	family with sequence similarity 189, member A2 [Source:MGI Symbol;Acc:MGI:2685813]	−1.411	9.912146e-03
2210418O10Rik	RIKEN cDNA 2210418O10 gene [Source:MGI Symbol;Acc:MGI:1924208]	−1.414	7.871452e-03
Ngef	neuronal guanine nucleotide exchange factor [Source:MGI Symbol;Acc:MGI:1858414]	−1.416	3.538097e-03
Gjb6	gap junction protein, beta 6 [Source:MGI Symbol;Acc:MGI:107588]	−1.418	1.947963e-03
Lrrc66	leucine rich repeat containing 66 [Source:MGI Symbol;Acc:MGI:2387634]	−1.422	1.021180e-02
**Sorl1**	**sortilin-related receptor, LDLR class A repeats-containing [Source:MGI Symbol;Acc:MGI:1202296]**	**−1.422**	**3.822159e-08**
Acss1	acyl-CoA synthetase short-chain family member 1 [Source:MGI Symbol;Acc:MGI:1915988]	−1.425	3.390610e-03
Hsd17b11	hydroxysteroid (17-beta) dehydrogenase 11 [Source:MGI Symbol;Acc:MGI:2149821]	−1.425	2.023463e-05
Vipr1	vasoactive intestinal peptide receptor 1 [Source:MGI Symbol;Acc:MGI:109272]	−1.428	3.722492e-03
Sorbs3	sorbin and SH3 domain containing 3 [Source:MGI Symbol;Acc:MGI:700013]	−1.428	1.195521e-04
Abcd1	ATP-binding cassette, sub-family D (ALD), member 1 [Source:MGI Symbol;Acc:MGI:1349215]	−1.433	4.454310e-05
Rap1gap2	RAP1 GTPase activating protein 2 [Source:MGI Symbol;Acc:MGI:3028623]	−1.435	1.161859e-02

Significant Genes

Runx1 and Runx2 Knock-out

Gene Name	Description	log2 FC	Adj p-value
Cavin4	caveolae associated 4 [Source:MGI Symbol;Acc:MGI:1915266]	−1.440	6.060334e-04
**Add3**	**adducin 3 (gamma) [Source:MGI Symbol;Acc:MGI:1351615]**	**−1.441**	**2.284080e-12**
Cldn7	claudin 7 [Source:MGI Symbol;Acc:MGI:1859285]	−1.443	4.832186e-03
**Asprv1**	**aspartic peptidase, retroviral-like 1 [Source:MGI Symbol;Acc:MGI:1915105]**	**−1.446**	**1.863128e-04**
Tgm2	transglutaminase 2, C polypeptide [Source:MGI Symbol;Acc:MGI:98731]	−1.449	5.250050e-03
Hpn	hepsin [Source:MGI Symbol;Acc:MGI:1196620]	−1.452	9.553481e-03
Smpd3	sphingomyelin phosphodiesterase 3, neutral [Source:MGI Symbol;Acc:MGI:1927578]	−1.459	1.674764e-04
Tmprss11e	transmembrane protease, serine 11e [Source:MGI Symbol;Acc:MGI:3513175]	−1.462	1.110460e-02
Sorbs2os	sorbin and SH3 domain containing 2, opposite strand [Source:MGI Symbol;Acc:MGI:2443013]	−1.469	1.085948e-02
Kcnn4	potassium intermediate/small conductance calcium-activated channel, subfamily N, member 4 [Source:MGI Symbol;Acc:MGI:1277957]	−1.471	1.545583e-04
Apod	apolipoprotein D [Source:MGI Symbol;Acc:MGI:88056]	−1.475	1.099740e-03
Scin	scinderin [Source:MGI Symbol;Acc:MGI:1306794]	−1.478	4.978234e-04
Hp	haptoglobin [Source:MGI Symbol;Acc:MGI:96211]	−1.480	7.910945e-03

Significant Genes

Runx1 and Runx2 Knock-out

Gene Name	Description	log2 FC	Adj p-value
**Prss8**	**protease, serine 8 (prostasin) [Source:MGI Symbol;Acc:MGI:1923810]**	**−1.482**	**1.357991e-06**
Vsig8	V-set and immunoglobulin domain containing 8 [Source:MGI Symbol;Acc:MGI:3642995]	−1.486	8.229272e-03
Cp	ceruloplasmin [Source:MGI Symbol;Acc:MGI:88476]	−1.486	3.546760e-03
Chst1	carbohydrate sulfotransferase 1 [Source:MGI Symbol;Acc:MGI:1924219]	−1.492	1.055862e-02
Kcnq1	potassium voltage-gated channel, subfamily Q, member 1 [Source:MGI Symbol;Acc:MGI:108083]	−1.493	9.728020e-03
Coro2a	coronin, actin binding protein 2A [Source:MGI Symbol;Acc:MGI:1345966]	−1.496	1.478966e-04
**Myo6**	**myosin VI [Source:MGI Symbol;Acc:MGI:104785]**	**−1.497**	**6.596286e-08**
Hectd2	HECT domain E3 ubiquitin protein ligase 2 [Source:MGI Symbol;Acc:MGI:2442663]	−1.498	7.094633e-05
Kcnd2	potassium voltage-gated channel, Shal-related family, member 2 [Source:MGI Symbol;Acc:MGI:102663]	−1.500	7.972170e-03
Car8	carbonic anhydrase 8 [Source:MGI Symbol;Acc:MGI:88253]	−1.505	7.933649e-03
Map3k21	mitogen-activated protein kinase kinase kinase 21 [Source:MGI Symbol;Acc:MGI:2385307]	−1.506	7.438813e-03
Rasal1	RAS protein activator like 1 (GAP1 like) [Source:MGI Symbol;Acc:MGI:1330842]	−1.507	1.193339e-04

Significant Genes

Runx1 and Runx2 Knock-out

Gene Name	Description	log2 FC	Adj p-value
Cxcl15	chemokine (C-X-C motif) ligand 15 [Source:MGI Symbol;Acc:MGI:1339941]	−1.507	9.914828e-03
Spata13	spermatogenesis associated 13 [Source:MGI Symbol;Acc:MGI:104838]	−1.513	1.867706e-10
Enpp5	ectonucleotide pyrophosphatase/phosphodiesterase 5 [Source:MGI Symbol;Acc:MGI:1933830]	−1.521	8.514715e-03
Wnt7b	wingless-type MMTV integration site family, member 7B [Source:MGI Symbol;Acc:MGI:98962]	−1.522	6.984523e-05
Cdo1	cysteine dioxygenase 1, cytosolic [Source:MGI Symbol;Acc:MGI:105925]	−1.526	5.271068e-03
Klhl13	kelch-like 13 [Source:MGI Symbol;Acc:MGI:1914705]	−1.526	1.734920e-04
Nxf7	nuclear RNA export factor 7 [Source:MGI Symbol;Acc:MGI:2159343]	−1.527	7.438813e-03
Gca	grancalcin [Source:MGI Symbol;Acc:MGI:1918521]	−1.528	3.546760e-03
Ttc28	tetratricopeptide repeat domain 28 [Source:MGI Symbol;Acc:MGI:2140873]	−1.534	6.261253e-06
Vsig10	V-set and immunoglobulin domain containing 10 [Source:MGI Symbol;Acc:MGI:2448533]	−1.547	4.080427e-08
Foxc1	forkhead box C1 [Source:MGI Symbol;Acc:MGI:1347466]	−1.550	1.002671e-05
Grb10	growth factor receptor bound protein 10 [Source:MGI Symbol;Acc:MGI:103232]	−1.552	7.277704e-03
Rassf6	Ras association (RalGDS/AF-6) domain family member 6 [Source:MGI	−1.553	4.925717e-07

Significant Genes			
Runx1 and Runx2 Knock-out			
Gene Name	Description	log2 FC	Adj p-value
	Symbol;Acc:MGI:1920496]		
Lyn	LYN proto-oncogene, Src family tyrosine kinase [Source:MGI Symbol;Acc:MGI:96892]	−1.555	7.187528e-06
Sptlc3	serine palmitoyltransferase, long chain base subunit 3 [Source:MGI Symbol;Acc:MGI:2444678]	−1.559	3.339018e-03
Gm34006	predicted gene, 34006 [Source:MGI Symbol;Acc:MGI:5593165]	−1.560	7.588989e-03
Gm31497	predicted gene, 31497 [Source:MGI Symbol;Acc:MGI:5590656]	−1.561	2.021045e-03
Krt33b	keratin 33B [Source:MGI Symbol;Acc:MGI:1309991]	−1.564	6.339128e-03
Tspan33	tetraspanin 33 [Source:MGI Symbol;Acc:MGI:1919012]	−1.566	2.737921e-03
Tmc4	transmembrane channel-like gene family 4 [Source:MGI Symbol;Acc:MGI:2669035]	−1.567	3.100578e-07
Krt83	keratin 83 [Source:MGI Symbol;Acc:MGI:3690448]	−1.569	7.116385e-03
Runx3	runt related transcription factor 3 [Source:MGI Symbol;Acc:MGI:102672]	−1.573	1.693207e-04
Wnt8a	wingless-type MMTV integration site family, member 8A [Source:MGI Symbol;Acc:MGI:107924]	−1.574	3.118884e-05
Pyroxd2	pyridine nucleotide-disulphide oxidoreductase domain 2 [Source:MGI Symbol;Acc:MGI:1921830]	−1.578	7.629189e-03
Prss23	protease, serine 23 [Source:MGI Symbol;Acc:MGI:1923703]	−1.578	6.054113e-04

Significant Genes

Runx1 and Runx2 Knock-out

Gene Name	Description	log2 FC	Adj p-value
Six4	sine oculis-related homeobox 4 [Source:MGI Symbol;Acc:MGI:106034]	−1.580	8.249932e-04
Mmp15	matrix metallopeptidase 15 [Source:MGI Symbol;Acc:MGI:109320]	−1.581	1.693207e-04
Timp1	tissue inhibitor of metalloproteinase 1 [Source:MGI Symbol;Acc:MGI:98752]	−1.581	2.230621e-04
Slc44a3	solute carrier family 44, member 3 [Source:MGI Symbol;Acc:MGI:2384860]	−1.582	1.849490e-03
Ano1	anoctamin 1, calcium activated chloride channel [Source:MGI Symbol;Acc:MGI:2142149]	−1.591	7.085753e-05
**Rhpn2**	**rhophilin, Rho GTPase binding protein 2 [Source:MGI Symbol;Acc:MGI:1289234]**	**−1.592**	**2.882604e-06**
Armcx4	armadillo repeat containing, X-linked 4 [Source:MGI Symbol;Acc:MGI:2147887]	−1.597	7.351540e-09
C3	complement component 3 [Source:MGI Symbol;Acc:MGI:88227]	−1.601	2.782138e-03
Slc9a3r2	solute carrier family 9 (sodium/hydrogen exchanger), member 3 regulator 2 [Source:MGI Symbol;Acc:MGI:1890662]	−1.603	8.454701e-06
Padi2	peptidyl arginine deiminase, type II [Source:MGI Symbol;Acc:MGI:1338892]	−1.605	3.685778e-03
Slain1	SLAIN motif family, member 1 [Source:MGI Symbol;Acc:MGI:2145578]	−1.607	2.896462e-08
Hey1	hairy/enhancer-of-split related with YRPW motif 1 [Source:MGI Symbol;Acc:MGI:1341800]	−1.610	3.394981e-05

Significant Genes

Runx1 and Runx2 Knock-out

Gene Name	Description	log2 FC	Adj p-value
Fxyd2	FXYD domain-containing ion transport regulator 2 [Source:MGI Symbol;Acc:MGI:1195260]	−1.611	6.742589e-03
Rftn1	raftlin lipid raft linker 1 [Source:MGI Symbol;Acc:MGI:1923688]	−1.612	1.597544e-04
Cck	cholecystokinin [Source:MGI Symbol;Acc:MGI:88297]	−1.616	6.571431e-03
Syne4	spectrin repeat containing, nuclear envelope family member 4 [Source:MGI Symbol;Acc:MGI:2141950]	−1.618	2.532275e-03
Ptx3	pentraxin related gene [Source:MGI Symbol;Acc:MGI:104641]	−1.621	3.640770e-04
Rhoj	ras homolog family member J [Source:MGI Symbol;Acc:MGI:1931551]	−1.623	2.605698e-03
Plscr2	phospholipid scramblase 2 [Source:MGI Symbol;Acc:MGI:1270860]	−1.624	3.029633e-03
Scn5a	sodium channel, voltage-gated, type V, alpha [Source:MGI Symbol;Acc:MGI:98251]	−1.624	4.703884e-03
Bik	BCL2-interacting killer [Source:MGI Symbol;Acc:MGI:1206591]	−1.629	4.203134e-04
Papss2	3'-phosphoadenosine 5'-phosphosulfate synthase 2 [Source:MGI Symbol;Acc:MGI:1330223]	−1.629	1.514514e-04
Gm266	predicted gene 266 [Source:MGI Symbol;Acc:MGI:2685112]	−1.631	2.311419e-03
**Tpcn1**	**two pore channel 1 [Source:MGI Symbol;Acc:MGI:2182472]**	**−1.635**	**2.416081e-20**

Significant Genes

Runx1 and Runx2 Knock-out

Gene Name	Description	log2 FC	Adj p-value
Meltf	melanotransferrin [Source:MGI Symbol;Acc:MGI:1353421]	−1.642	2.851097e-03
Prelp	proline arginine-rich end leucine-rich repeat [Source:MGI Symbol;Acc:MGI:2151110]	−1.643	2.023008e-06
Slco1a5	solute carrier organic anion transporter family, member 1a5 [Source:MGI Symbol;Acc:MGI:1351865]	−1.657	4.035980e-03
Arid3b	AT rich interactive domain 3B (BRIGHT-like) [Source:MGI Symbol;Acc:MGI:1930768]	−1.660	1.786255e-06
Loxl4	lysyl oxidase-like 4 [Source:MGI Symbol;Acc:MGI:1914823]	−1.662	1.759769e-04
Rgl3	ral guanine nucleotide dissociation stimulator-like 3 [Source:MGI Symbol;Acc:MGI:1918996]	−1.667	2.902040e-03
Csdc2	cold shock domain containing C2, RNA binding [Source:MGI Symbol;Acc:MGI:2146027]	−1.668	4.180610e-04
Piezo2	piezo-type mechanosensitive ion channel component 2 [Source:MGI Symbol;Acc:MGI:1918781]	−1.668	2.847002e-08
Apol7a	apolipoprotein L 7a [Source:MGI Symbol;Acc:MGI:1923011]	−1.669	3.366478e-03
Nos1ap	nitric oxide synthase 1 (neuronal) adaptor protein [Source:MGI Symbol;Acc:MGI:1917979]	−1.671	4.447918e-11
Tjp3	tight junction protein 3 [Source:MGI Symbol;Acc:MGI:1351650]	−1.672	4.595029e-06

Significant Genes

Runx1 and Runx2 Knock-out

Gene Name	Description	log2 FC	Adj p-value
Esr1	estrogen receptor 1 (alpha) [Source:MGI Symbol;Acc:MGI:1352467]	−1.672	7.206924e-04
Ly6g6g	lymphocyte antigen 6 complex, locus G6G [Source:MGI Symbol;Acc:MGI:1925975]	−1.677	3.399798e-03
Mxra7	matrix-remodelling associated 7 [Source:MGI Symbol;Acc:MGI:1914872]	−1.680	1.222176e-03
Fam221a	family with sequence similarity 221, member A [Source:MGI Symbol;Acc:MGI:2442161]	−1.681	1.086418e-03
Anxa9	annexin A9 [Source:MGI Symbol;Acc:MGI:1923711]	−1.681	6.262628e-05
Rftn2	raftlin family member 2 [Source:MGI Symbol;Acc:MGI:1921263]	−1.681	1.737920e-03
Hs3st1	heparan sulfate (glucosamine) 3-O-sulfotransferase 1 [Source:MGI Symbol;Acc:MGI:1201606]	−1.683	3.301900e-04
Cracr2b	calcium release activated channel regulator 2B [Source:MGI Symbol;Acc:MGI:2446129]	−1.683	3.118884e-05
Gm16485	predicted gene 16485 [Source:MGI Symbol;Acc:MGI:3642771]	−1.687	1.103697e-04
Mansc1	MANSC domain containing 1 [Source:MGI Symbol;Acc:MGI:1914979]	−1.689	4.040026e-06
Mfge8	milk fat globule-EGF factor 8 protein [Source:MGI Symbol;Acc:MGI:102768]	−1.696	2.311419e-03
Gm39556	predicted gene, 39556 [Source:MGI Symbol;Acc:MGI:5622441]	−1.702	3.567785e-04
Krt8-ps	keratin 8, pseudogene [Source:MGI Symbol;Acc:MGI:3779503]	−1.708	9.631096e-04

Significant Genes

Runx1 and Runx2 Knock-out

Gene Name	Description	log2 FC	Adj p-value
Gne	glucosamine (UDP-N-acetyl)-2-epimerase/N-acetylmannosamine kinase [Source:MGI Symbol;Acc:MGI:1354951]	−1.710	7.483355e-06
Tm4sf1	transmembrane 4 superfamily member 1 [Source:MGI Symbol;Acc:MGI:104678]	−1.712	2.044069e-04
Tmprss2	transmembrane protease, serine 2 [Source:MGI Symbol;Acc:MGI:1354381]	−1.715	1.778765e-05
Krt8	keratin 8 [Source:MGI Symbol;Acc:MGI:96705]	−1.716	1.144486e-03
Anpep	alanyl (membrane) aminopeptidase [Source:MGI Symbol;Acc:MGI:5000466]	−1.717	8.423561e-06
Iqgap2	IQ motif containing GTPase activating protein 2 [Source:MGI Symbol;Acc:MGI:2449975]	−1.718	2.000539e-04
Gm12305	predicted gene 12305 [Source:MGI Symbol;Acc:MGI:3650835]	−1.726	3.567785e-04
Pgr	progesterone receptor [Source:MGI Symbol;Acc:MGI:97567]	−1.729	5.698601e-09
Pced1b	PC-esterase domain containing 1B [Source:MGI Symbol;Acc:MGI:2446270]	−1.730	6.092663e-09
Crhr1	corticotropin releasing hormone receptor 1 [Source:MGI Symbol;Acc:MGI:88498]	−1.735	1.185912e-04
Pdk4	pyruvate dehydrogenase kinase, isoenzyme 4 [Source:MGI Symbol;Acc:MGI:1351481]	−1.743	1.123574e-03
Pkp2	plakophilin 2 [Source:MGI Symbol;Acc:MGI:1914701]	−1.744	1.150478e-05
Krt87	keratin 87 [Source:MGI Symbol;Acc:MGI:3665486]	−1.747	3.187871e-03

Significant Genes

Runx1 and Runx2 Knock-out

Gene Name	Description	log2 FC	Adj p-value
Cryba4	crystallin, beta A4 [Source:MGI Symbol;Acc:MGI:102716]	−1.748	5.891012e-06
Gm9967	predicted gene 9967 [Source:MGI Symbol;Acc:MGI:3704300]	−1.754	2.368980e-03
Plekhf1	pleckstrin homology domain containing, family F (with FYVE domain) member 1 [Source:MGI Symbol;Acc:MGI:1919537]	−1.762	5.528020e-05
Col6a2	collagen, type VI, alpha 2 [Source:MGI Symbol;Acc:MGI:88460]	−1.770	9.390584e-06
Ccser1	coiled-coil serine rich 1 [Source:MGI Symbol;Acc:MGI:3045354]	−1.772	4.040409e-05
Enpp3	ectonucleotide pyrophosphatase/phosphodiesterase 3 [Source:MGI Symbol;Acc:MGI:2143702]	−1.781	1.781270e-04
Gm42778	predicted gene 42778 [Source:MGI Symbol;Acc:MGI:5662915]	−1.784	2.900854e-05
Arhgef6	Rac/Cdc42 guanine nucleotide exchange factor (GEF) 6 [Source:MGI Symbol;Acc:MGI:1920591]	−1.786	3.630199e-05
Arhgef38	Rho guanine nucleotide exchange factor (GEF) 38 [Source:MGI Symbol;Acc:MGI:1924919]	−1.786	5.779995e-04
Krt31	keratin 31 [Source:MGI Symbol;Acc:MGI:1309993]	−1.787	3.304296e-03
Cldn10	claudin 10 [Source:MGI Symbol;Acc:MGI:1913101]	−1.788	1.131051e-03
Itgb3	integrin beta 3 [Source:MGI Symbol;Acc:MGI:96612]	−1.794	3.526224e-05

Significant Genes

Runx1 and Runx2 Knock-out

Gene Name	Description	log2 FC	Adj p-value
Mgll	monoglyceride lipase [Source:MGI Symbol;Acc:MGI:1346042]	−1.799	2.321218e-06
Myzap	myocardial zonula adherens protein [Source:MGI Symbol;Acc:MGI:2142908]	−1.801	1.412696e-05
Krt26	keratin 26 [Source:MGI Symbol;Acc:MGI:2444913]	−1.804	9.709086e-05
Krt18	keratin 18 [Source:MGI Symbol;Acc:MGI:96692]	−1.806	6.244092e-04
Slc5a8	solute carrier family 5 (iodide transporter), member 8 [Source:MGI Symbol;Acc:MGI:2384916]	−1.807	2.406212e-05
**Spns2**	**spinster homolog 2 [Source:MGI Symbol;Acc:MGI:2384936]**	**−1.808**	**1.629648e-16**
Angpt1	angiopoietin 1 [Source:MGI Symbol;Acc:MGI:108448]	−1.809	2.486031e-03
Idi2	isopentenyl-diphosphate delta isomerase 2 [Source:MGI Symbol;Acc:MGI:2444315]	−1.817	2.820613e-03
Reep6	receptor accessory protein 6 [Source:MGI Symbol;Acc:MGI:1917585]	−1.819	3.290323e-11
Gm44005	predicted gene, 44005 [Source:MGI Symbol;Acc:MGI:5690397]	−1.822	1.232482e-03
Pip5k1b	phosphatidylinositol-4-phosphate 5-kinase, type 1 beta [Source:MGI Symbol;Acc:MGI:107930]	−1.824	8.628213e-04
Col6a1	collagen, type VI, alpha 1 [Source:MGI Symbol;Acc:MGI:88459]	−1.825	9.585444e-06
Plekha6	pleckstrin homology domain containing, family A member 6 [Source:MGI	−1.825	7.206957e-08

Significant Genes

Runx1 and Runx2 Knock-out

Gene Name	Description	log2 FC	Adj p-value
	Symbol;Acc:MGI:2388662]		
Podxl	podocalyxin-like [Source:MGI Symbol;Acc:MGI:1351317]	−1.826	3.247395e-07
Vtcn1	V-set domain containing T cell activation inhibitor 1 [Source:MGI Symbol;Acc:MGI:3039619]	−1.828	1.160711e-05
Bcl2l14	BCL2-like 14 (apoptosis facilitator) [Source:MGI Symbol;Acc:MGI:1914063]	−1.834	1.014495e-04
Muc4	mucin 4 [Source:MGI Symbol;Acc:MGI:2153525]	−1.840	2.039745e-04
**Dsc2**	**desmocollin 2 [Source:MGI Symbol;Acc:MGI:103221]**	**−1.842**	**5.611943e-09**
Rorc	RAR-related orphan receptor gamma [Source:MGI Symbol;Acc:MGI:104856]	−1.847	2.148731e-04
Clmn	calmin [Source:MGI Symbol;Acc:MGI:2136957]	−1.850	5.845930e-13
Ido1	indoleamine 2,3-dioxygenase 1 [Source:MGI Symbol;Acc:MGI:96416]	−1.857	2.378962e-03
**Cobl**	**cordon-bleu WH2 repeat [Source:MGI Symbol;Acc:MGI:105056]**	**−1.859**	**1.095748e-15**
**Gm14137**	**predicted gene 14137 [Source:MGI Symbol;Acc:MGI:3651144]**	**−1.868**	**3.259205e-07**
Rnf223	ring finger 223 [Source:MGI Symbol;Acc:MGI:3588193]	−1.876	1.244573e-03
Aldh1a3	aldehyde dehydrogenase family 1, subfamily A3 [Source:MGI Symbol;Acc:MGI:1861722]	−1.876	3.353648e-05
Myo5c	myosin VC [Source:MGI Symbol;Acc:MGI:2442485]	−1.881	2.644135e-04

Significant Genes

Runx1 and Runx2 Knock-out

Gene Name	Description	log2 FC	Adj p-value
Ildr1	immunoglobulin-like domain containing receptor 1 [Source:MGI Symbol;Acc:MGI:2146574]	−1.883	7.293698e-08
Arhgef28	Rho guanine nucleotide exchange factor (GEF) 28 [Source:MGI Symbol;Acc:MGI:1346016]	−1.883	1.472740e-06
Krt19	keratin 19 [Source:MGI Symbol;Acc:MGI:96693]	−1.894	2.103830e-04
Trim46	tripartite motif-containing 46 [Source:MGI Symbol;Acc:MGI:2673000]	−1.899	5.783935e-06
Ptpn18	protein tyrosine phosphatase, non-receptor type 18 [Source:MGI Symbol;Acc:MGI:108410]	−1.901	1.663633e-04
Slc7a2	solute carrier family 7 (cationic amino acid transporter, y+ system), member 2 [Source:MGI Symbol;Acc:MGI:99828]	−1.905	2.488605e-06
Tspan8	tetraspanin 8 [Source:MGI Symbol;Acc:MGI:2384918]	−1.910	3.342458e-05
Mall	mal, T cell differentiation protein-like [Source:MGI Symbol;Acc:MGI:2385152]	−1.916	6.744924e-05
Prr15l	proline rich 15-like [Source:MGI Symbol;Acc:MGI:2387599]	−1.918	1.127956e-04
Atp10b	ATPase, class V, type 10B [Source:MGI Symbol;Acc:MGI:2442688]	−1.924	3.476059e-06
Spink6	serine peptidase inhibitor, Kazal type 6 [Source:MGI Symbol;Acc:MGI:3648654]	−1.929	9.744467e-05
Aqp5	aquaporin 5 [Source:MGI Symbol;Acc:MGI:106215]	−1.931	1.693207e-04
Gja3	gap junction protein, alpha 3 [Source:MGI Symbol;Acc:MGI:95714]	−1.935	9.709086e-05

Significant Genes

Runx1 and Runx2 Knock-out

Gene Name	Description	log2 FC	Adj p-value
Scnn1b	sodium channel, nonvoltage-gated 1 beta [Source:MGI Symbol;Acc:MGI:104696]	−1.936	1.035524e-06
Cytip	cytohesin 1 interacting protein [Source:MGI Symbol;Acc:MGI:2183535]	−1.936	6.946419e-06
Klk14	kallikrein related-peptidase 14 [Source:MGI Symbol;Acc:MGI:2447564]	−1.937	1.206183e-03
Ppp1r9a	protein phosphatase 1, regulatory subunit 9A [Source:MGI Symbol;Acc:MGI:2442401]	−1.937	2.399599e-04
Plag1	pleiomorphic adenoma gene 1 [Source:MGI Symbol;Acc:MGI:1891916]	−1.944	5.993891e-06
Hhipl2	hedgehog interacting protein-like 2 [Source:MGI Symbol;Acc:MGI:1926022]	−1.953	2.139259e-04
Papln	papilin, proteoglycan-like sulfated glycoprotein [Source:MGI Symbol;Acc:MGI:2386139]	−1.954	9.431838e-10
Cadps2	Ca2+-dependent activator protein for secretion 2 [Source:MGI Symbol;Acc:MGI:2443963]	−1.960	1.300785e-10
Arhgap40	Rho GTPase activating protein 40 [Source:MGI Symbol;Acc:MGI:3649852]	−1.974	1.758498e-05
Muc1	mucin 1, transmembrane [Source:MGI Symbol;Acc:MGI:97231]	−1.985	8.918491e-07
Ntn1	netrin 1 [Source:MGI Symbol;Acc:MGI:105088]	−2.006	3.047637e-08
Scml4	Scm polycomb group protein like 4 [Source:MGI Symbol;Acc:MGI:2446140]	−2.022	1.051624e-03
Plce1	phospholipase C, epsilon 1 [Source:MGI Symbol;Acc:MGI:1921305]	−2.037	9.539359e-06

Significant Genes

Runx1 and Runx2 Knock-out

Gene Name	Description	log2 FC	Adj p-value
Ly6g6d	lymphocyte antigen 6 complex, locus G6D [Source:MGI Symbol;Acc:MGI:2148931]	−2.042	2.644135e-04
Ccdc33	coiled-coil domain containing 33 [Source:MGI Symbol;Acc:MGI:1922464]	−2.048	8.410631e-04
Pdlim3	PDZ and LIM domain 3 [Source:MGI Symbol;Acc:MGI:1859274]	−2.053	1.235645e-04
Adamts3	a disintegrin-like and metallopeptidase (reprolysin type) with thrombospondin type 1 motif, 3 [Source:MGI Symbol;Acc:MGI:3045353]	−2.055	2.735110e-06
Galnt15	polypeptide N-acetylgalactosaminyltransferase 15 [Source:MGI Symbol;Acc:MGI:1926004]	−2.079	1.016233e-05
S100a7a	S100 calcium binding protein A7A [Source:MGI Symbol;Acc:MGI:2687194]	−2.085	2.362992e-04
Igfbp5	insulin-like growth factor binding protein 5 [Source:MGI Symbol;Acc:MGI:96440]	−2.085	1.801493e-05
Tlr5	toll-like receptor 5 [Source:MGI Symbol;Acc:MGI:1858171]	−2.087	2.481307e-06
BC006965	cDNA sequence BC006965 [Source:MGI Symbol;Acc:MGI:2384955]	−2.088	4.588699e-10
Btn1a1	butyrophilin, subfamily 1, member A1 [Source:MGI Symbol;Acc:MGI:103118]	−2.095	2.785861e-04
Calhm4	calcium homeostasis modulator family member 4 [Source:MGI Symbol;Acc:MGI:2685489]	−2.100	3.297304e-04
Elmod1	ELMO/CED-12 domain containing 1 [Source:MGI Symbol;Acc:MGI:3583900]	−2.101	1.332784e-04

Significant Genes

Runx1 and Runx2 Knock-out

Gene Name	Description	log2 FC	Adj p-value
Slc5a5	solute carrier family 5 (sodium iodide symporter), member 5 [Source:MGI Symbol;Acc:MGI:2149330]	−2.102	7.187528e-06
Prss27	protease, serine 27 [Source:MGI Symbol;Acc:MGI:2450123]	−2.107	4.666980e-04
Sorbs2	sorbin and SH3 domain containing 2 [Source:MGI Symbol;Acc:MGI:1924574]	−2.129	3.622333e-09
Tmprss11d	transmembrane protease, serine 11d [Source:MGI Symbol;Acc:MGI:2385221]	−2.148	9.631096e-04
Ceacam1	carcinoembryonic antigen-related cell adhesion molecule 1 [Source:MGI Symbol;Acc:MGI:1347245]	−2.149	1.108408e-06
Dnase1l2	deoxyribonuclease 1-like 2 [Source:MGI Symbol;Acc:MGI:1913955]	−2.157	1.481933e-06
Btla	B and T lymphocyte associated [Source:MGI Symbol;Acc:MGI:2658978]	−2.185	7.356918e-04
Shank2	SH3 and multiple ankyrin repeat domains 2 [Source:MGI Symbol;Acc:MGI:2671987]	−2.211	7.837365e-15
Gm7694	predicted gene 7694 [Source:MGI Symbol;Acc:MGI:3649135]	−2.225	1.561663e-10
Trpm6	transient receptor potential cation channel, subfamily M, member 6 [Source:MGI Symbol;Acc:MGI:2675603]	−2.232	7.224445e-08
Elf5	E74-like factor 5 [Source:MGI Symbol;Acc:MGI:1335079]	−2.234	2.201587e-07
Gm11571	predicted gene 11571 [Source:MGI Symbol;Acc:MGI:3650087]	−2.245	5.695456e-05

Significant Genes

Runx1 and Runx2 Knock-out

Gene Name	Description	log2 FC	Adj p-value
Atg9b	autophagy related 9B [Source:MGI Symbol;Acc:MGI:2685420]	−2.263	1.561663e-10
Smoc1	SPARC related modular calcium binding 1 [Source:MGI Symbol;Acc:MGI:1929878]	−2.264	2.551862e-06
Rasef	RAS and EF hand domain containing [Source:MGI Symbol;Acc:MGI:2448565]	−2.276	1.318975e-08
S100g	S100 calcium binding protein G [Source:MGI Symbol;Acc:MGI:104528]	−2.285	2.391209e-05
Gabrp	gamma-aminobutyric acid (GABA) A receptor, pi [Source:MGI Symbol;Acc:MGI:2387597]	−2.304	1.401765e-07
Mlph	melanophilin [Source:MGI Symbol;Acc:MGI:2176380]	−2.308	2.532074e-12
Prlr	prolactin receptor [Source:MGI Symbol;Acc:MGI:97763]	−2.314	4.699773e-06
Nccrp1	non-specific cytotoxic cell receptor protein 1 homolog (zebrafish) [Source:MGI Symbol;Acc:MGI:2685009]	−2.315	4.787025e-04
Foxi1	forkhead box I1 [Source:MGI Symbol;Acc:MGI:1096329]	−2.340	6.841659e-05
Wnt5b	wingless-type MMTV integration site family, member 5B [Source:MGI Symbol;Acc:MGI:98959]	−2.355	3.357679e-07
Muc20	mucin 20 [Source:MGI Symbol;Acc:MGI:2385039]	−2.356	7.905778e-09
Ccn5	cellular communication network factor 5 [Source:MGI Symbol;Acc:MGI:1328326]	−2.365	1.711324e-04
Alpl	alkaline phosphatase, liver/bone/kidney [Source:MGI Symbol;Acc:MGI:87983]	−2.380	2.596887e-12

Significant Genes

Runx1 and Runx2 Knock-out

Gene Name	Description	log2 FC	Adj p-value
Krt33a	keratin 33A [Source:MGI Symbol;Acc:MGI:1919138]	−2.386	2.393177e-04
Unc5c	unc-5 netrin receptor C [Source:MGI Symbol;Acc:MGI:1095412]	−2.393	2.207909e-07
Padi4	peptidyl arginine deiminase, type IV [Source:MGI Symbol;Acc:MGI:1338898]	−2.394	5.644759e-18
Ltbp2	latent transforming growth factor beta binding protein 2 [Source:MGI Symbol;Acc:MGI:99502]	−2.424	1.623276e-13
**Krt7**	**keratin 7 [Source:MGI Symbol;Acc:MGI:96704]**	**−2.430**	**1.248109e-10**
Pik3cd	phosphatidylinositol-4,5-bisphosphate 3-kinase catalytic subunit delta [Source:MGI Symbol;Acc:MGI:1098211]	−2.433	4.066095e-11
Hephl1	hephaestin-like 1 [Source:MGI Symbol;Acc:MGI:2685355]	−2.465	1.853220e-05
Tlcd4	TLC domain containing 4 [Source:MGI Symbol;Acc:MGI:1923195]	−2.508	6.236086e-07
Krtap8-1	keratin associated protein 8-1 [Source:MGI Symbol;Acc:MGI:1330293]	−2.509	1.967812e-04
Rspo1	R-spondin 1 [Source:MGI Symbol;Acc:MGI:2183426]	−2.526	1.681694e-05
Gm13546	predicted gene 13546 [Source:MGI Symbol;Acc:MGI:3649666]	−2.563	4.723511e-06
Kit	KIT proto-oncogene receptor tyrosine kinase [Source:MGI Symbol;Acc:MGI:96677]	−2.585	3.940565e-12
Scara5	scavenger receptor class A, member 5 [Source:MGI Symbol;Acc:MGI:1918395]	−2.609	1.576601e-06

Significant Genes

Runx1 and Runx2 Knock-out

Gene Name	Description	log2 FC	Adj p-value
Gpx3	glutathione peroxidase 3 [Source:MGI Symbol;Acc:MGI:105102]	−2.665	3.725975e-06
Gprc5d	G protein-coupled receptor, family C, group 5, member D [Source:MGI Symbol;Acc:MGI:1935037]	−2.695	7.372598e-06
Klhdc7a	kelch domain containing 7A [Source:MGI Symbol;Acc:MGI:2444612]	−2.716	7.976874e-09
**Mme**	**membrane metallo endopeptidase [Source:MGI Symbol;Acc:MGI:97004]**	**−2.741**	**2.176045e-13**
Gm12347	predicted gene 12347 [Source:MGI Symbol;Acc:MGI:3758174]	−2.800	1.237137e-06
**Mal**	**myelin and lymphocyte protein, T cell differentiation protein [Source:MGI Symbol;Acc:MGI:892970]**	**−2.898**	**6.362003e-10**
Krt78	keratin 78 [Source:MGI Symbol;Acc:MGI:1917529]	−3.010	2.023008e-06
**Atp6v1b1**	**ATPase, H+ transporting, lysosomal V1 subunit B1 [Source:MGI Symbol;Acc:MGI:103285]**	**−3.093**	**1.994906e-13**
Dynap	dynactin associated protein [Source:MGI Symbol;Acc:MGI:1922827]	−3.164	2.023992e-05
Sox14	SRY (sex determining region Y)-box 14 [Source:MGI Symbol;Acc:MGI:98362]	−3.342	4.384480e-11
Krtap7-1	keratin associated protein 7-1 [Source:MGI Symbol;Acc:MGI:1918613]	−3.494	2.241750e-06
Itprid1	ITPR interacting domain containing 1 [Source:MGI Symbol;Acc:MGI:2685304]	−3.596	4.647874e-12

Significant Genes

Runx1 and Runx2 Knock-out

Gene Name	Description	log2 FC	Adj p-value
**Cnfn**	**cornifelin [Source:MGI Symbol;Acc:MGI:1919633]**	**−3.714**	**6.195864e-09**
**Rptn**	**repetin [Source:MGI Symbol;Acc:MGI:1099055]**	**−4.790**	**2.170179e-08**
